# Uracil-Containing Heterodimers of a New Type: Synthesis and Study of Their Anti-Viral Properties

**DOI:** 10.3390/molecules25153350

**Published:** 2020-07-23

**Authors:** Anna A. Maslova, Elena S. Matyugina, Robert Snoeck, Graciela Andrei, Sergey N. Kochetkov, Anastasia L. Khandazhinskaya, Mikhail S. Novikov

**Affiliations:** 1 Engelhardt Institute of Molecular Biology, Russian Academy of Science, 119991 Moscow, Russia; maslova_anna94@mail.ru (A.A.M.); matyugina@gmail.com (E.S.M.); snk1952@gmail.com (S.N.K.); 2 Rega Institute for Medical Research, KU Leuven, 3000 Leuven, Belgium; robert.snoeck@kuleuven.be (R.S.); graciela.andrei@kuleuven.be (G.A.); 3 Department of Pharmaceutical & Toxicological Chemistry, Volgograd State Medical University, 400131 Volgograd, Russia; m-novikov1@mail.ru

**Keywords:** HIV, HSV-2, CMV, co-infection, prodrug, dual activity

## Abstract

Widespread latent herpes viral infections within a population can lead to the development of co-infections in HIV-infected patients. These infections are not particularly dangerous for healthy individuals and often occur with minimal symptoms, but for those who are immunocompromised, these infections can accelerate the acute phase of HIV infection and AIDS. Thus, the idea of designing compounds that could combine activity against HIV and co-infections would seem promising. In that regard, eleven compounds were synthesized that represent conjugates of non-nucleoside HIV reverse transcriptase inhibitors and nucleoside inhibitors of the herpes family viruses with the hope that these novel heterodimers will result in dual activity against HIV and concomitant herpes virus infections.

## 1. Introduction

According to the WHO, there are more than 38 million people currently living with human immunodeficiency virus (HIV) and of those, about 3 million HIV-infected people die each year. Thus, HIV infection and the AIDS pandemic are not only a serious threat to human health, but can also have significant social and economic consequences. Currently used highly active antiretroviral therapy (HAART) is not a panacea, since long-term side effects, the development of drug resistance, the emergence of resistant strains of the virus, as well as intolerance by some patients for these drugs have been problematic [1]. An additional problem is the simultaneous infection of other viruses, such as hepatitis, cytomegalovirus, and herpes viruses among others.

Herpes simplex virus type 2 (HSV-2) is one of the most common sexually transmitted infections worldwide and 50–90% of HIV-infected people are co-infected with this virus [2]. HSV-2 is a DNA virus belonging to the herpes virus family, which includes more than 120 viruses that infect many animal species. Primary infection with herpes viruses in immunocompetent hosts is often asymptomatic or with minimal symptoms, but morbidity and mortality can be high in the case of a person who is immunodeficient, especially in the case of cytomegalovirus (CMV), herpes simplex viruses and Varicella-Zoster virus (VZV) [3]. The immunological effect of viral co-infections contributes to the acceleration of virus replication, viral genotypic heterogeneity and a decrease in CD4^+^ T-lymphocytes, which leads to a weakening of the immune system, reduced survival and significantly increases the risk of HIV-1 transmission [4,5,6]. Long-term epidemiological and molecular studies have shown a strong and synergistic relationship between HSV-2 and HIV-1 infections [6,7]. Thus, there is obvious need to create an effective antiviral drug against both viruses.

Previously, we have synthesized heterodimers of 2′,3′-dideoxy-3′-azidothymidine, a classical nucleoside inhibitor (NI) of DNA biosynthesis catalyzed by HIV reverse transcriptase, and 1-[ω-(4-bromophenoxy)alkyl] uracil derivatives which are non-nucleoside inhibitors of HCMV replication [8]. We have shown that such heterodimers are able to release active antiviral components under the action of hydrolyzing enzymes (esterases). The obtained compounds demonstrated antiviral activity in vitro against CMV (ID_50_ 3–12 μM) and HIV-1 (ID_50_ 0.19–0.83 μM) without cytotoxicity (CD_50_ 170–600 μM in a culture of human lung embryo diploid fibroblasts, CD_50_ > 100 µM on MT-4 cells). Experiments on the human tissue system ex vivo simultaneously infected with HIV-1 (LAI.04) and CMV confirmed the ability of the compounds to completely suppress the replication of both viruses in concentration of 10 μM, with a complete absence of cytotoxicity at this concentration [9].

**Figure 1:**
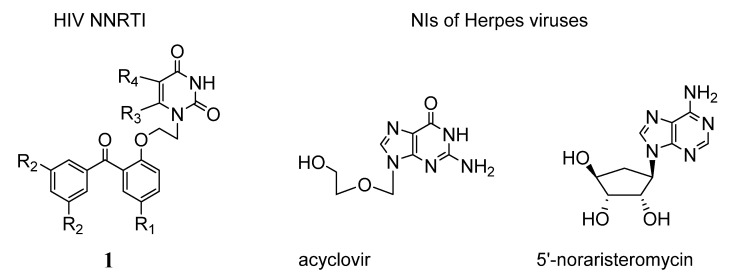
Lead antiviral agents.

Here we present the synthesis and activity of two new groups of heterodimers. These heterodimers consist of a non-nucleoside HIV reverse transcriptase inhibitor (HIV NNRTI), containing the substituted benzophenone residue attached to the N-1 position of uracil by an oxyethyl linker [10,11,12] with nucleoside analogues (NIs) that have significant activity against herpes viruses (CMV, VZV, HSV-2), acyclovir (ACV) or 5′-noraristeromycin (Figure 1).

A study of the biological activity of N^1^-alkylated uracil derivatives **1** containing the benzophenone fragment showed potent antiviral activity against HIV-1 (EC_50_ 0.016–0.51 μM on MT-4 cells) [10,12]. A structure-activity relationship study revealed that the R_1_ substituent plays a crucial role in modulating biological activity. The R_2_ substituents also have positive influence on the activity of the compounds. Thus, the most active uracil derivatives with R_1_ = H, Cl or Br and R_2_ = H, Me or Cl were selected to design heterodimers.

**Figure 2:**
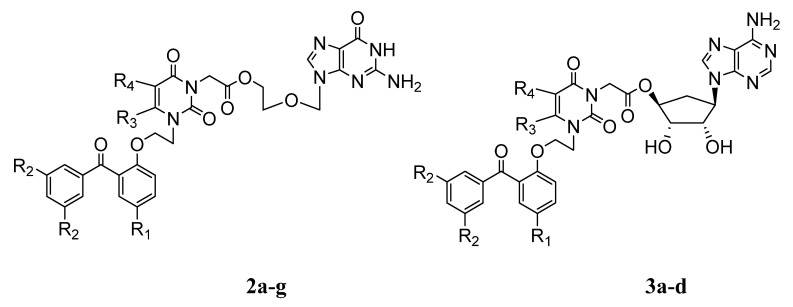
New heterodimers.

The first group of heterodimers (**2a-2g**) was based on ACV, which is an FDA-approved drug for the treatment of HSV, and also has some activity against Varicella-Zoster virus (Figure 2). ACV is an acyclic analogue of guanosine, which, after intracellular conversion to the corresponding triphosphate, becomes a chain terminator substrate of the virus DNA polymerase [13]. There are three stages of ACV phosphorylation. The first stage is catalyzed by herpes virus thymidine kinase (HSV-TK), which recognizes ACV as a substrate much more efficiently than cell thymidine kinase. Differences in substrate specificity of cell and viral thymidine kinases provide high selectivity for ACV to the virus. VZV thymidine kinase can also phosphorylate ACV [13]. Phosphorylation of ACV monophosphate to diphosphate by the cell enzyme guanylate kinase proceeds at a lower rate than the conversion of GMP to GDP, however, the reaction proceeds quite efficiently, and most of the monophosphate is converted to the diphosphate. The last phosphorylation step can be carried out by several different cellular enzymes, including phosphoglycerate kinase, nucleoside diphosphate kinase and phosphoenolpyruvate kinase [13]. The final ACV triphosphate is the actual active form of the compound. Viral DNA polymerase incorporates it into viral DNA instead of 2’-deoxyguanosine triphosphate, which then causes chain termination. Interestingly, treatment of HIV-1/HSV-2 co-infected people with ACV reduces the viral load of both viruses, although suppression of HIV-1 by ACV is not particularly effective [14].

The second group of heterodimers (**3a-3d)** incorporates 5′-noraristeromycin, which is a carbocyclic analogue of adenosine (Figure 2). 5′-Noraristeromycin doesn’t have the methylene group at the 5′-position and, as a consequence, cannot be phosphorylated by intracellular enzymes. As it is an effective inhibitor of the cellular enzyme S-adenosylhomocysteine hydrolase, 5′-noraristeromycin has a wide spectrum of antiviral activity against DNA and RNA viruses, including herpes viruses [15]. Racemic (±)-5′-noraristeromycin showed potent antiviral activity against CMV (HEL cells, MIC_50_ 1.5 μM for both strains AD-169 and Davis) [16]. It was later shown that the active molecule is the (-) enantiomer (HEL cells, MIC_50_ 0.04–0.2 μM for strain AD-169 and MIC_50_ 0.1–0.3 μM for strain Davis), while the (+) enantiomer of 5′-noraristeromycin was three orders of magnitude weaker [17].

Based on previous work [9], we choose acetic acid as the linker connecting the HIV NNRTI and the NI of the heterodimers.

## 2. Results and Discussion 

### 2.1. Chemistry

**Scheme 1:**
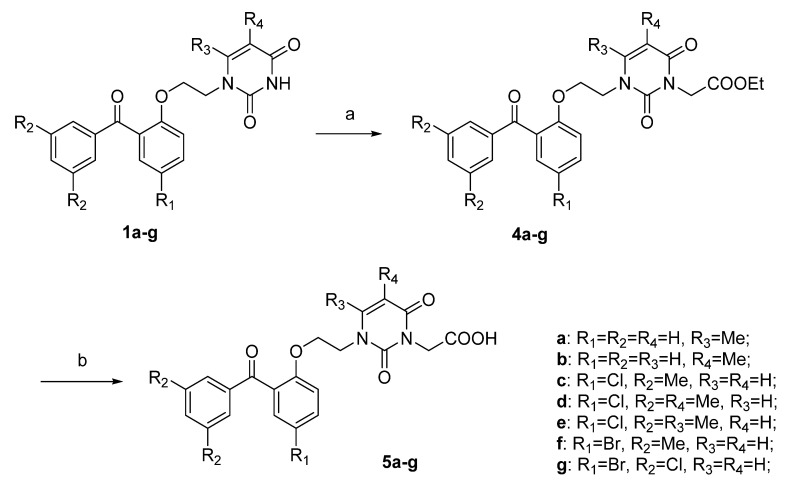
Synthesis of [2,6-dioxo-3-[(2-benzoylphenoxy)ethyl)]-3,6-dihydropyrimidin-1(2*H*)-yl]acetic acids **5a–5g**. *Reagents and conditions*: (a) BrCH_2_COOEt, K_2_CO_3_, DMF; (b) LiOH, H_2_O/EtOH.

The 1-[2-(2-benzoyl-4(*R*)-phenoxy)ethyl] derivatives of uracil **1a–1g** were treated with ethyl bromoacetate in a DMF solution in the presence of K_2_CO_3_ at room temperature to introduce the acetic acid moiety at the C^3^ position of the pyrimidine ring. The corresponding ethyl esters of [2,6-dioxo-3-[(2-benzoylphenoxy)ethyl)]-3,6-dihydropyrimidin-1(2*H*)-yl]acetic acids **4a–4g** were thus formed as crystals in 77–90% yield. One exception was the 6-methyluracil derivative **4e**, which was isolated as a viscous oil. Subsequent hydrolysis of esters **4a–4g** with LiOH in an aqueous-alcoholic medium at room temperature led to the formation of the desired [2,6-dioxo-3-[(2-benzoylphenoxy)ethyl)]-3,6-dihydropyrimidin-1(2*H*)-yl]acetic acids **5a–5g** in 80–94% yield (Scheme 1).

Nucleoside analogues ACV or 5′-noraristeromycin [13,17], were used as the second component of the heterodimers. ACV was kindly provided by the AZT Association. The synthesis, isolation and purification of the 5′-noraristeromycin precursor (−)-9-(4′-hydroxy-2′-cyclopenten-1′-yl)adenine were carried out by the methods previously described [15].

**Scheme 2:**
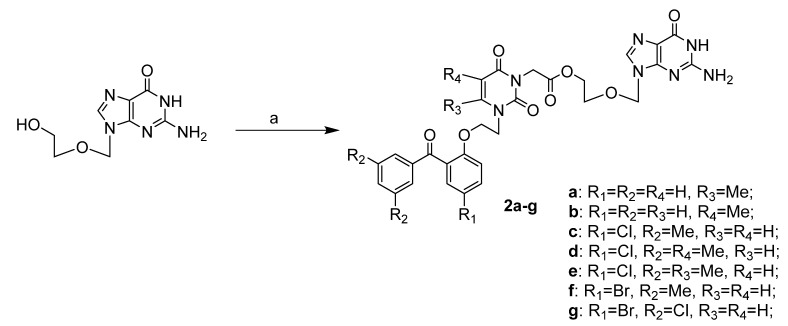
Synthesis of heterodimers **2a–2g**. *Reagents and conditions*: (a) **5a–5g**, EDC, DMAP, DMF.

The synthesis of compounds **2a–2g**, condensation of derivatives of [2,6-dioxo-3-[(2-benzoylphenoxy)ethyl)]-3,6-dihydropyrimidin-1(2H)-yl]acetic acids **5a–5g** with ACV was carried out in the presence of 1-ethyl-3-(3-dimethylaminopropyl)carbodiimide (EDC) and dimethyl-aminopyridine (DMAP) in dimethylformamide (DMF) (Scheme 2). The yields after isolation and purification ranged between 12.5–38.3%.

**Scheme 3:**
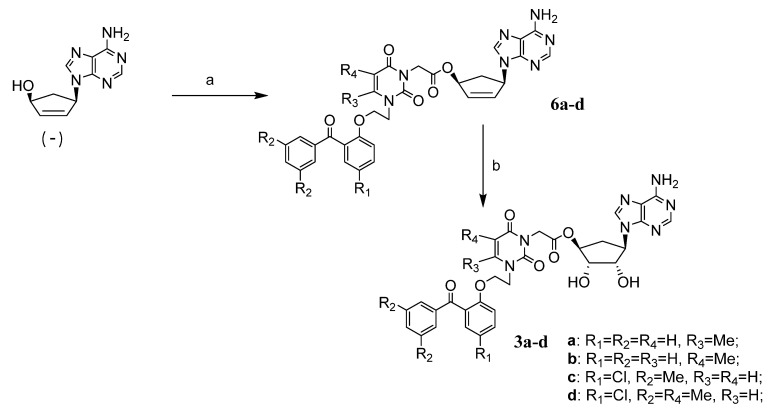
Synthesis of heterodimers **3a–3d**. *Reagents and conditions*: (a) **5a−5g**, EDCI, DMAP, DMF; (b) OsO_4_, NMMO, 1,4-dioxane; H_2_O (10:1).

Since the active compound is (−)-5′-noraristeromycin, for the synthesis of the second group of conjugates of [2,6-dioxo-3-[(2-benzoylphenoxy)ethyl)]-3,6-dihydropyrimidine-1(2*H*)-yl]acetic acids with 5′-noraristeromycin, we used (−)-9-(4′-hydroxy-2′-cyclopenten-1′-yl)adenine obtained in two stages according to the published procedure [15]. The (−)-isomer was condensed with the corresponding acids **5a–5d** as described above and the subsequent oxidation of the double bond of compounds **6a–6d** in the presence of osmium tetroxide gave targets **3a–3d** (Scheme 3). It should be noted that the product yields at the condensation stage in this case were significantly higher (52−76%) than in reactions with ACV (12.5–38.3%). The yields of the heterodimers based on (−)-9-(4′-hydroxy-2′-cyclopenten-1′-yl)adenine were 23.7−37.4%.

### 2.2. Hydrolysis of Compounds by Esterase from Porcine Liver

We assumed that the compounds **2a–2g** and **3a–3d** could be hydrolyzed enzymatically and generate biologically active components. We choose porcine esterase as one of the enzymes which could be involved in hydrolysis of synthesized compounds. The compounds **2d** and **3d** were taken as examples. T_1/2_ for compounds was about 9 h and hydrolysis was completed in 30 h. Reaction of **2d** with esterase gave two main products, identified by TLC as acyclovir and 1-[ω-(4-bromophenoxy)alkyl]uracil acetic acid (**5d**), respectively. Reaction of **3d** with esterase gave two main products identified by TLC as 5′-noraristeromycine and **5d**. Not even traces of the parental compound **1d** were found in both cases. Thymidine diacetate was used as a positive control; its hydrolysis was totally completed after 4 h under the same conditions.

### 2.3. Biological Activity

Using a prodrug is a very well-known and effective way to overcome potential shortcomings of a drug [18]. Usually a prodrug consists of the drug and an inactive “carrier” moiety, which, after hydrolysis in vivo, releases the drug inside the cell [18]. The concept of our heterodimer approach was to achieve dual action against HIV and herpes viruses, so by combining two drugs, it essentially gave a “mutual” prodrug, codrug where one drug serves as the carrier for the other and vice versa [19]. In our case, we combined two well-known nucleoside inhibitors of herpes family viruses ACV and 5′-noraristeromycin with nonnucleoside HIV RT inhibitors, substituted 2,6-dioxo-3-[(2-benzoylphenoxy)ethyl)]-3,6-dihydropyrimidines [10].

When creating dual-acting heterodimers however, it is very important to choose the right linker. The optimal linker should be non-toxic, able to sequentially cross-link with the given functional groups of both antiviral agents and give the heterodimer molecule the ability to release active components at the desired rate as a result of hydrolysis (chemical and/or enzymatic) in the body. The choice of a linker essentially determines the rate of release of the active components and, as a result, the level of activity of the individual compounds. We have previously shown that the acetic acid moiety was a successful linker in heterodimers consisting of 2′,3′-dideoxy-3′-azidothymidine and 1-[ω-(4-bromophenoxy)alkyl]uracil derivatives [9], thus we selected it for this investigation.

We obtained seven ACV-containing heterodimers **2a–2g** and four derivatives of 5′-noraristeromycin **3a–3d**. Compounds **2a–2g** and **3a** showed a lack of cytotoxicity on CEM (human lymphocytic) and HEL (human embryonic lung fibroblasts) cell cultures. After testing the heterodimers on a CEM cells infected with HIV-1 (strain IIIb) it was found that compounds **2a**, **2c–2g**, **3a–3d** had anti-HIV activity, but significantly lower than both the original NNRTIs **1a–1g** and antiviral drugs used as controls (Table 1). This is likely due to the ineffective release of the NNRTIs **1a−1g** in the conditions for the cell experiments.

ACV-containing heterodimers **2a–2g** were then tested in HEL cells infected with HSV-1 or HSV-2 (Table 2). None of the compounds showed significant inhibition of HSV-1 (data not shown), and while all derivatives were active against HSV-2, but less effective then ACV ranging from five (for **2e**) to 25 times worse (for **2a** and **2g**). Such a decrease in activity would seem to indicate a slow release of the ACV in cellular experiments.

Heterodimers **2a–2g** were also tested in HEL cells infected with VZV and CMV viruses (Table 3). The derivatives of ACV did not exhibit significant anti-CMV activity, but compounds **2b**, **2f** and **2g** showed moderate activity against VZV.

In addition, heterodimers containing 5′-noraristeromycin **3a–3d** did not show activity against HSV-1 and HSV-2 (data not shown). Slight inhibition of VZV was detected for compounds **3c** and **3d** (Table 3). CMV was moderately inhibited by compounds **3b–3d** (Table 3). It should be noted that for **3d,** the EC_50_ and CC_50_ values were almost the same. Derivatives of 5′-noraristeromycin, which is a known VV inhibitor, were additionally tested against this virus (Table 4). Compounds **3b** and **3d** showed anti-VV activity lower than the parent analogue by 14- and 8-fold less, respectively. Interestingly, compound **2f**, an ACV derivative, also showed weak activity against VV.

Thus, the lack of significant antiviral activity for the heterodimers suggests that the hydrolysis of the ester linkage and the release of nucleoside analogues under cellular experiment conditions goes too slowly to effectively deliver the drugs in a timely fashion. A significant drop in anti-HIV activity is most likely due to the lack of hydrolysis of the acetic acid residue from uracil fragment. Instead of compounds **1a–1g**, low active acetic acids **5a–5g** were obtained. Thus, the use of acetic acid as the linker, which proved successful in previously studied and structurally similar heterodimers [9], did not give the desired results in this current study. Optimization of the linker to achieve more efficient hydrolysis for obtaining a higher inhibitory effect is currently underway and the results will be published as they become available.

## 3. Materials and Methods

### 3.1. General Information

All reagents (highest grade available) were obtained from Sigma (St. Louis, MO, USA) and Acros Organics (Geelcity, Belgium) and used without further purification unless otherwise noted. Anhydrous DMF and isopropanol were purchased from Sigma-Aldrich (Madison, WI, USA). Anhydrous 1,2-dichloroethane, and ethyl acetate were obtained by distillation over P_2_O_5_.

Column chromatography was performed on Silica Gel 60 0.040−0.063 mm (Merck, Darmstadt, Germany), and systems for elution are indicated in the text. Thin layer chromatography (TLC) was performed on TLC Silica gel 60 F254 plates (Merck). Preparative layer chromatography (PLC) was performed on PLC Silica gel 60 F254 plates (Merck). Flash chromatography was performed on Kieselgel 60−200 micron, 60A (Acros Organics). ^1^H- and ^13^C-nuclear magnetic resonance (NMR) spectra were registered on a Bruker Avance 400 (400 MHz for ^1^H and 100 MHz for ^13^C) spectrometer and Bruker Avance 300 (300 MHz for ^1^H and 100 MHz for ^13^C) spectrometer (Bruker, Newark, Germany) using tetramethylsilane (TMS) in CDCl_3_, CD_3_OD, CDCl_3_/CD_3_OD mixture, or DMSO-d_6_ as internal standard. Chemical shifts are given in ppm, and the letter “*J*” indicates normal 3*J*HH couplings and all *J* values are given in Hz. High-resolution mass spectra (HRMS) were registered on a Bruker Daltonics micrOTOF-Q II instrument using electrospray ionization (ESI). The measurements were acquired in a negative ion mode with the following parameters: interface capillary voltage-3700 V; mass range from *m/z* 50 to 3000; external calibration (Electrospray Calibrant Solution, Fluka, Newark, NH, USA); nebulizer pressure 0.3 Bar; flow rate- 3 L/min; dry gas nitrogen (4.0 L/min); interface temperature was set at 180 or 190 °C. A syringe injection was used. Melting points were determined using a Mel-Temp 3.0 apparatus (Laboratory Devices Inc., Auburn, CA, USA).

### 3.2. Chemistry

Starting benzophenone analogues of uracil and thymine **1b, 1c, 1e** and **1f** were synthesized according to the published protocol [10], and benzophenone derivatives of 6-methyluracil as described earlier [20].

#### 3.2.1. General Method for the Synthesis of Ethyl Ester of [2,6-dioxo-3-[(2-benzoylphenoxy)ethyl]-3,6-dihydropyrimidine-1(2*H*)-yl]carboxylic acids **4a–4g**

A mixture of the 1-[2-(2-benzoylphenoxy)ethyl]uracil derivative **1** (1.69 mmol) and K_2_CO_3_ (0.3 g; 2.17 mmol) was stirred in DMF (10 mL) at 80 °C during 1 h. and then cooled to room temperature. Then ethyl bromoacetate was added (0.2 mL; 1.80 mmol). The resulting mixture was stirred for 20 h at room temperature. Then the reaction mixture was evaporated in vacuo, the residue was treated with 50 mL of cold water and extracted with 1,2-dichloroethane (5 × 20 mL). The extract was evaporated under reduced pressure and the residue was purified by flash chromatography on silica gel eluting with ethyl acetate. The fractions containing the product were crystallized from ethyl acetate-hexane. The yield of target esters **4a–4g** was in the range of 77–89%. Compound **4e** was obtained as a viscous oil with a yield of 74%.

*Ethyl ester of [2,6-dioxo-3-[(2-benzoylphenoxy)ethyl]-6-methyl-3,6-dihydropyrimidine-1(2H)-yl]carboxylic acid* (**4a**). Purified by crystallization from an ethyl acetate-hexane (2:1) mixture in 90% yield. Mp 150–151 °C, R*_f_* 0.68 (ethyl acetate:1,2-dichloroethane, 1:1). ^1^H-NMR (DMSO-d_6_): 1.15 (3H, t, *J* = 7.0 Hz, CH_3_), 1.89 (3H, s, CH_3_), 3.94 (2H, t, *J* = 5.0 Hz, CH_2_-N), 4.09 (2H, qd, *J* = 7.1 Hz, CH_2_), 4.19 (2H, t, *J* = 5.0 Hz, CH_2_-O), 4.45 (2H, s, CH_2_CO), 5.29 (1H, s, H5Ura), 7.10 (1H, s, *J* = 7.5 Hz, HPh_2_CO), 7.20 (1H, t, *J* = 8.4 Hz, HPh2CO), 7.28 (1H, dd, *J* = 7.4 and 1.7 Hz, HPh_2_CO), 7.34–7.47 (2H, m, HPh_2_CO), 7.51 (1H, dt, *J* = 8.0 and 1.7 Hz, HPh_2_CO), 7.58 (1H, t, *J* = 7.3 Hz, HPh_2_CO), 7.64–7.66 (2H, m, HPh_2_CO). ^13^C-NMR (DMSO-d_6_): 14.0, 19.4, 41.56, 44.2, 60.9, 65.7, 100.2, 112.7, 121.3, 128.5, 128.6, 128.8, 129.2, 131.2, 133.6, 136.5, 151.2, 153.4, 155.2, 160.5, 167.8, 195.5 (see Supplementary Materials).

*Ethyl ester of [2,6-dioxo-3-[(2-benzoylphenoxy)ethyl]-5-methyl-3,6-dihydropyrimidine-1(2H)-yl]carboxylic acid* (**4b**)**.** Purified by crystallization from an ethyl acetate-hexane mixture (2:1) in 77% yield. Mp 118–119 °C, R*_f_* 0.57 (ethyl acetate:1,2-dichloroethane, 1:1).^1^H-NMR (DMSO-d_6_): 1.16 (3H, t, *J* = 7.1 Hz, CH_3_), 1.63 (3H, s, CH_3_), 3.86 (2H, t, *J* = 5 Hz, CH_2_), 4.10 (2H, t, *J* = 7.1 Hz, CH_2_), 4.19 (2H, qd, *J* = 5.0 Hz, CH_2_), 4.46 (2H, s, CH_2_CO), 6.98 (1H, d, *J* = 1.1 Hz, H6Thy), 7.10 (1H, t, *J* = 7.5 Hz, HPh_2_CO), 7.21 (1H, t, *J* = 8.4 Hz, HPh_2_CO), 7.30 (1H, dd, *J* = 7.5 and 1.7 Hz, HPh_2_CO), 7.45–7.49 (2H, m, HPh_2_CO), 7.52 (1H, dt, *J* = 8.0 and 1.7 Hz, HPh_2_CO), 7.59 (1H, t, *J* = 7.4 Hz, HPh_2_CO), 7.65–7.67 (2H, m, HPh_2_CO). ^13^C-NMR (DMSO-d_6_): 12.4, 14.0, 41.8, 48.0, 60.9, 65.6, 107.1, 112.9, 121.2, 128.5, 128.9, 129.1, 132.0, 133.4, 136.8, 140.8, 150.4, 155.3, 162.4, 167.8, 195.3.

*Ethyl ester of [2,6-dioxo-3-[(2-[2-(3,5-dimethylbenzoyl)-4-chlorophenophenoxy]ethyl]-3,6-dihydropyrimidine-1(2H)-yl]carboxylic acid* (**4c**). Purified by crystallization from an ethyl acetate-hexane mixture (2:1) in 84% yield. Mp 152–153 °C, R_*f*_ 0.67 (ethyl acetate:1,2-dichloroethane, 1:1).^1^H-NMR (DMSO-d_6_): 1.16 (3H, t, *J* = 7.0 Hz, CH_3_), 2.28 (6H, t, CH_3_), 3.72 (2H, t, *J* = 4.9 Hz, N-CH_2_), 4.09 (2H, t, *J* = 7.0 Hz, O-CH_2_), 4.18 (2H, qd, *J* = 5.1 Hz, CH_2_), 4.43 (2H, s, CH_2_CO), 5.32 (1H, d, *J* = 7.9 Hz, H5Ura), 6.94 (1H, d, *J* = 8.0 Hz, H-3”), 7.22–7.34 (4H, m, H-5”, H-2‘, H-4‘, H-6′), 7.55 (1H, dd, *J* = 8.9 and 2.7 Hz, H-6”). ^13^C-NMR (DMSO-d_6_): 14.0, 20.7, 41.5, 48.1, 61.0, 66.1, 99.4, 114.9, 125.1, 127.0, 128.1, 130.6, 131.3, 125.2, 136.5, 138.0, 144.4, 150.6, 154.0, 161.5, 167.6, 193.9.

*Ethyl ester of [2,6-dioxo-3-[(2-[2-(3,5-dimethylbenzoyl)-4-chlorophenophenoxy]ethyl]-5-methyl-3,6-dihydropyrimidine-1(2H)-yl]carboxylic acid* (**4d**). Purified by crystallization from an ethyl acetate-hexane mixture (2:1) in 87% yield. Mp 153-154 °C, R_*f*_ 0.73 (ethyl acetate:1,2-dichloroethane, 1:1).^1^H- NMR (DMSO-d_6_): 1.16 (3H, t, *J* = 5.0 Hz, CH_3_), 2.27 (3H, s, CH_3_), 2.29 (6H, s, CH_3_), 3.86 (2H, t, *J* = 5.0 Hz, N–CH_2_), 4.09 (2H, qd, *J* = 7.2 Hz, CH_2_), 4.20 (2H, t, *J* = 5.0 Hz, O–CH_2_), 4.44 (2H, s, CH_2_CO), 6.98 (1H, s, H-3”), 7.24–7.31 (5H, m, H-5”, H-2′, H-4′, H-6′, H6Ura), 7.54 (1H, dd, *J* = 8.9 и 2.6 Hz, H-6”). ^13^C-NMR (DMSO-d_6_): 12.2, 13.9, 20.6, 41.6, 47.9, 60.9, 66.1, 107.0, 115.1, 125.0, 126.9, 128.0, 130.6, 131.2, 135.1, 136.2, 137.9, 140.7, 150.3, 154.0, 162.2, 167.6, 193.7.

*Ethyl ester of [2,6-dioxo-3-[(2-[2-(3,5-dimethylbenzoyl)-4-chlorophenophenoxy]ethyl]-6-methyl-3,6-dihydropyrimidine-1(2H)-yl]carboxylic acid* (**4e**). Was obtain as an oil in 74% yield and used in the subsequent hydrolysis without further purification. R_*f*_ 0.64 (ethyl acetate:1,2-dichloroethan, 1:1).

*Ethyl ester of [2,6-dioxo-3-[(2-[2-(3,5-dimethylbenzoyl)-4-bromophenophenoxy]ethyl]-3,6-dihydropyrimidine-1(2H)-yl]carboxylic acid* (**4f**). Purified by crystallization from an ethyl acetate-hexane mixture (2:1) in 89% yield. Mp 152–153 °C, R_*f*_ 0.66 (ethyl acetate:1,2-dichloroethanw, 1:1).^1^H-NMR (DMSO-d_6_): 1.16 (3H, t, *J* = 7.1 Hz, CH_3_), 2.23 (6H, s, CH_3_), 3.87 (2H, t, *J* = 4.8 Hz, N-CH_2_), 4.09 (2H, qd, *J* = 7.1 Hz, CH_2_), 4.20 (2H, t, *J* = 5.0 Hz, O–CH_2_), 4.42 (2H, s, CH_2_CO), 5.31 (1H, d, *J* = 8.0 Hz, H5Ura), 6.93 (1H, d, *J* = 7.9 Hz, H-3”), 7.18 (1H, d, *J* = 8.0 Hz, H6Ura), 7.26 (1H, s, H-4′), 7.27 (2H, s, H-2′, H-6′), 7.44 (1H, d, *J* = 2.5 Hz, H-3′), 7.67 (1H, dd, *J* = 8.9 and 2.6 Hz, H-6”); ^13^C-NMR (DMSO-d_6_): 14.0, 20.7, 41.5, 48.1, 61.0, 66.0, 99.4, 112.7, 115.4, 127.0, 130.9, 131.0, 134.2, 135.2, 136.5, 138.0, 144.4, 150.6, 154.5, 161.5, 167.6, 193.8.

*Ethyl ester of [2,6-dioxo-3-[(2-[2-(3,5-dichlorobenzoyl)-4-bromophenophenoxy]ethyl]-3,6-dihydropyrimidine-1(2H)-yl]carboxylic acid* (**4g**). Purified by crystallization from an ethyl acetate-hexane mixture (2:1) in 86% yield. Mp 162.5–165.5 °C, R_*f*_ 0.70 (ethyl acetate:1,2-dichloroethane, 1:1).^1^H-NMR (DMSO-d_6_): 1.16 (3H, t, *J* = 7.1 Hz, CH_3_), 3.92 (2H, t, *J* = 4.9 Hz, N-CH_2_), 4.10 (2H, qd, *J* = 7.2 Hz, CH_2_), 4.22 (2H, t, *J* = 5.0 Hz, O–CH_2_), 4.46 (2H, s, CH_2_CO), 5.41 (1H, d, *J* = 7.9 Hz, H5Ura), 7.18 (1H, d, *J* = 7.9 Hz, H6Ura), 7.21 (1H, d, *J* = 9.0 Hz, H-5”), 7.55 (1H, d, *J* = 2.4 Hz, H-4′), 7.57 (2H, d, *J* = 1.9 Hz, H-2′, H-6′), 7.73 (1H, dd, *J* = 8.9 and 2.6 Hz, H-6”), 7.85 (1H, t, *J* = 1.7 Hz, H-3”). ^13^C-NMR (DMSO-d_6_): 13.9, 41.4, 47.9, 60.9, 65.9, 99.3, 112.7, 115.4, 127.4, 128.9, 131.4, 132.8, 134.6, 135.1, 139.3, 144.4, 150.5, 154.6, 161.3, 167.5, 191.2.

#### 3.2.2. General Method for the Synthesis of [2,6-dioxo-3-[(2-benzoylphenoxy)ethyl]-3,6-dihydropyrimidine-1(2*H*)-yl]carboxylic acids **5a–5g**

The corresponding ethyl ester of [2,6-dioxo-3-[(2-benzoylphenoxy)ethyl)]-3,6-dihydro-pyrimidin-1(*2H*)-yl]acetic acid **4a–4g** (1.75 mmol) was dissolved in ethanol (50 mL) then LiOH (0.25 g; 10.44 mmol) and water (30 mL) were added. The resulting mixture was stirred at room temperature for 24 h. Ethanol was evaporated under reduced pressure, 50 mL of water was added to the residue, acidified with 6% aqueous hydrochloric acid, and refrigerated overnight. The precipitate was filtered off, washed on the filter with a large amount of water and dried. After crystallization from an ethyl acetate-hexane mixture, the yield of the desired acids **5a–5g** was in the range of 80–94%.

*[2,6-Dioxo-3-[(2-benzoylphenoxy)ethyl]-6-methyl-3,6-dihydropyrimidine-1(2H)-yl]carboxylic* acid (**5a**). Purified by crystallization from an ethyl acetate-hexane mixture (3:1) in 90% yield. Mp 194.5–196 °C, R*_f_* 0.63 (ethyl acetate: isopropanol: 40% aq. NH_4_OH, 6:9:5).^1^H-NMR (DMSO-d_6_): 1.89 (3H, s, CH_3_), 3.93 (2H, t, *J* = 5.0 Hz, CH_2_), 4.20 (2H, t, *J* = 5.1 Hz, CH_2_), 4.36 (2H, s, CH_2_CO), 5.30 (1H, d, *J* = 0.7 Hz, H5Ura), 7.10 (9H, dt, *J* = 7.5 and 0.8 Hz, HPh_2_CO), 7.20 (1H, d, *J* = 8.4 Hz, HPh_2_CO), 7.28 (1H, dd, *J* = 7.4 and 1.6 Hz, HPh_2_CO), 7.43–7.47 (2H, m, HPh_2_CO), 7.49–7.54 (1H, m, HPh_2_CO), 7.58 (1H, dt, *J* = 7.3 and 1.3 Hz, HPh_2_CO), 7.64–7.66 (1H, m, HPh_2_CO), 12.86 (1H, bs, COOH). ^13^C-NMR (DMSO-d_6_): 19.3, 41.5, 44.1, 65.7, 100.2, 112.8, 121.2, 128.4, 128.6, 128.8, 129.1, 131.7, 133.5, 136.5, 151.2, 155.2, 157.1, 160.5, 169.0, 195.5.

*[2,6-Dioxo-3-[(2-benzoylphenoxy)ethyl]-6-methyl-3,6-dihydropyrimidine-1(2H)-yl]carboxylic acid* (**5b**). Purified by crystallization from an ethyl acetate-hexane mixture (3:1) in 87% yield. Mp 195.5–196.5 °C, R*_f_* 0.6 (ethyl acetate: isopropanol: 40% aq. NH_4_OH, 6:9:5).^1^H-NMR (DMSO-d_6_): 1.63 (3H, d, *J* = 0.8 Hz, CH_3_), 3.85 (2H, t, *J* = 5.0 Hz, CH_2_-N), 4.19 (2H, t, *J* = 5.0 Hz, CH_2_-O), 4.37 (2H, s, CH_2_CO), 6.97 (1H, d, *J* = 1.1 Hz, HPh_2_CO), 7.10 (1H, t, *J* = 7.4 Hz, HPh_2_CO), 7.21 (1H, d, *J* = 8.4 Hz, HPh_2_CO), 7.30 (1H, dd, *J* = 7.5 and 1.7 Hz, HPh_2_CO), 7.47 (2H, t, *J* = 7.5 Hz, HPh_2_CO), 7.52 (1H, dt, *J* = 8.0 and 1.7 Hz, HPh_2_CO), 7.60 (1H, t, *J* = 7.4 Hz, HPh_2_CO), 7.65–7.67 (1H, t, HPh2CO, H6Ura), 12.86 (1H, bs, COOH). ^13^C-NMR (DMSO-d_6_): 12.3, 41.7, 47.9, 65.6, 107.0, 112.9, 121.1, 128.4, 128.8, 129.0, 132.0, 133.3, 136.7, 140.5, 150.4, 155.3, 162.3, 169.0, 195.2.

*[2,6-Dioxo-3-[(2-[2-(3,5-dimethylbenzoyl)-4-chlorophenophenoxy]ethyl]-3,6-dihydropyrimidine-1(2H)-yl]carboxylic* acid (**5c**). Purified by crystallization from an ethyl acetate-hexane (3:1) mixture in 85% yield. Mp 215–216 °C, R*_f_* 0.57 (ethyl acetate: isopropanol: 40% aq. NH_4_OH, 6:9:5).^1^H-NMR (DMSO-d_6_): 2.28 (6H, s, CH_3_), 3.87 (2H, t, *J* = 4.9 Hz, N-CH_2_), 4.18 (2H, t, *J* = 5.1 Hz, O-CH_2_), 4.34 (2H, s, CH_2_CO), 5.32 (1H, d, *J* = 7.9 Hz, H5Ura), 6.94 (1H, d, *J* = 7.9 Hz, H-5”), 7.22–7.27 (4H, m, H-2′, H-4′, H-6′, H6Ura), 7.34 (1H, d, *J* = 2.6 Hz, H-3”), 7.55 (1H, dd, *J* = 8.9 and 2.8 Hz, H-6”), 12.88 (1H, bs, COOH). ^13^C-NMR (DMSO-d_6_): 20.6, 41.3, 48.0, 66.0, 99.3, 114.9, 125.0, 126.9, 128.0, 130.5, 131.2, 135.1, 136.4, 137.9, 144.2, 150.5, 153.9, 161.5, 168.9, 193.8.

*[2,6-Dioxo-3-[(2-[2-(3,5-dimethylbenzoyl)-4-chlorophenophenoxy]ethyl]-5-methyl-3,6-dihydropyrimidine-1(2H)-yl]carboxylic* acid (**5d**). Purified by crystallization from an ethyl acetate-hexane mixture (3:1) in 94% yield. Mp 238–239 °C, R*_f_* 0.6 (ethyl acetate: isopropanol: 40% aq. NH_4_OH, 6:9:5).^1^H-NMR (DMSO-d_6_): 1.62 (3H, s, CH_3_), 2.28 (6H, s, CH_3_), 3.86 (2H, t, *J* = 5.0 Hz, N–CH_2_), 4.20 (2H, t, *J* = 5.0 Hz, O–CH_2_), 4.35 (2H, s, CH_2_CO), 6.97 (1H, d, *J* = 7.9 Hz, H-5”), 7.22–7.27 (4H, m, H-2′, H-4′, H-6′, H6Ura), 7.31 (1H, d, *J* = 2.7 Hz, H-3”), 7.55 (1H, dd, *J* = 8.9 and 2.7 Hz, H-6), 12.87 (1H, bs, COOH). ^13^C-NMR (DMSO-d_6_): 12.2, 20.6, 41.6, 47.8, 66.1, 107.0, 115.1, 125.0, 126.9, 127.9, 130.5, 131.1, 135.1, 136.2, 137.8, 140.5, 150.4, 153.9, 162.2, 169.0, 193.7. 

*[2,6-Dioxo-3-[(2-[2-(3,5-dimethylbenzoyl)-4-chlorophenophenoxy]ethyl]-6-methyl-3,6-dihydropyrimidine-1(2H)-yl]carboxylic acid* (**5e**). Purified by crystallization from an ethyl acetate-hexane mixture (3:1) in 80% yield. Mp 213–214.5 °C, R*_f_* 0.56 (ethyl acetate: isopropanol: 40% aq. NH_4_OH, 6:9:5).^1^H-NMR (DMSO-d_6_): 1.87 (3H, s, CH_3_), 2.26 (6H, c, CH_3_), 3.94 (2H, t, *J* = 4.9 Hz, N–CH_2_), 4.20 (2H, t, *J* = 5.0 Hz, O–CH_2_), 4.36 (2H, s, CH_2_CO), 5.25 (1H, s, H5Ura), 7.22 (1H, s, H-4′), 7.25 (3H, s, H-2′, H-6′), 7.32 (1H, d, *J* = 2.6 Hz, H-3”), 7.54 (1H, dd, *J* = 8.9 and 2.7 Hz, H-6”), 12.87 (1H, bs, COOH). ^13^C-NMR (DMSO-d_6_): 19.3, 20.6, 41.4, 44.0, 66.2, 100.0, 114.8, 125.1, 126.9, 127.7, 130.7, 131.0, 135.3, 136.0, 138.0, 151.2, 153.0, 153.9, 160.4, 168.9, 194.0.

*[2,6-Dioxo-3-[(2-[2-(3,5-dimethylbenzoyl)-4-bromophenophenoxy]ethyl]-3,6-dihydropyrimidine-1(2H)-yl]carboxylic acid* (**5f**). Purified by crystallization from an ethyl acetate-hexane mixture (3:1) in 81% yield. Mp 224.5–225.5 °C, R*_f_* 0.47 (ethyl acetate: isopropanol: 40% aq. NH_4_OH, 6:9:5).^1^H-NMR (DMSO-d_6_): 2.28 (6H, s, CH_3_), 3.87 (2H, t, *J* = 5.0 Hz, N-CH_2_), 4.18 (2H, t, *J* = 5.0 Hz, O-CH_2_), 4.34 (2H, s, CH_2_CO), 5.31 (1H, d, *J* = 7.8 Hz, H5Ura), 6.93 (1H, d, *J* = 8.0 Hz, H-5”), 7.18 (1H, d, *J* = 9.0 Hz, H6Ura), 7.27 (3H, м, H-2′, H-4′, H-6′), 7.45 (1H, d, *J* = 2.6 Hz, H-3”), 7.67 (1H, dd, *J* = 8.9 and 2.6 Hz, H-6”), 12.89 (1H, bs, COOH). ^13^C-NMR (DMSO-d_6_): 20.6, 41.3, 48.0, 66.0, 99.3, 112.6, 115.3, 126.9, 130.8, 130.9, 134.1, 135.1, 136.4, 137.9, 144.2, 150.5, 154.4, 161.5, 168.9, 193.7.

*[2,6-Dioxo-3-[(2-[2-(3,5-dichlorobenzoyl)-4-bromophenophenoxy]ethyl]-3,6-dihydropyrimidine-1(2H)-yl]carboxylic acid* (**5g**). Purified by crystallization from an ethyl acetate-hexane mixture (3:1) in 86% yield. Mp 212–213 °C, R*_f_* 0.51 (ethyl acetate: isopropanol: 40% aq. NH_4_OH, 6:9:5).^1^H-NMR (DMSO-d_6_): 3.92 (2H, t, *J* = 5.0 Hz, N-CH_2_), 4.18 (2H, t, *J* = 5.0 Hz, O-CH_2_), 4.38 (2H, s, CH_2_CO), 5.40 (1H, d, *J* = 7.8 Hz, H5Ura), 7.16 (1H, d, *J* = 7.8 Hz, H-5”), 7.21 (1H, d, *J* = 8.9 Hz, H6Ura), 7.55 (1H, d, *J* = 2.6 Hz, H-4′), 7.57 (2H, d, *J* = 2.0 Hz, H-2′, H-6′), 7.73 (1H, dd, *J* = 8.9 and 2.6 Hz, H-6”), 7.85 (1H, t, *J* = 2.0 Hz, H-3”), 12.89 (1H, bs, COOH). ^13^C-NMR (DMSO-d_6_): 41.4, 47.9, 65.9, 99.3, 112.7, 115.4, 127.4, 128.9, 131.4, 132.8, 134.6, 135.1, 139.3, 144.2, 150.5, 154.6, 161.4, 168.8, 191.2.

#### 3.2.3. General Method for the Synthesis of ACV Derivatives

A mixture of the corresponding [2,6-dioxo-3-[(2-benzoylphenoxy)ethyl)]-3,6-dihydropyrimidin-1(2H)-yl]acetic acid (0.5 mmol) and ACV (0.5 mmol) was twice evaporated in DMF, then dissolved in DMF (5 mL) and 1-ethyl-3-(3-dimethylaminopropyl)carbodiimide (1.2 eq) and dimethyl-aminopyridine (0.5 eq) were added. The reaction mixture was allowed to mix for 16 h at room temperature. The progress of the reaction was monitored by TLC. The solvent was then evaporated and the residue was purified by column chromatography on silica gel eluting with a 9:1 chloroform-methanol mixture. The yield of the target products was 26–39%.

*[2,6-Dioxo-3-[(2-benzoylphenoxy)ethyl]-6-methyl-3,6-dihydropyrimidine-1(2H)-yl]acetate of ACV* (**2a**). Purified on a silica gel column using chloroform:methanol (9:1) as eluent in 33% yield. R*_f_* 0.57 (chloroform:methanol, 95:5). ^1^H-NMR (CD_3_OD:CDCl_3_): 1.97 (3H, s, CH_3_), 3.88-3.91 (2H, m, CH_2_O), 4.04–4.07 (2H, m, CH_2_N), 4.24–4.37 (4H, m, 2 × CH_2_CH_2_C=O), 4.58 (2H, s, NCH_2_C=O), 5.29 (1H, d, H5Ura), 5.64 (2H, s, OCH_2_N), 7.06–7.12 (2H, m, Ph), 7.24–7.28 (1H, d, Ph), 7.40–7.52 (3H, m, Ph), 7.54–7.60 (1H, m, Ph), 7.73–7.76 (2H, d, Ph), 9.02 (1H, s, H8). ^13^C-NMR (DMSO-d_6_): 19.9, 41.9, 44.6, 64.3, 66.1, 66.7, 72.3, 100.6, 113.25, 117.0, 121.7, 129.0, 129.1, 129.2, 129.7, 132.3, 134.1, 136.9, 138.0, 151.6, 151.9, 153.9, 154.6, 155.6, 155.6, 157.2, 161.0, 168.3, 196. HRMS: *m/z* [M + H]^+^ calcd for C_30_H_29_N_7_O_8_: 616.2150, found: 616.2136.

*[2,6-Dioxo-3-[(2-benzoylphenoxy)ethyl]-5-methyl-3,6-dihydropyrimidine-1(2H)-yl]acetate of* ACV (**2b**). Purified on a silica gel column using chloroform:methanol (9:1) as eluent in 23% yield. R*_f_* 0.62 (chloroform:methanol, 95:5). ^1^H-NMR (CD_3_OD:CDCl_3_): 1.72 (3H, s, CH_3_), 3.70–3.78 (2H, m, CH_2_O), 3.91–3.94 (2H, m, CH_2_N), 4.20–4.30 (4H, m, 2 × CH_2_CH_2_C=O), 4.61 (2H, s, NCH_2_C=O), 5.49 (2H, s, OCH_2_N), 6.80–6.81 (1H, s, H6Ura), 7.03-7.10 (2H, m, Ph), 7.30–7.33 (1H, d, *J* = 12Hz, Ph), 7.44–7.50 (3H, m, Ph), 7.57–7.60 (1H, m, Ph), 7.76-7.79 (2H, d, Ph), 8.17 (1H, s, H8). ^13^C-NMR (DMSO-d_6_): 19.9, 41.9, 44.6, 64.3, 66.1, 66.8, 72.3, 100.6, 113.3, 117.0, 121.7, 129.0, 129.1, 129.2, 129.7, 132.3, 134.1, 136.9, 138.0, 151.6, 151.9, 153.9, 154.5, 155.6, 157.2, 160.9, 168.3, 196.0 HRMS: *m/z* [M + H]^+^ calcd for C_30_H_29_N_7_O_8_: 616.2150, found: 616.2128.

*[2,6-Dioxo-3-[(2-[2-(3,5-dimethylbenzoyl)-4-chlorophenophenoxy]ethyl]-3,6-dihydropyrimidine-1(2H)-yl]-acetate of ACV* (**2c**). Purified on a silica gel column using chloroform:methanol (9:1) as eluent in 19% yield. R*_f_* 0.65 (chloroform:methanol, 95:5). ^1^H-NMR (CD_3_OD): 2.29 (6H, s, 2 × CH_3_), 3.77-3.78 (2H, m, CH_2_O), 3.90–3.91 (2H, m, CH_2_N), 4.16–4.22 (4H, m, 2 × CH_2_CH_2_C=O), 4.50 (2H, s, NCH_2_C=O), 5.26–5.28 (1H, d, *J* = 8Hz, H5Ura), 5.47 (2H, s, OCH_2_N), 6.79–6.82 (1H, d, *J* = 12Hz, H6Ura), 7.07–7.10 (1H, d, *J* = 12Hz, Ph), 7.21–7.24 (2H, m, Ph), 7.30 (2H, m, Ph), 7.45–7.42 (1H, m, Ph), 8.23 (1H, s, H8). ^13^C-NMR (CD_3_OD): 19.9, 31.6, 41.4, 60.9, 63.9, 66.2, 67.3, 70.2, 72.3, 72.9, 99.5, 114.3, 126.2, 127.2, 128.3, 130.8, 131.8, 135.3, 136.5, 144.7, 151.0, 154.3, 154.9, 156.7, 162.9, 168.0, 195.1. HRMS: *m/z* [M − H]^−^ calcd for C_31_H_30_ClN_7_O_8_: 662.1761, found: 662.1763.

*[2,6-Dioxo-3-[(2-[2-(3,5-dimethylbenzoyl)-4-chlorophenophenoxy]ethyl]-5-methyl-3,6-dihydropyrimidine-1(2H)-yl]acetate of ACV* (**2d**). Purified on a silica gel column using chloroform:methanol (9:1) as eluent in 38% yield. R*_f_* 0.59 (chloroform:methanol, 95:5). ^1^H-NMR (DMSO-d_6_): 1.88 (3H, s, CH3), 2.26 (6H, s, 2 × CH3), 3.66–3.69 (2H, m, CH_2_O), 3.94–3.96 (2H, m, CH_2_N), 4.15–4.22 (4H, m, 2 × CH_2_CH_2_C=O), 4.25 (2H, s, NCH_2_C=O), 5.36 (2H, s, OCH_2_N), 6.58 (2H, s, NH2), 7.03 (1H, d, H6 Ura), 7.20–7.37 (5H, m, Ph), 7.53–7.57 (1H, m, Ph), 7.85 (1H, s, H8), 10.70 (1H, s, NH). ^13^C-NMR (DMSO-d_6_): 19.9, 21.1, 41.8, 44.6, 64.3, 66.6, 66.8, 70.3, 72.3, 100.5, 115.3, 116.8, 125.6, 127.5, 128.2, 131.2, 135.9, 136.5, 138.1, 138.6, 151.6, 151.9, 153.9, 154.4, 157.2, 160.9, 168.2, 194.5. HRMS: *m/z* [M + H]^+^ calcd for C_32_H_32_ClN_7_O_8_: 678.2074, found: 678.2073.

*[2,6-Dioxo-3-[(2-[2-(3,5-dimethylbenzoyl)-4-chlorophenophenoxy]ethyl]-6-methyl-3,6-dihydropyrimidine-1(2H)-yl]acetate of* ACV (**2e**). Purified on a silica gel column using chloroform:methanol (9:1) as eluent in 14% yield. R*_f_* 0.6 (chloroform:methanol, 95:5). ^1^H-NMR (DMSO-d_6_): 1.83 (3H, s, CH_3_), 2.21 (6H, s, 2 × CH_3_), 3.66–3.67 (2H, m, CH_2_O), 3.88–3.91 (2H, m, CH_2_N), 4.12–4.17 (4H, m, 2 × CH_2_CH_2_C=O), 4.40 (2H, m, NCH_2_C=O), 5.22 (1H, d, H5Ura), 5.36 (2H, s, OCH_2_N), 6.75 (2H, s, NH_2_), 7.19–7.22 (5H, m, Ph), 7.48–7.52 (1H, m, Ph), 8.17 (1H, s, H8), 10.94 (1H, s, NH). ^13^C-NMR (DMSO-d_6_): 12.7, 21.2, 42.0, 48.3, 64.3, 66.6, 66.8, 70.3, 72.4, 107.5, 115.6, 125.6, 127.4, 128.5, 131.1, 131.7, 135.7, 136.7, 138.4, 141.3, 150.8, 154.5, 162.7, 168.2, 194.2. HRMS: *m/z* [M + H]^+^ calcd for C_32_H_32_ClN_7_O_8_: 678.2074, found: 678.2076.

*[2,6-Dioxo-3-[(2-[2-(3,5-dimethylbenzoyl)-4-bromophenophenoxy]ethyl]-3,6-dihydropyrimidine-1(2H)-yl]acetate of ACV* (**2f**). Purified on a silica gel column using chloroform:methanol (9:1) as eluent in 22% yield. R*_f_* 0.64 (chloroform:methanol, 95:5). ^1^H-NMR (CD_3_OD): 2.34 (6H, s, 2 × CH_3_), 3.76–3.79 (2H, m, CH_2_O), 3.95–3.97 (2H, m, CH_2_N), 4.23–4.26 (4H, m, 2 × CH_2_CH_2_C=O), 4.55 (2H, s, NCH_2_C=O), 5.30-5.33 (1H, d, *J* = 12 Hz, H5Ura), 5.45 (2H, s, OCH_2_N), 6.85-6.88 (1H, d, *J* = 12 Hz, H-6), 7.07–7.10 (1H, d, *J* = 12Hz, Ph), 7.36 (1H, m, Ph), 7.39–7.40 (3H, m, Ph), 7.61-7.65 (1H, m, Ph), 7.82 (1H, s, H8). ^13^C-NMR (DMSO-d_6_): 21.1, 41.8, 48.6, 64.4, 66.5, 66.8, 70,3, 72.3, 99.8, 113.2, 115.9, 117.0, 127.4, 131.3, 131.4, 134.7, 135.7, 136.9, 138.1, 138.5, 144.9, 151.0, 151.9, 154.5, 154.9, 157.2, 161.9, 168.2, 194.3. HRMS: *m/z* [M − H]^−^ calcd for C_31_H_30_BrN_7_O_8_: 706.1255, found: 706.1261.

*[2,6-Dioxo-3-[(2-[2-(3,5-dichlorobenzoyl)-4-bromophenophenoxy]ethyl]-3,6-dihydropyrimidine-1(2H)-yl]acetate of ACV* (**2g**). Purified on a silica gel column using chloroform:methanol (9:1) as eluent in 13% yield. R*_f_* 0.56 (chloroform:methanol, 95:5). ^1^H-NMR (CD_3_OD): 3.75–3.78 (2H, m, CH_2_O), 3.94–3.96 (2H, m, CH_2_N), 4.22–4.24 (4H, m, 2xCH_2_CH_2_C=O), 4.55 (2H, s, NCH_2_C=O), 5.40–5.43 (1H, d, J=12Hz, H5Ura), 5.45 (2H, s, OCH_2_N), 7.00–7.02 (1H, d, *J* = 8Hz, H6Ura), 7.07–7.10 (1H, d, J=12Hz, Ph), 7.45 (1H, m, Ph), 7.57–7.58 (2H, m, Ph), 7.63–7.64 (2H, m, Ph), 7.82 (1H, s, H8). ^13^C-NMR (DMSO-d_6_): 14.4, 22.5, 23.1, 41.8, 64.4, 66.4, 72.3, 79.65, 99.8, 113.2, 115.6, 116.9, 128.0, 129.4, 132.0, 133.4, 135.2, 135.7, 138.0, 139.8, 145.0, 151.0, 151.9, 154.7, 155.09, 157.4, 161.9, 168.1, 191.8. HRMS: *m/z* [M − H]^−^ calcd for C_29_H_24_BrCl_2_N_7_O_8_: 746.0163, found: 746.0177.

#### 3.2.4. General Method for the Synthesis of 9-(4′-hydroxy-2′cyclopenten-1′-yl)adenine Derivatives **6a–6d**

A mixture of 9-(4′-hydroxy-2′-cyclopenten-1′-yl)adenine (0.5 mmol) and the corresponding [2,6-dioxo-3-[(2-benzoylphenoxy)ethyl)]-3,6-dihydropyrimidine-1(2*H*)-yl]acetic acid **5a–5d** (0.5 mmol) was twice evaporated from DMF, then again dissolved in DMF (5 mL) and 1-ethyl-3-(3-dimethylaminopropyl)carbodiimide (0.6 mmol) and dimethylaminopyridine (0.25 mmol) were added. The mixture was stirred for 16 h at room temperature. The solvent was evaporated in vacuo, the residue was purified by silica gel column chromatography, eluting with the chloroform: methanol system (95:5). The yield of the desired esters **6a–6g** was in the range of 56–84%.

*9-(4′-[2,6-Dioxo-3-[(2-benzoylphenoxy)ethyl]-6-methyl-3,6-dihydropyrimidine-1(2H)-yl]acetyl-2′cyclo-penten-1′-yl)adenine* (**6a**). Purified on a silica gel column using chloroform: methanol (98:2) as eluent in 62% yield. R*_f_* 0.53 (chloroform:methanol, 95:5). ^1^H-NMR (CDCl_3_): 1.94 (3H, s, CH_3_), 1.98–2.02 (1H, m, H5′a), 3.04–3.14 (1H, m, 5′Hb), 4.01–4.04 (2H, m, CH_2_-N), 4.24–4.26 (2H, m, CH_2_-O), 4.57–4.69 (2H, d, *J =* 3.2 Hz, CH_2_CO), 5.25 (1H, s, H5Ura), 5.68–5.71 (1H, m, H4′), 5.81–5.83 (1H, m, H1′), 5.96 (2H, s, NH_2_), 6.18–6.21 (1H, m, H3′), 6.32–6.36 (1H, m, H2′), 6.96–6.98 (1H, d, *J* = 8.1 Hz, HPh_2_CO), 7.08–7.11 (1H, t, *J* = 7.9 Hz, HPh_2_CO), 7.31 (1H, m, HPh_2_CO), 7.40–7.45 (3H, m, HPh_2_CO), 7.58–7.60 (1H, m, HPh_2_CO), 7.77–7.79 (2H, d, *J* = 7.1 Hz, HPh_2_CO), 7.84 (1H, s, H8), 8.38 (1H, s, H2). ^13^C-NMR (CDCl_3_): 20.3, 38.4, 42.0, 44.8, 56.7, 66.0, 70.6, 78.2, 101.5, 112.3, 121.6, 128.5, 129.1, 129.9, 131.6, 133.6, 134.8, 135.0, 138.7, 152.7, 155.4, 161.1, 167.4.

*9-(4′-[2,6-Dioxo-3-[(2-benzoylphenoxy)ethyl]-5-methyl-3,6-dihydropyrimidine-1(2H)-yl]acetyl-2′cyclo-penten-1′-yl)adenine* (**6b**). Purified on a silica gel column using chloroform:methanol (98:2) as eluent in 84% yield. R*_f_* 0.55 (chloroform:methanol, 95:5). ^1^H-NMR (CDCl_3_): 1.74 (3H, s, CH_3_), 1.97–2.03 (1H, m, H5′a), 3.07–3.12 (1H, m, 5′Hb), 3.91–3.96 (2H, m, CH_2_-N), 4.19–4.21 (2H, m, CH_2_-O), 4.60–4.72 (2H, d, *J* = 1.3 Hz, CH_2_CO), 5.68–5.70 (1H, m, H4′), 5.80–5.82 (1H, m, H1′), 5.86 (2H, s, NH_2_), 6.18–6.21 (1H, m, H3′), 6.33–6.35 (1H, m, H2′), 6.75 (1H, s, H6Ura), 6.94–6.96 (1H, d, *J* = 8.3 Hz, HPh_2_CO), 7.08–7.11 (1H, t, *J* = 7.4 Hz, HPh_2_CO), 7.35 (1H, m, HPh_2_CO), 7.44–7.50 (3H, m, HPh_2_CO), 7.57–7.60 (1H, m, HPh_2_CO), 7.82–7.84 (3H, m, HPh_2_CO,H8), 8.39 (1H, s, H2). ^13^C-NMR (CDCl_3_): 12.6, 38.4, 42.2, 48.9, 56.7, 66.3, 70.6, 78.2, 109.2, 112.3, 119.7, 121.3, 128.4, 129.9, 129.9, 132.2, 133.3, 135.1, 149.7, 151.0, 152.7, 155.3, 155.8, 163.0, 167.4, 195.4.

*9-(4′-[2,6-Dioxo-3-[(2-[2-(3,5-dimethylbenzoyl)-4-chlorophenophenoxy]ethyl]-3,6-dihydropyrimidine-1(2H)-yl]acetyl-2′cyclopenten-1′-yl)adenine* (**6c**). Purified on a silica gel column using chloroform:methanol (98:2) as eluent in 58% yield. R_*f*_ 0.61 (chloroform: methanol, 95:5). ^1^H-NMR (CDCl_3_): 1.99–2.02 (1H, m, H5′a), 2.37 (6H, s, 2 × CH_3_), 3.05–3.12 (1H, m, 5′Hb), 3.92–3.95 (2H, m, CH_2_-N), 4.15–4.18 (2H, m, CH_2_-O), 4.58–4.70 (2H, d, *J* = 3.0 Hz, CH_2_CO), 5.33–5.35 (1H, d, 7.9 Hz, H5Ura), 5.70–5.72 (1H, m, H4′), 5.80–5.83 (1H, m, H1′), 5.85 (2H, s, NH_2_), 6.21–6.22 (1H, m, H3′), 6.35 (1H, m, H2′), 6.60–6.63 (1H, d, 7.9 Hz, H6Ura), 6.87–6.90 (1H, d, *J* = 8.8 Hz, HPh_2_CO), 7.27–7.33 (3H, m, HPh_2_CO), 7.34–7.40 (3H, m, HPh_2_CO), 7.84 (1H, s, HPh_2_CO) 7.82–7.84 (3H, m, HPh_2_CO,H8), 8.39 (1H, s, H2). ^13^C-NMR (CDCl_3_): 21.2, 38.4, 41.9, 49.0, 56.7, 66.8, 70.6, 78.3, 100.8, 113.7, 126.8, 127.5, 129.4, 131.5, 134.8, 135.0, 135.5, 137.2, 138.5, 138.7, 143.8, 151.1, 152.8, 154.1, 155.2, 161.9, 167.2, 194.4.

*9-(4′-[2,6-Dioxo-3-[(2-[2-(3,5-dimethylbenzoyl)-4-chlorophenophenoxy]ethyl]-6-methyl-3,6-dihydro-pyrimidine-1(2H)-yl]acetyl-2′cyclopenten-1′-yl)adenine* (**6d**). Purified on a silica gel column using chloroform:methanol (98:2) as eluent with 56% yield. R_*f*_ 0.58 (chloroform:methanol, 95:5). ^1^H-NMR (CDCl_3_): 1.74 (3H, s, CH_3_), 2.01–2.06 (1H, m, H5′a), 2.37 (6H, s, 2 × CH_3_), 3.05–3.08 (1H, m, 5′Hb), 3.94–3.96 (2H, m, CH_2_-N), 4.18–4.19 (2H, m, CH_2_-O), 4.65–4.68 (2H, d, *J* = 7.4 Hz, CH_2_CO), 5.70–5.72 (1H, m, H4′), 5.80–5.82 (1H, m, H1′), 6.20–6.23 (1H, m, H3′), 6.38–6.40 (1H, m, H2′), 6.76 (2H, s, NH_2_), 6.89–6.92 (1H, d, *J* = 8.8 Hz, H6Ura), 7.26–7.29 (3H, m, HPh_2_CO), 7.40–7.41 (3H, m, HPh_2_CO), 7.94 (1H, s, H8), 8.37 (1H, s, H2). ^13^C-NMR (CDCl_3_): 12.5, 21.2, 38.4, 42.2, 48.8, 57.1, 66.9, 70.6, 78.1, 109.3, 113.9, 119.3, 126.5, 127.7, 129.3, 131.5, 134.4, 135.5 x 2, 138.4, 139.7, 140.4, 149.3, 149.6, 151.1, 153.9, 154.3, 163.0, 167.4, 194.5.

#### 3.2.5. General Method for the Synthesis of 5′-noraristeromycin Derivatives **3a–3d**

To a solution of the corresponding 9-(4′-hydroxy-2′-cyclopenten-1′-yl)adenine derivative **6a–3d** (0.3 mmol) in a (10:1) dioxane:water mixture (10 mL), osmium tetroxide (0.01 mmol) and N-methylmorpholine oxide (2 mmol) were added. The reaction mixture was stirred/ for 3 h at the room temperature. The progress of the reaction was monitored by TLC. Then the solvents were evaporated in vacuo, the residue was purified by silica gel column chromatography, eluting with the chloroform: methanol system (95: 5). The yields of target esters **3a–3g** were in the range of 43–65%.

*[2,6-Dioxo-3-[(2-benzoylphenoxy)ethyl]-6-methyl-3,6-dihydropyrimidine-1(2H)-yl]acetate of 5′-nor-aristeromycin* (**3a**). Purified on a silica gel column using chloroform:methanol (95:5) as eluent in 60% yield. R*_f_* 0.52 (chloroform: methanol, 9:1). ^1^H-NMR (CDCl_3_:CD_3_OD): 1.91 (3H, s, CH_3_), 2.16–2.24 (1H, m, H5′a), 2.92–3.03 (1H, m, 5Hb), 3.99–4.02 (2H, m, CH_2_-N), 4.14–4.15 (1H, m, H4′), 4.20–4.23 (2H, m, CH_2_-O), 4.46–4.50 (1H, m, H1′), 4.56–4.61(2H, m, CH_2_CO), 4.79–4.82 (1H, m, H3′), 5.06–5.09 (1H, m, H2′), 5.24 (1H, s, H5Ura), 6.96–6.99 (1H, m, HPh_2_CO), 7.03–7.08 (1H, m, HPh_2_CO), 7.25–7.36 (1H, d, *J* = 5.8 Hz, HPh_2_CO), 7.40–7.43 (3H, d, *J* = 7.9 Hz, HPh_2_CO), 7.52–7.55 (1H, m, HPh_2_CO) 7.73–7.75 (2H, d, *J* = 7.2 Hz, HPh_2_CO), 7.99 (1H, s, H8), 8.25 (1H, s, H2). ^13^C-NMR (CDCl_3_:CD_3_OD): 20.1, 33.1, 42.0, 44.8, 59.1, 65.7, 74.3, 75.6, 100.9, 112.3, 121.3, 128.5, 129.0, 129.9, 131.6, 131.8, 133.8, 136.7, 139.6, 151.6, 152.0, 152.1, 155.5, 161.8, 167.3. HRMS: *m/z* [M + nH]^+^ calcd for C_32_H_31_N_7_O_8_: 642.2307, found: 642.2292; m/z[M + nNa]^+^ calcd for C_32_H_31_N_7_O_8_: 664.2126, found: 664.2112.

*[2,6-Dioxo-3-[(2-benzoylphenoxy)ethyl]-5-methyl-3,6-dihydropyrimidine-1(2H)-yl]acetate of 5′-nor-aristeromycin* (**3b**). Purified on a silica gel column using chloroform:methanol (95:5) as eluent in 43% yield. R*_f_* 0.56 (chloroform:methanol, 9:1). ^1^H-NMR (CDCl_3_:CD_3_OD): 1.65 (3H, s, CH_3_), 2.05–2.12 (1H, m, H5′a), 2.69–2.78 (1H, m, 5′Hb), 3.85–3.88 (2H, m, CH_2_-N), 3.95-3.96 (1H, m, H4′), 4.19–4.22 (2H, t, *J* = 4.9 Hz, CH_2_-O), 4.49–4.52 (1H, m, H3′), 4.55–4.59 (2H, m, CH_2_CO),4.73–4.76 (1H, m, H2′), 4.84–4.86 (1H, m, H1′), 5.20–5.22 (1H, d, *J* = 6.6 Hz, OH), 5.31–5.33 (1H, d, *J* = 4.2 Hz, OH), 7.02 (1H, s, H5Ura), 7.10–7.18 (1H, m, HPh_2_CO), 7.02–7.07 (1H, m, HPh_2_CO), 7.30–7.33 (1H, m, HPh_2_CO), 7.42–7.47 (3H, m, HPh_2_CO), 7.61–7.68 (3H, m, HPh_2_CO), 8.13–8.15 (2H, d, *J* = 5.6 Hz, H8 and H2). ^13^C-NMR (CDCl_3_:CD_3_OD): 12.3, 33.1, 42.2, 59.1, 65.1, 70.3, 74.2, 109.0, 112.4, 121.3, 128.4, 129.9, 132.3, 133.5, 137.4, 139.8, 140.9, 151.1, 152.0, 155.4, 155.8, 163.4, 167.4, 196.4. HRMS: *m/z* [M + nH]^+^ calcd for C_32_H_31_N_7_O_8_: 642.2307, found: 642.2304; *m/z* [M + nNa]^+^ calcd for C_32_H_31_N_7_O_8_: 664.2126, found: 664.2126. 

*[2,6-Dioxo-3-[(2-[2-(3,5-dimethylbenzoyl)-4-chlorophenophenoxy]ethyl]-3,6-dihydropyrimidine-1(2H)-yl]-acetate of 5′-noraristeromycin* (**3c**). Purified on a silica gel column using chloroform:methanol (95:5) as eluent in 65% yield. R_*f*_ 0.49 (chloroform: methanol, 9:1). ^1^H-NMR (DMSO-d_6_): 2.07–2.12 (1H, m, H5′a), 2.30 (6H, s, 2 × CH_3_), 2.69–2.74 (1H, m, 5′Hb), 3.87–3.90 (2H, m, CH_2_-N), 3.93–3.97 (1H, m, H4′), 4.17–4.19 (2H, m, CH_2_-O), 4.47–4.56 (3H, m, CH_2_CO and H3′), 4.73–4.76 (1H, m, H2′), 4.84–4.88 (1H, m, H1′), 5.20–5.23 (1H, m, OH), 5.32–5.36 (2H, m, OH and H5Ura), 6.96–6.99 (1H, d, *J* = 7.9 Hz, H6Ura), 7.17 (2H, s, NH_2_), 7.22–7.29 (4H, m, HPh_2_CO), 7.35 (1H, m, HPh_2_CO), 7.53–7.57 (1H, m, HPh_2_CO), 8.13–8.15 (2H, d, *J* = 6.5, H2 and H8). ^13^C-NMR (DMSO-d6): 21.2, 33.1, 48.6, 58.4, 66.5, 70.3, 74.1, 74.6, 77.7, 79.6, 99.8, 115.4, 119.9, 125.6, 127.4, 128.6, 131.0, 131.7, 135.7, 136.9, 138.5, 140.6, 144.9, 150.1, 151.0, 152.7, 154.4, 156.5, 162.0, 167.7, 194.4. HRMS: m/z [M + nH]^+^ calcd for C_33_H_32_ClN_7_O_8_: 690.2074, found: 690.2053.

*[2,6-Dioxo-3-[(2-[2-(3,5-dimethylbenzoyl)-4-chlorophenophenoxy]ethyl]-5-methyl-3,6-dihydropyrimidine-1(2H)-yl]acetate of 5′-noraristeromycin* (**3d**). Purified on a silica gel column using chloroform:methanol (95:5) as eluent with 57% yield. R_*f*_ 0.48 (chloroform: methanol, 9:1). ^1^H-NMR (DMSO-d_6_): 1.64 (3H, s, CH_3_), 2.06–2.11 (1H, m, H5′a), 2.29 (6H, s, 2 × CH_3_), 2.66–2.73 (1H, m, 5′Hb), 3.88–3.89 (2H, m, CH_2_-N), 3.95–3.97 (1H, m, H4′), 4.20–4.21 (2H, d, *J* = 4.8 Hz, CH_2_-O), 4.48–4.53 (3H, m, CH_2_CO and H3′), 4.73–4.76 (1H, m, H2′), 4.84–4.88 (1H, m, H1′), 5.21–5.23 (1H, d, *J* = 6.6 Hz, OH), 5.32–5.33 (1H, d, *J* = 4.3, OH), 7.02 (1H, s, H6Ura), 7.18 (2H, s, NH_2_), 7.24–7.28 (4H, m, HPh_2_CO), 7.32–7.33 (1H, d, *J* = 2.7 Hz, HPh_2_CO), 7.53–7.57 (1H, m, HPh_2_CO), 8.13-8.15 (2H, d, *J* = 7.1 Hz, H2 and H8). ^13^C-NMR (DMSO-d_6_): 12.7, 21.2, 33.1, 42.3, 48.4, 58.4, 66.6, 70.3, 74.1, 74.7, 77.7, 107.5, 115.6, 119.7, 125.6, 127.4, 128.5, 131.1, 131.7, 135.7, 136.8, 138.4, 140.6, 141.2, 150.2, 150.9, 152.7, 154.5, 156.5, 162.8, 167.8, 194.2. HRMS: m/z [M + nH]^+^ calcd for C_34_H_34_ClN_7_O_8_: 704.2230, found: 704.2217.

### 3.3. Hydrolysis of the Compounds by Esterase from Porcine Liver

Hydrolysis of the compounds was assayed in 40 µL 50 mM Tris-HCl buffer pH 8.2 containing NaCl 250 mM, CaCI_2_ 6 mM, esterase 8 units/test, 2 mM of compound **2d** or **3d** in methanol. The reactions were proceeded at 37 °C for 0–30 h. Reaction mixture was analyzed by TLC; in chloroform-ethanol 4:1 for **2d** (Rf of hydrolysis products: -0.8 (**2d**), 0.2 (**5d**), 0.29 (ACV)) or chloroform-ethanol 9:1 for **3d** (Rf of hydrolysis products: 0.5 (**3d**), 0.12 (**5d)**, 0.14 (5′-noraristeromycine)). The compounds **1d, 5d**, 5′-noraristeromycine and ACV were used as references.

### 3.4. Biological Assays

#### 3.4.1. Anti-HIV Activity Assays 

Inhibition of HIV-1(III_B_)- and HIV-2(ROD)-induced cytopathicity in CEM cell cultures was measured in microtiter 96-well plates containing ~3 × 10^5^ CEM cells/mL infected with 100 CCID_50_ of HIV per milliliter and containing appropriate dilutions of the test compounds. After 4–5 days of incubation at 37 °C in a CO_2_-controlled humidified atmosphere, CEM giant (syncytium) cell formation was examined microscopically. The EC_50_ (50% effective concentration) was defined as the compound concentration required to inhibit HIV-induced giant cell formation by 50%.

#### 3.4.2. Antiviral Activity Assays other than HIV

The compounds were evaluated against the following viruses: herpes simplex virus type 1 (HSV-1) strain KOS, thymidine kinase-deficient (TK^−^) HSV-1 KOS strain resistant to ACV (ACV^r^), herpes simplex virus type 2 (HSV-2) strains Lyons and G, human cytomegalovirus (HCMV) (strains AD-169 and Davis), varicella-zoster virus (strains OKA and YS), vaccinia virus Lederle strain. The antiviral assays were based on inhibition of virus-induced cytopathicity or plaque formation in human embryonic lung (HEL) fibroblasts. Confluent cell cultures in microtiter 96-well plates were inoculated with 100 CCID_50_ of virus (1 CCID_50_ being the virus dose to infect 50% of the cell cultures) or 10 or 100 plaque forming units (PFU) (for VZV and HCMV) in the presence of varying concentrations of the test compounds. Viral cytopathicity or plaque formation was recorded as soon as it reached completion in the control virus-infected cell cultures that were not treated with the test compounds. Antiviral activity was expressed as the EC_50_ or compound concentration required to reduce virus-induced cytopathogenicity or viral plaque formation by 50%.

#### 3.4.3. Cytostatic Activity Assays

All assays were performed in 96-well microtiter plates. To each well were added (5–7.5) × 10^4^ tumor cells and a given amount of the test compound. The cells were allowed to proliferate for 72 h at 37 °C in a humidified CO_2_-controlled atmosphere. At the end of the incubation period, the cells were counted in a Coulter counter. The IC_50_ (50% inhibitory concentration) was defined as the concentration of the compound that inhibited cell proliferation by 50%.

## Figures and Tables

**Table 1:** Cytotoxicity and antiviral activity against HIV (strain III_B_, SEM cells).

Compound	CC_50_, µM	IC_50_, µM	SI
**2a**	>125	49.09 ± 3.51	>3
**2b**	>125	>125	-
**2c**	>125	46.60 ± 4.02	>3
**2d**	>125	28.45 ± 2.38	>4
**2e**	>125	16.70 ± 4.83	>7
**2f**	>125	9.61 ± 0.51	>13
**2g**	>125	48.42 ± 6.23	>3
**3a**	>125	47.55 ± 3.76	>3
**3b**	8.93 ± 2.88	>8.93	<1
**3c**	48.12 ± 12	7.00 ± 0.38	>7
**3d**	18.45 ± 4.36	2.02 ± 0.29	9
**Zidovudine**	>7.48	0.022	>342
**DDN/DDI**	>211.66	8.89	>24
**Nevirapine**	>15.02	0.14	>107

**Table 2:** Cytotoxicity and antiviral activity against Herpes simplex virus type 2 in HEL cell cultures.

Compound	Cytotoxicity CC_50_,^a^ µM	HSV-2, EC_50_,^b^ µM		SI ^c^
		MTS	Visual CPE Score	
**2a**	>100	43.4	38.3	>2.3
**2b**	>100	16.35	15.85	>6.1
**2c**	>100	37.0	34.2	>2.7
**2d**	>100	14.75	14.5	>6.8
**2e**	>100	9.4	10.9	>10.6
**2f**	>100	21.8	16.25	>4.6
**2g**	>100	41.4	45.0	>2.2
**ACV**	>100	1.8	1.6	>55.5

^a^ 50% Cytotoxic concentration, as determined by measuring the cell viability with the colorimetric formazan-based MTS assay. Microscopically detectable alteration of normal cell morphology was not found for all the compounds **2a–2g** at concentration up to 100 µM. ^b^ 50% Effective concentration, or concentration producing 50% inhibition of virus-induced cytopathic effect, as determined by visual scoring of the CPE, or by measuring the cell viability with the colorimetric formazan-based MTS assay. ^c^ Selectivity index: ratio of CC_50_ to EC_50_(MTS).

**Table 3:** Cytotoxicity and antiviral activity against varicella-zoster virus (VZV) and human cytomegalovirus (CMV) in HEL cells.

Compound	Cytotoxicity, µM		VZV			CMV		
			EC_50_,^c^ µM		SI ^d^ TK^+^ Strain	EC_50_,^c^ µM		SI ^d^ Davis Strain
	CC_50_^a^	MCC ^b^	TK^+^ Strain	TK^-^ Strain		AD-169 Strain	Davis Strain	
**2b**	>100	>100	54.0	>100	>1.8	>100	>100	-
**2f**	>100	100	31.8	>100	>3	>100	68.9	>1.5
**2g**	>100	100	>20	>100	<5	>100	>100	-
**3b**	>100	>100	>100	>100	-	76.5	34.2	>2.9
**3c**	54	100	35.0	55.4	1.5	100	44.7	1.2
**3d**	38.0	100	37.1	>20	1	54.7	11.5	3.3
**ACV**	>100	>100	7.7	57.7	>13	>100	37.0	>2.7
**Gancyclovir**	>300	>300	-	-	-	19.3	1.8	>167
**Brivudin**	>300	>300	0.034	0.42	>8824	-	-	-

^a^ 50% Cytotoxic concentration, as determined by measuring the cell viability with the colorimetric formazan-based MTS assay. ^b^ Minimum cytotoxic concentration that causes a microscopically detectable alteration of cell morphology. ^c^ Effective concentration required to reduce virus plaque formation by 50%. Virus input was 100 plaque forming units (PFU). ^d^ Selectivity index: ratio of CC_50_ to EC_50_(MTS).

**Table 4:** Cytotoxicity and antiviral activity against Vaccinia virus in HEL cells.

Compound	Cytotoxicity, µM		Antiviral EC_50_,^c^ µM		SI ^d^
	CC_50_^a^	MCC ^b^	MTS	Visual CPE Score	
**2f**	>100	>100	25.4	47.8	>3.9
**3b**	>100	>100	5.5	20	>18.2
**3c**	54	100	>100	>100	-
**3d**	38	100	3.2	9.5	11.9
**(-)-5′-Noraristeromycin**	>100	>100	0.4	0.4	>250

^a^ 50% Cytotoxic concentration, as determined by measuring the cell viability with the colorimetric formazan-based MTS assay. ^b^ Minimum compound concentration that causes a microscopically detectable alteration of normal cell morphology. ^c^ 50% Effective concentration, or concentration producing 50% inhibition of virus-induced cytopathic effect, as determined by visual scoring the CPE, or by measuring the cell viability with the colorimetric formazan-based MTS assay. ^d^ Selectivity index: ratio of CC_50_ to EC_50_(MTS).

## Supplementary Materials

The Supplementary Materials are available online.
