# Supplementary material for: Uracil-Containing Heterodimers of a New Type: Synthesis and Study of Their Anti-Viral Properties
Source: Molecules. 2020 Jul 23;25(15):3350. doi: 10.3390/molecules25153350 (PMC7435738; doi:10.3390/molecules25153350)
Supplement: Supplementary file 1 [file molecules-25-03350-s001.pdf]

Supplementary material

URACIL-CONTAINING HETERODIMERS OF A NEW TYPE: SYNTHESIS AND STUDY OF ANTI-VIRAL PROPERTIES

Anna A. Maslova[1], Elena S. Matyugina[1], Robert Snoeck[2], Graciela Andrei[2], Sergey N. Kochetkov[1], Anastasia L. Khandazhinskaya[1,*] and Mikhail S. Novikov[3]

[1] Engelhardt Institute of Molecular Biology, Russian Academy of Science, Moscow 119991, Russian Federation

[2] Rega Institute for Medical Research, KU Leuven, B-3000 Leuven, Belgium

[3] Department of Pharmaceutical & Toxicological Chemistry, Volgograd State Medical University, 400131 Volgograd, Russian Federation

*Correspondence: khandazhinskaya@bk.ru

Contents

Figure S1. ^{1}H NMR spectrum of compound **4a** (in DMSO-d_6 at 400 MHz) 3
Figure S2. ^{13}C NMR spectrum of compound **4a** (in DMSO-d_6 at 100 MHz) 4
Figure S3. ^{1}H NMR spectrum of compound **4b** (in DMSO-d_6 at 400 MHz) 5
Figure S4. ^{13}C NMR spectrum of compound **4b** (in DMSO-d_6 at 100 MHz) 6
Figure S5. ^{1}H NMR spectrum of compound **4c** (in DMSO-d_6 at 400 MHz) 7
Figure S6. ^{13}C NMR spectrum of compound **4c** (in DMSO-d_6 at 100 MHz) 8
Figure S7. ^{1}H NMR spectrum of compound **4d** (in $CDCl_3$ at 400 MHz) 9
Figure S8. ^{13}C NMR spectrum of compound **4d** (in $CDCl_3$ at 100 MHz) 10
Figure S9. ^{1}H NMR spectrum of compound **4f** (in DMSO-d_6 at 400 MHz) 11
Figure S10. ^{13}C NMR spectrum of compound **4f** (in DMSO-d_6 at 100 MHz) 12
Figure S11. ^{1}H NMR spectrum of compound **4g** (in DMSO-d_6 at 400 MHz) 13
Figure S12. ^{13}C NMR spectrum of compound **4g** (in DMSO-d_6 at 100 MHz) 14
Figure S13. ^{1}H NMR spectrum of compound **5a** (in DMSO-d_6 at 400 MHz) 15
Figure S14. ^{13}C NMR spectrum of compound **5a** (in DMSO-d_6 at 100 MHz) 16
Figure S15. ^{1}H NMR spectrum of compound **5b** (in DMSO-d_6 at 400 MHz) 17
Figure S16. ^{13}C NMR spectrum of compound **5b** (in DMSO-d_6 at 100 MHz) 18
Figure S17. ^{1}H NMR spectrum of compound **5c** (in DMSO-d_6 at 400 MHz) 19
Figure S18. ^{13}C NMR spectrum of compound **5c** (in DMSO-d_6 at 100 MHz) 20
Figure S19. ^{1}H NMR spectrum of compound **5d** (in DMSO-d_6 at 400 MHz) 21
Figure S20. ^{13}C NMR spectrum of compound **5d** (in DMSO-d_6 at 100 MHz) 22
Figure S21. ^{1}H NMR spectrum of compound **5e** (in DMSO-d_6 at 400 MHz) 23
Figure S22. ^{13}C NMR spectrum of compound **5e** (in DMSO-d_6 at 100 MHz) 24
Figure S23. ^{1}H NMR spectrum of compound **5f** (in DMSO-d_6 at 400 MHz) 25
Figure S24. ^{13}C NMR spectrum of compound **5f** (in DMSO-d_6 at 100 MHz) 26
Figure S25. ^{1}H NMR spectrum of compound **5g** (in DMSO-d_6 at 400 MHz) 27
Figure S26. ^{13}C NMR spectrum of compound **5g** (in DMSO-d_6 at 100 MHz) 28
Figure S27. ^{1}H NMR spectrum of compound **2a** (in CD_3OD at 400 MHz) 29
Figure S28. ^{13}C NMR spectrum of compound **2a** (in DMSO-d_6 at 100 MHz) 30
Figure S29. ^{1}H NMR spectrum of compound **2b** (in CD_3OD at 400 MHz) 31
Figure S30. ^{13}C NMR spectrum of compound **2b** (in DMSO-d_6 at 100 MHz) 32
Figure S31. ^{1}H NMR spectrum of compound **2c** (in CD_3OD at 400 MHz) 33
Figure S32. ^{13}C NMR spectrum of compound **2c** (in CD_3OD at 100 MHz) 34
Figure S33. ^{1}H NMR spectrum of compound **2d** (in DMSO-d_6 at 400 MHz) 35
Figure S34. ^{13}C NMR spectrum of compound **2d** (in DMSO-d_6 at 100 MHz) 36
Figure S35. ^{1}H NMR spectrum of compound **2e** (in DMSO-d_6 at 400 MHz) 37
Figure S36. ^{13}C NMR spectrum of compound **2e** (in DMSO-d_6 at 100 MHz) 38
Figure S37. ^{1}H NMR spectrum of compound **2f** (in CD_3OD at 400 MHz) 39
Figure S38. ^{13}C NMR spectrum of compound **2f** (in DMSO-d_6 at 100 MHz) 40
Figure S39. ^{1}H NMR spectrum of compound **2g** (in CD_3OD at 400 MHz) 41
Figure S40. ^{13}C NMR spectrum of compound **2g** (in DMSO-d_6 at 100 MHz) 42
Figure S41. ^{1}H NMR spectrum of compound **6a** (in $CDCl_3$ at 400 MHz) 43
Figure S42. ^{13}C NMR spectrum of compound **6a** (in $CDCl_3$ at 100 MHz) 44
Figure S43. ^{1}H NMR spectrum of compound **6b** (in $CDCl_3$ at 400 MHz) 45
Figure S44. ^{13}C NMR spectrum of compound **6b** (in $CDCl_3$ at 100 MHz) 46
Figure S45. ^{1}H NMR spectrum of compound **6c** (in $CDCl_3$ at 400 MHz) 47
Figure S46. ^{13}C NMR spectrum of compound **6c** (in $CDCl_3$ at 100 MHz) 48
Figure S47. ^{1}H NMR spectrum of compound **6d** (in $CDCl_3$ at 400 MHz) 49
Figure S48. ^{13}C NMR spectrum of compound **6d** (in $CDCl_3$ at 100 MHz) 50
Figure S49. ^{1}H NMR spectrum of compound **3a** (in $CDCl_3$:CD_3OD at 400 MHz) 51
Figure S50. ^{13}C NMR spectrum of compound **3a** (in $CDCl_3$:CD_3OD at 100 MHz) 52
Figure S51. ^{1}H NMR spectrum of compound **3b** (in $CDCl_3$:CD_3OD at 400 MHz) 53
Figure S52. ^{13}C NMR spectrum of compound **3b** (in DMSO-d_6 at 100 MHz) 54
Figure S53. ^{1}H NMR spectrum of compound **3c** (in DMSO-d_6 at 400 MHz) 55
Figure S54. ^{13}C NMR spectrum of compound **3c** (in DMSO-d_6 at 100 MHz) 56
Figure S55. ^{1}H NMR spectrum of compound **3d** (in DMSO-d_6 at 400 MHz) 57
Figure S56. ^{13}C NMR spectrum of compound **3d** (in DMSO-d_6 at 100 MHz) 58

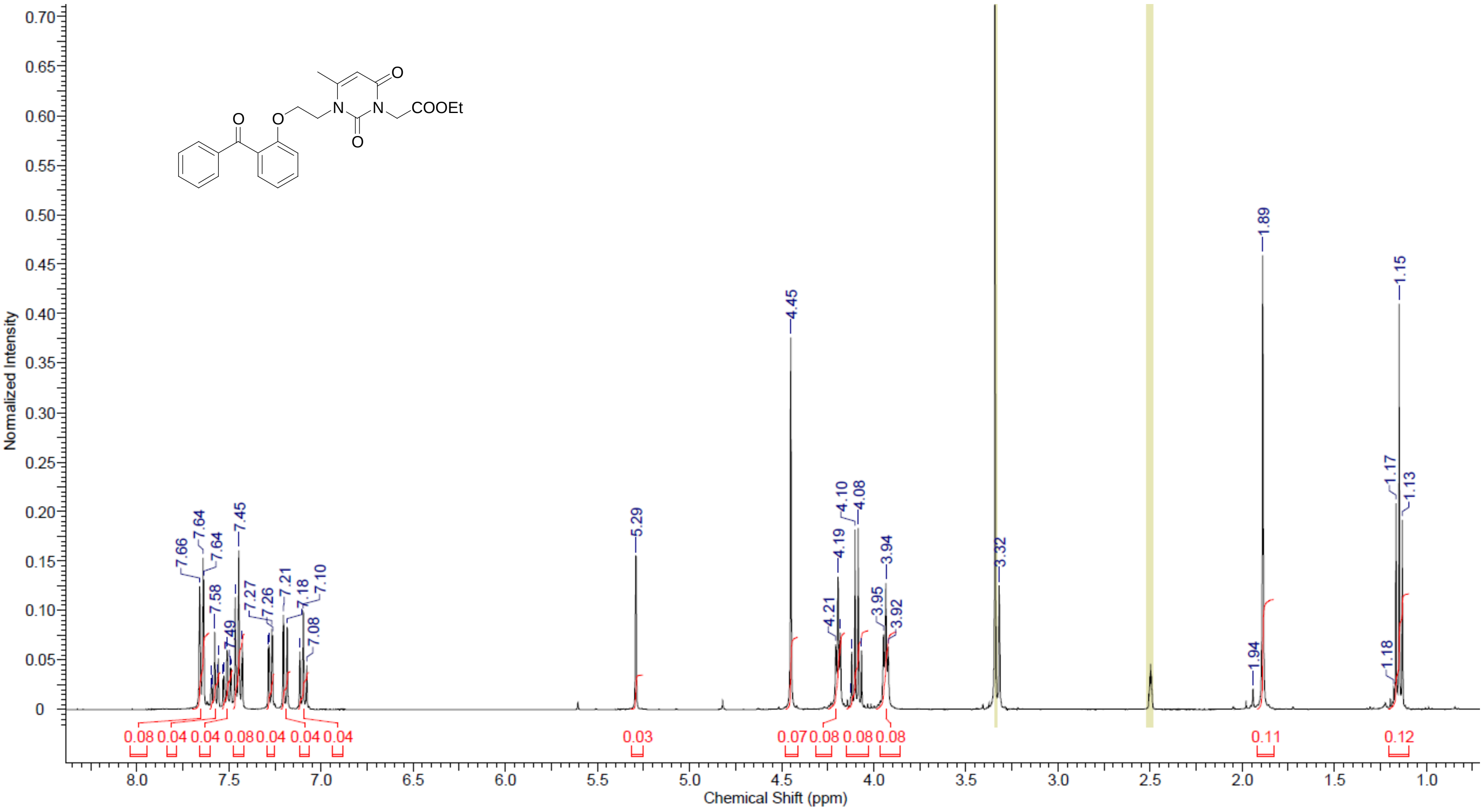

Figure S1 ^{1}H NMR spectrum of compound **4a** in DMSO-d_6 at 400 MHz.

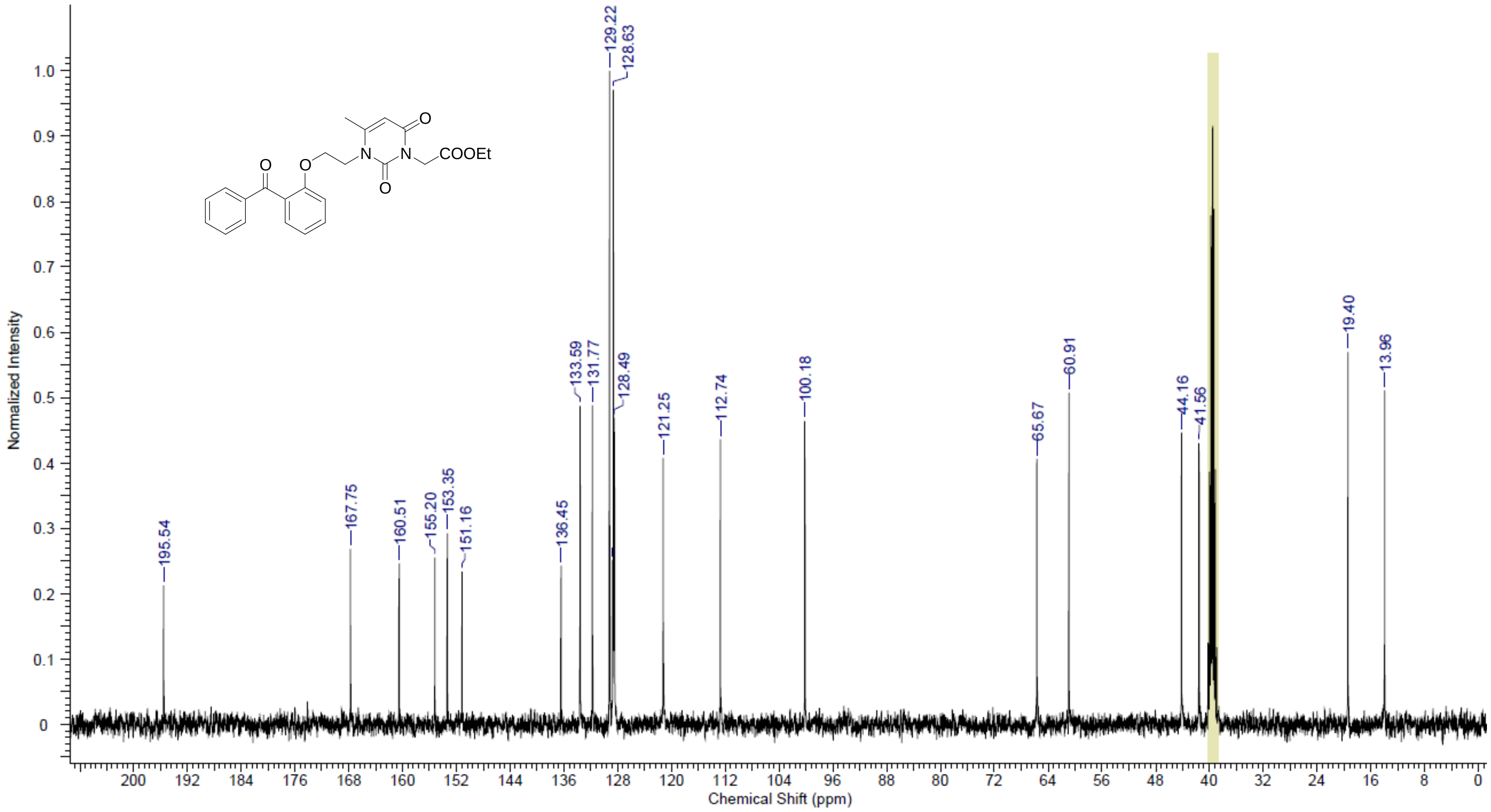

Figure S2 ^{13}C NMR spectrum of compound **4a** in DMSO-d_6 at 100 MHz.

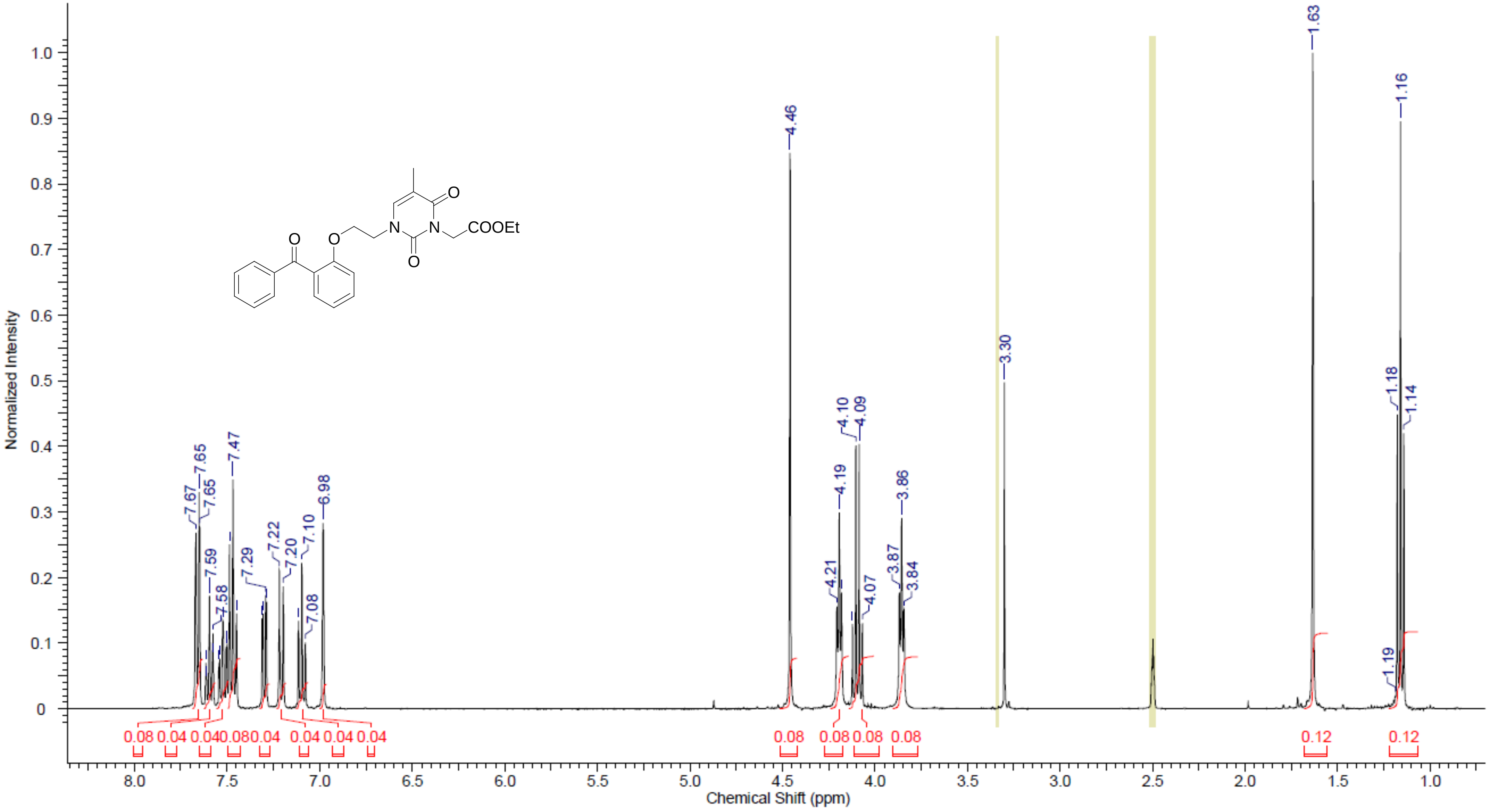

Figure S3. ^{1}H NMR spectrum of compound **4b** in DMSO-d_6 at 400 MHz.

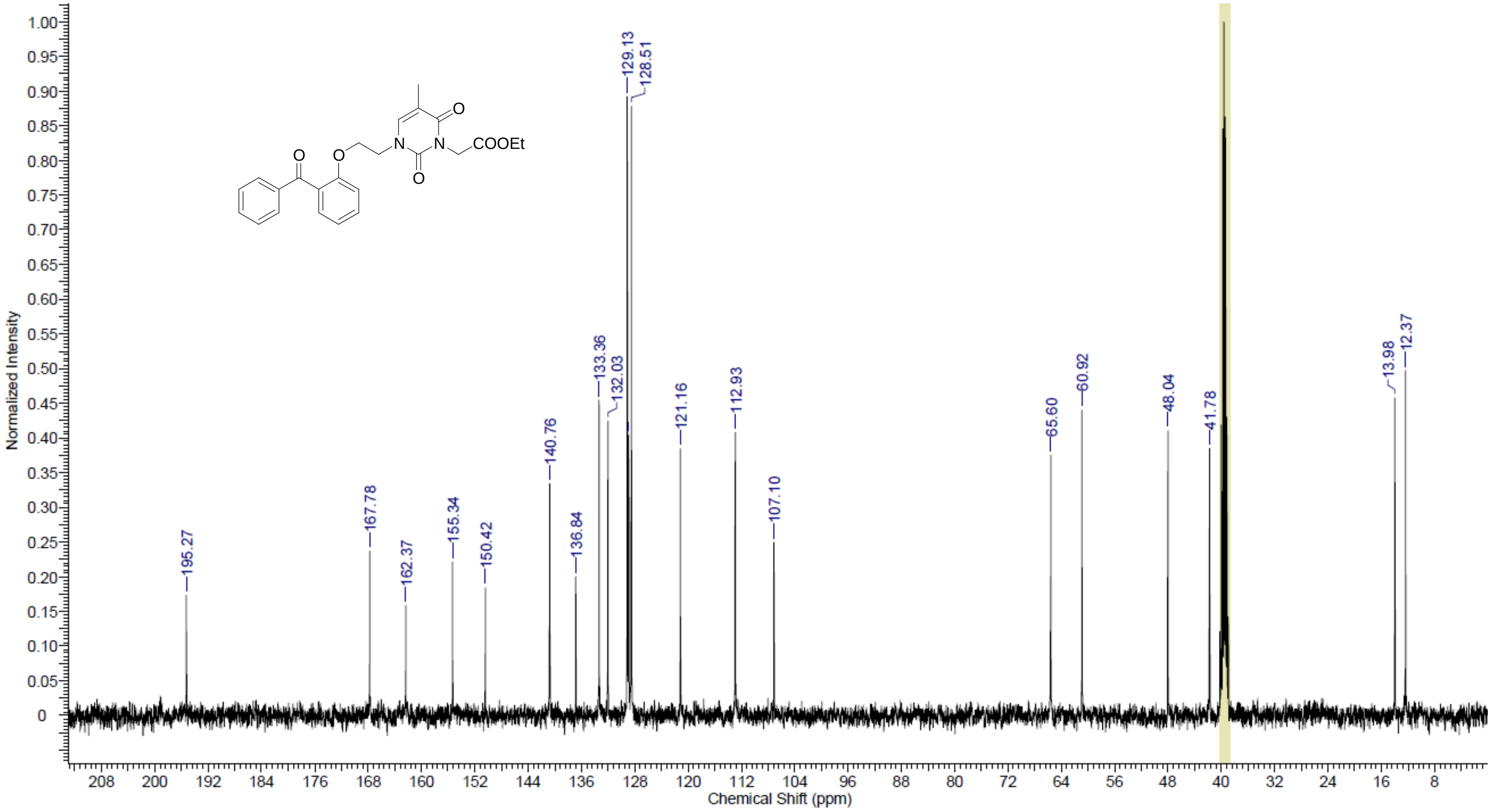

Figure S4. ^{13}C NMR spectrum of compound **4b** in DMSO-d_6 at 100 MHz.

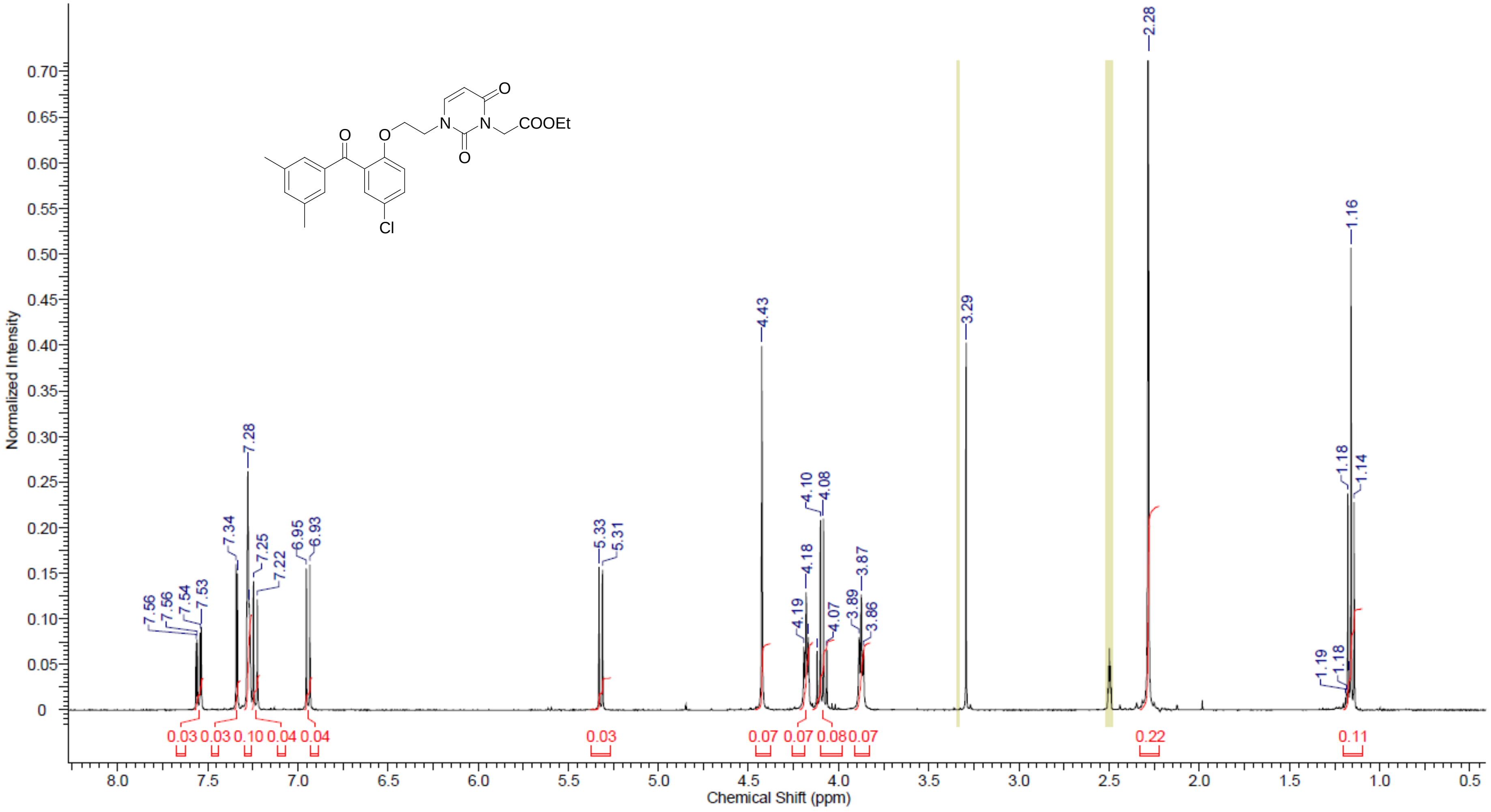

Figure S5. ^{1}H NMR spectrum of compound **4c** in DMSO-d_6 at 400 MHz.

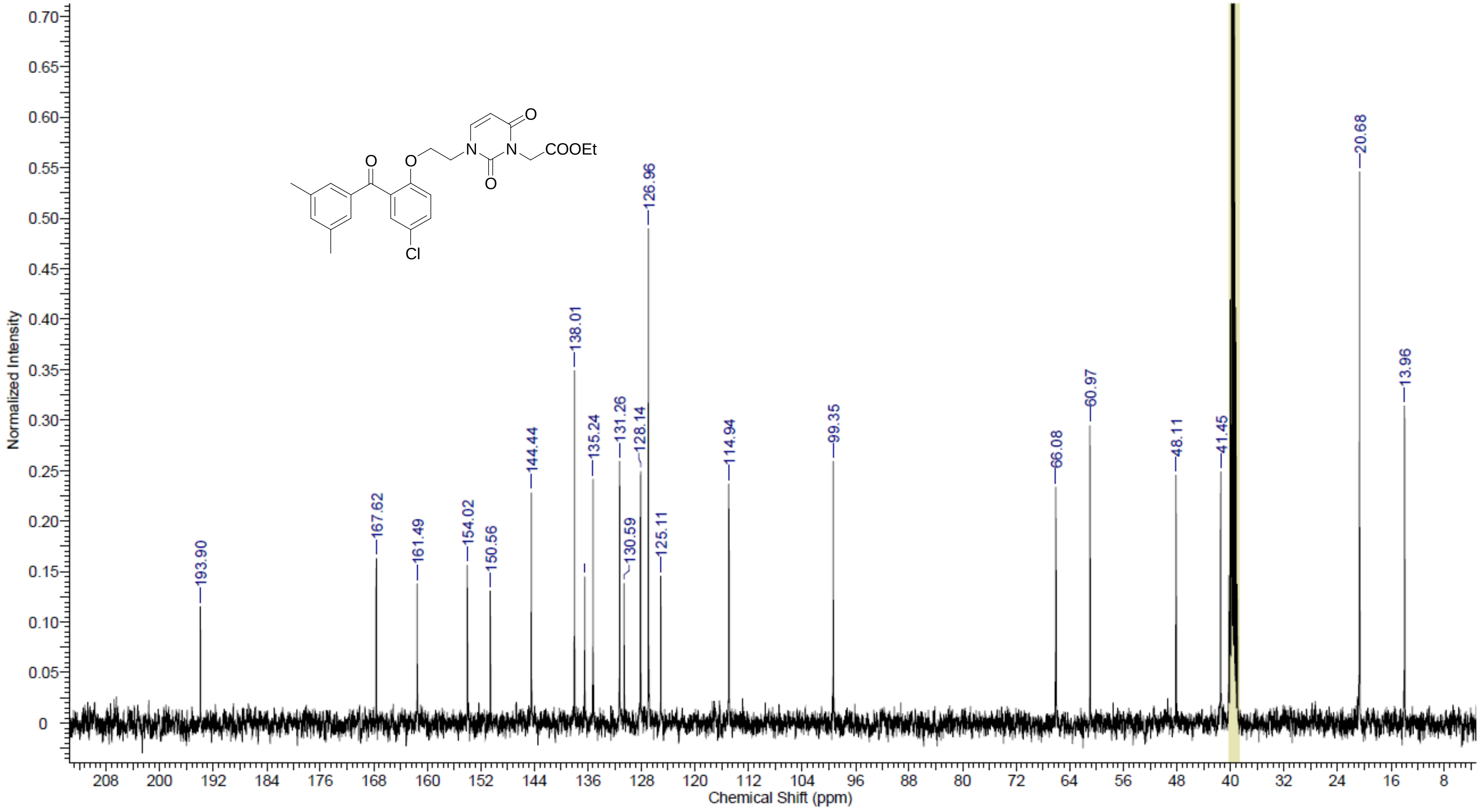

Figure S6. ^{13}C NMR spectrum of compound **4c** in DMSO-d_6 at 100 MHz.

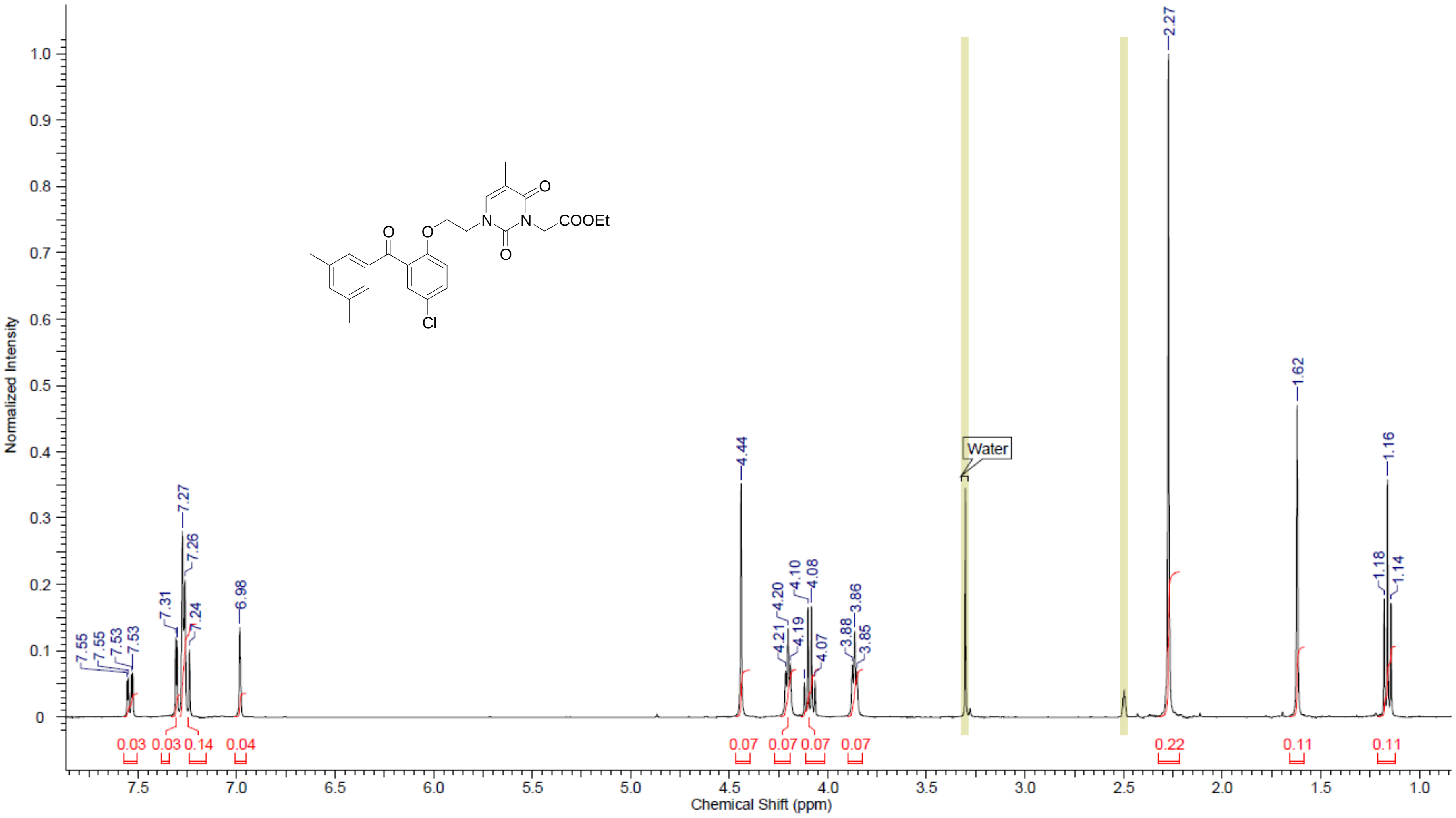

Figure S7. ^{1}H NMR spectrum of compound **4d** in $CDCl_3$ at 400 MHz.

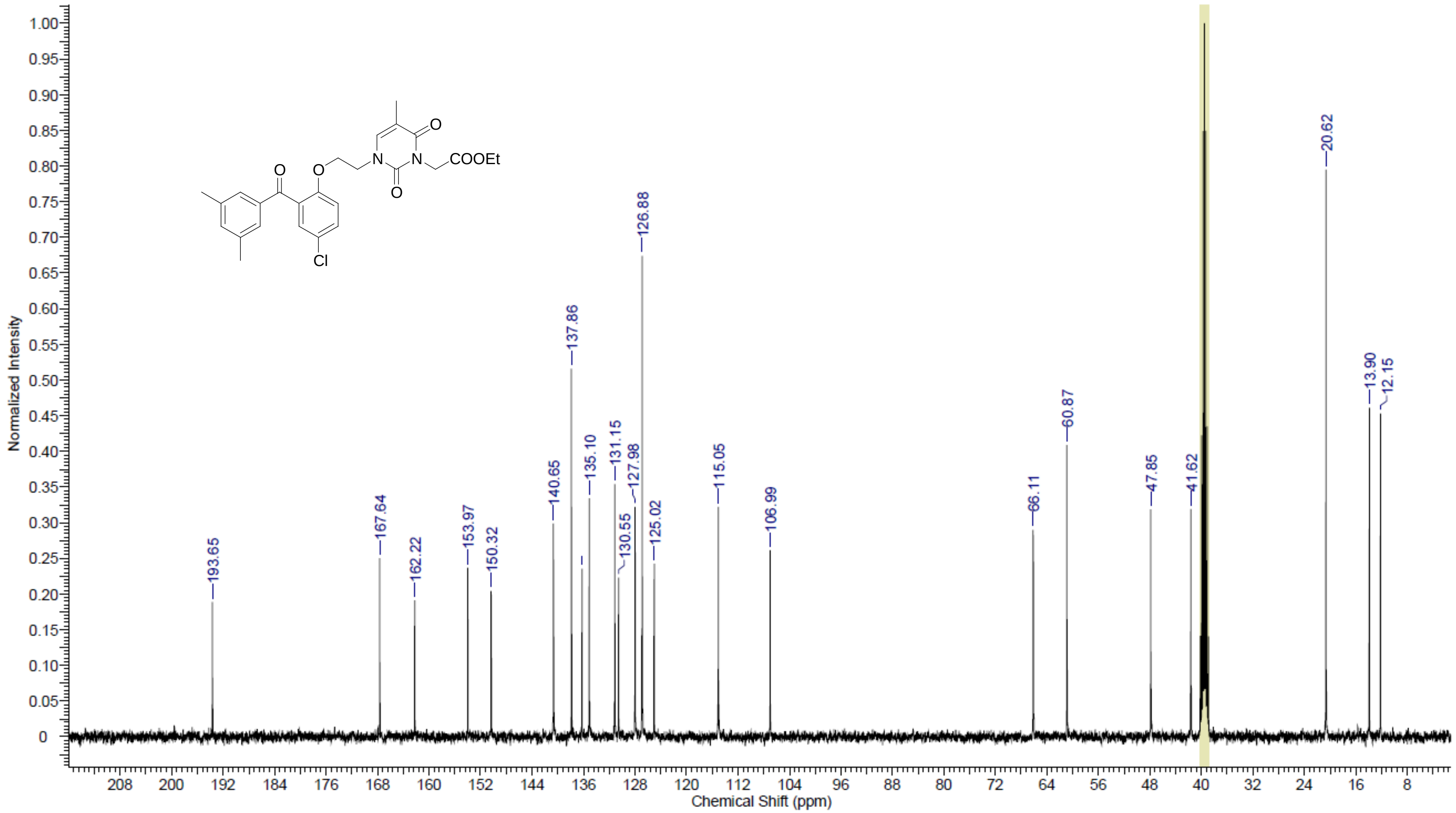

Figure S8. ^{13}C NMR spectrum of compound **4d** in $CDCl_3$ at 100 MHz.

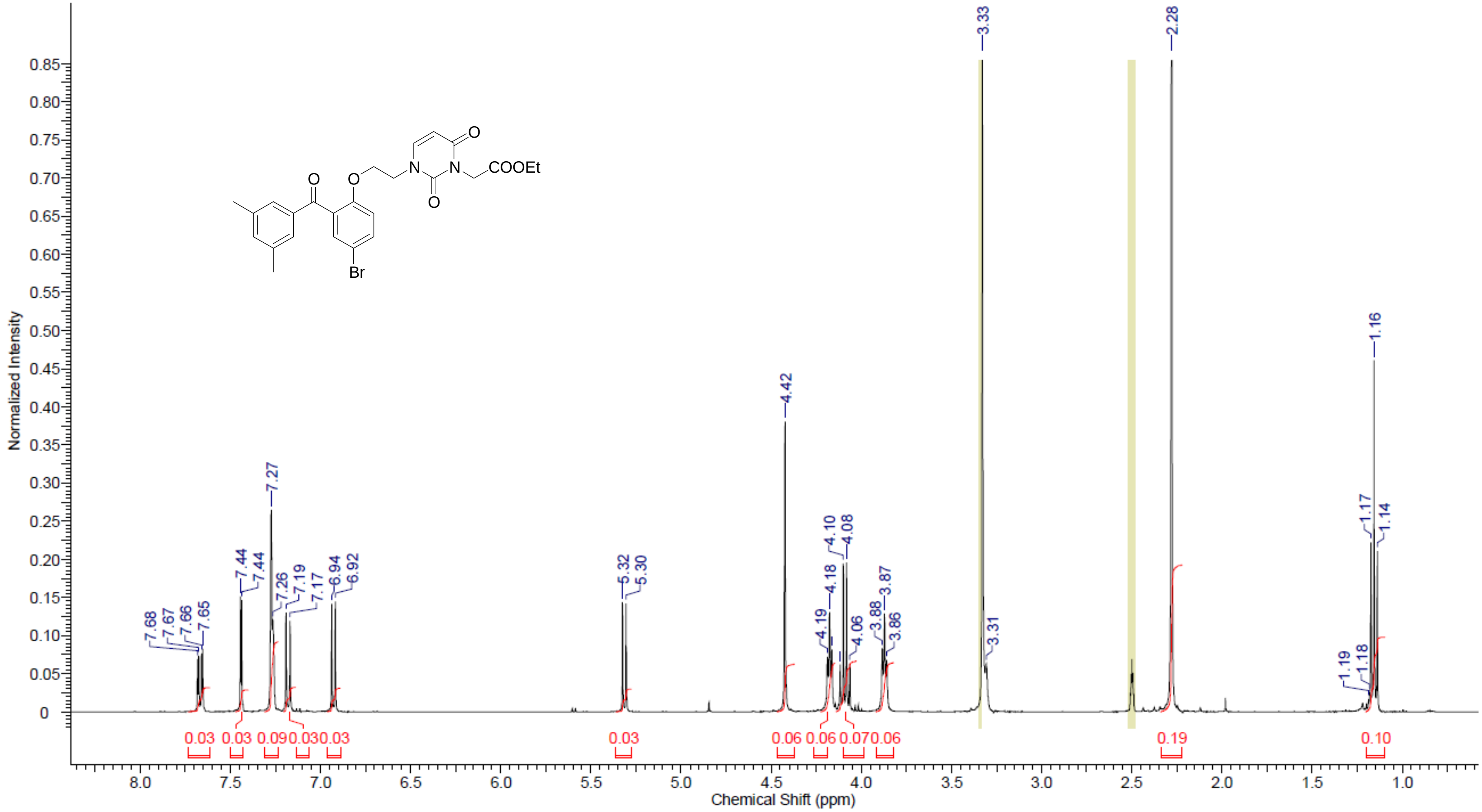

Figure S9. ^{1}H NMR spectrum of compound **4f** in DMSO-d_6 at 400 MHz.

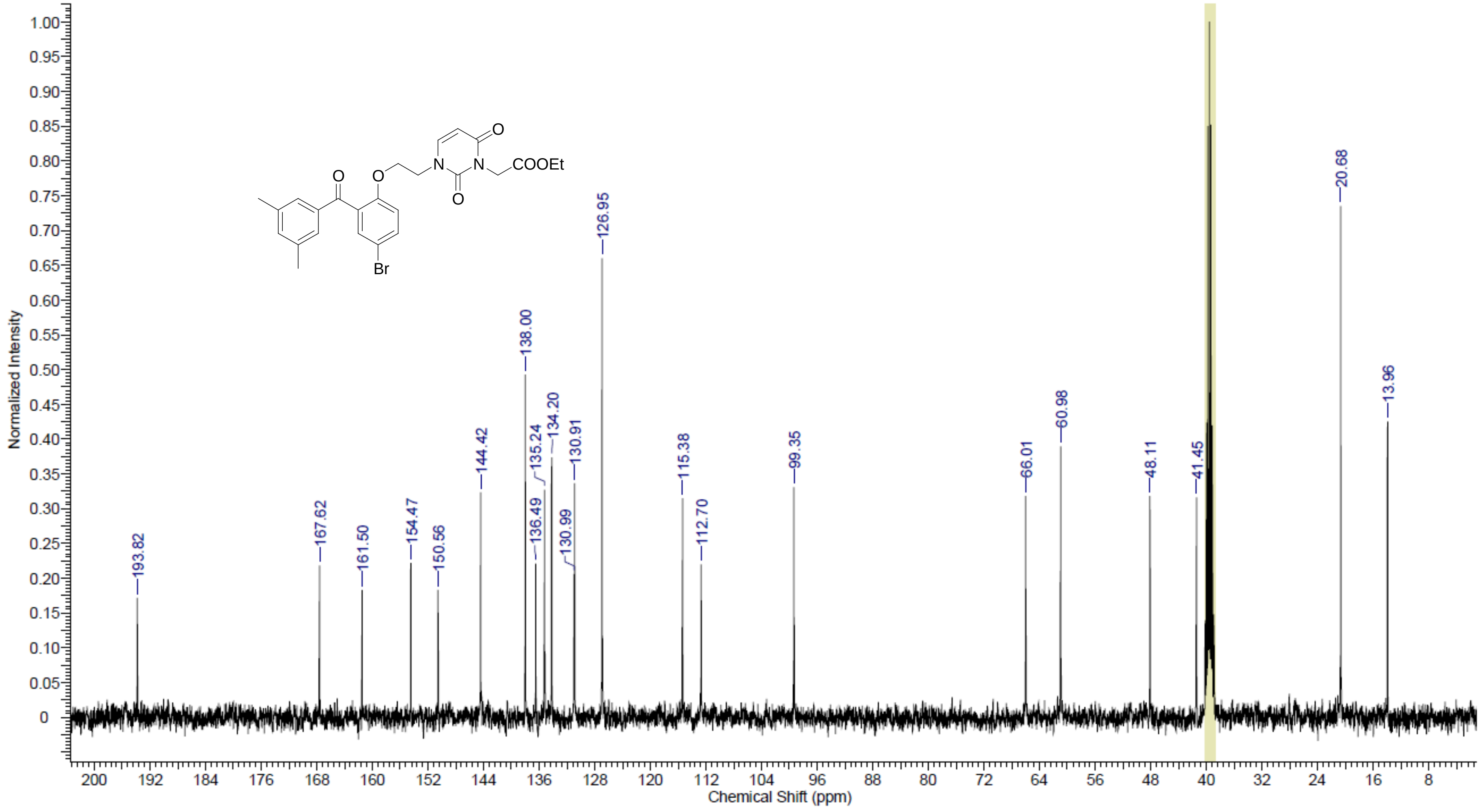

Figure S10. ^{13}C NMR spectrum of compound **4f** in DMSO-d_6 at 100 MHz.

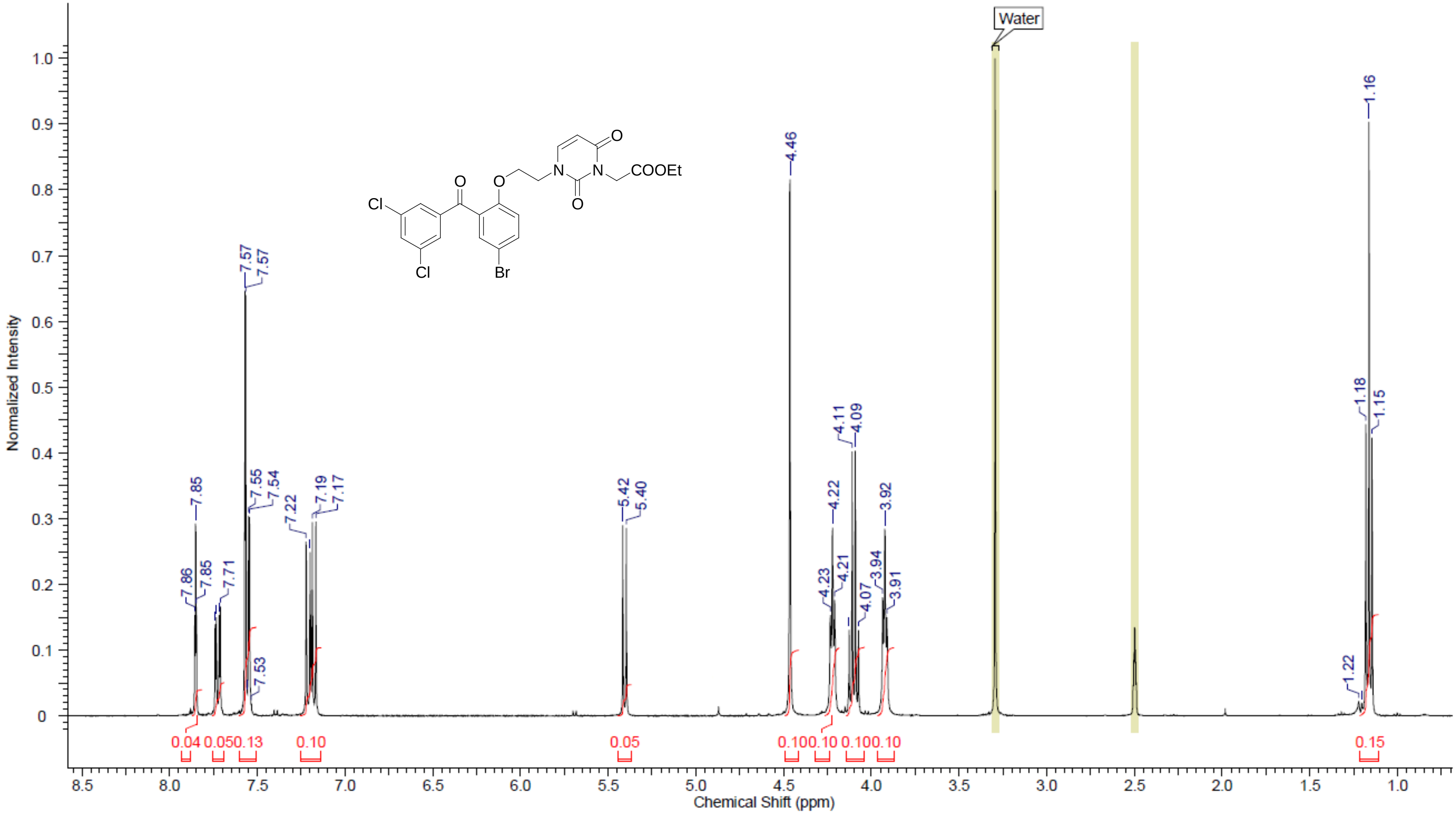

Figure S11. ^{1}H NMR spectrum of compound **4g** in DMSO-d_6 at 400 MHz.

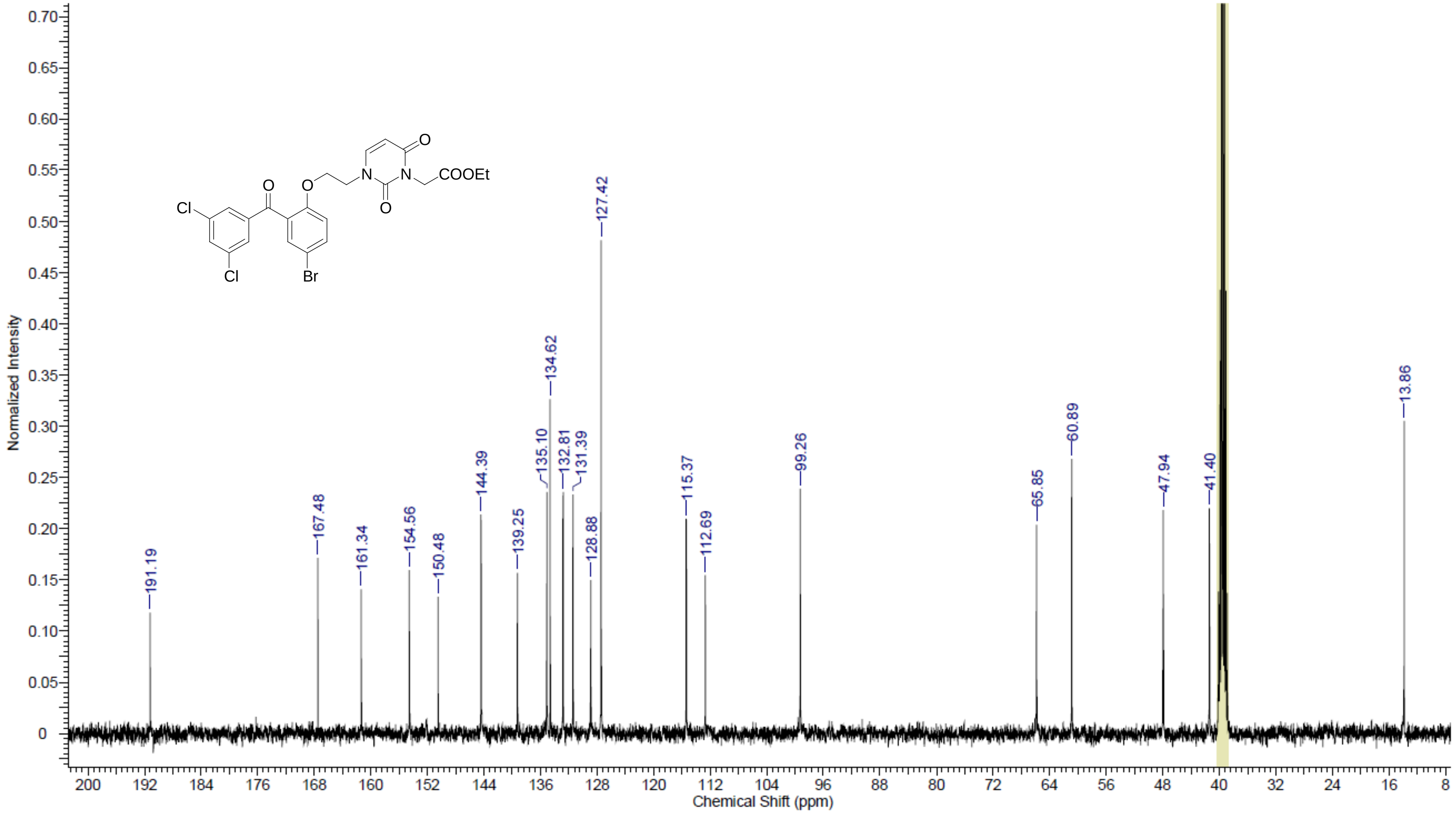

Figure S12. ^{13}C NMR spectrum of compound **4g** in DMSO-d_6 at 100 MHz.

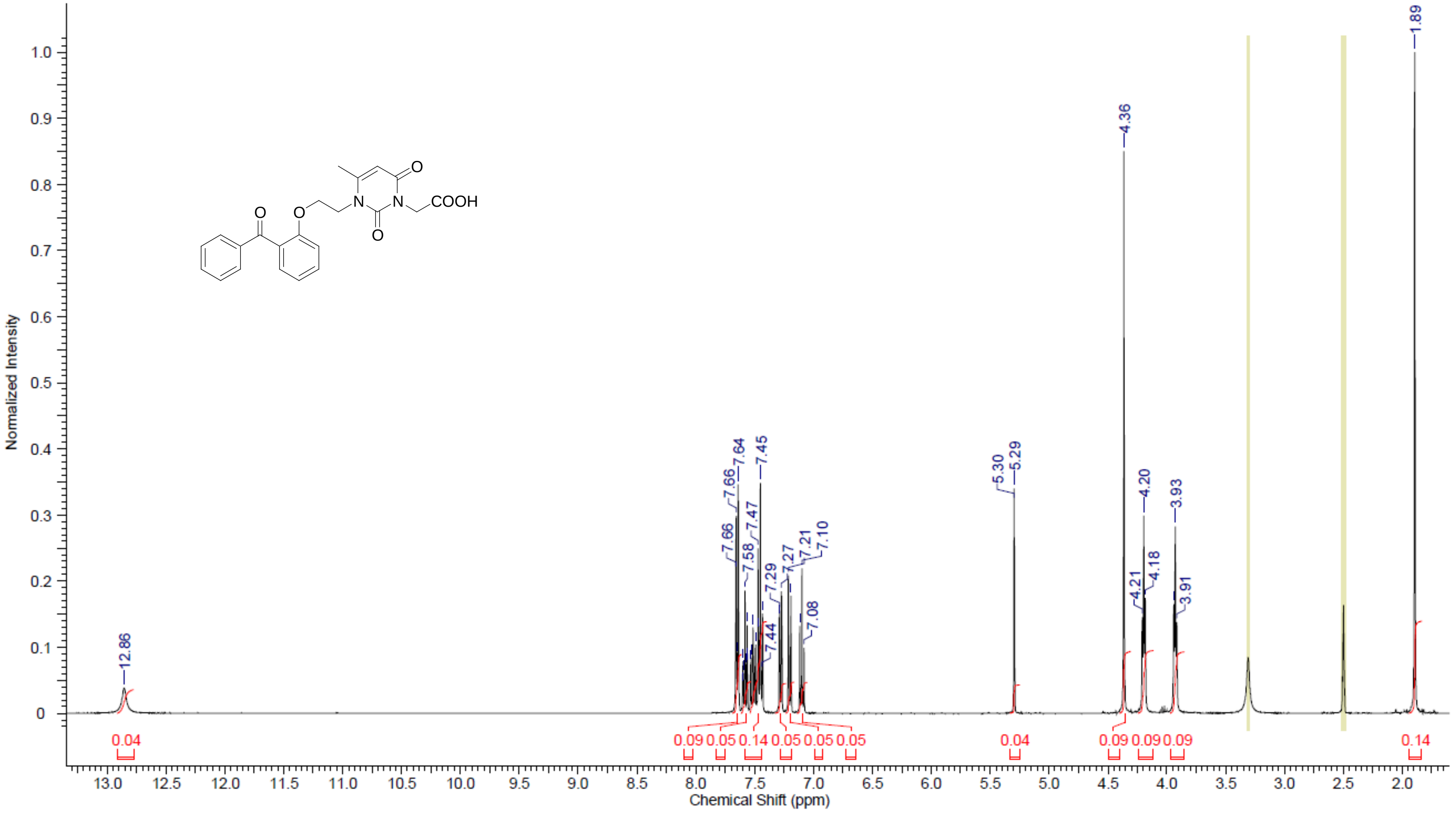

Figure S13. ^{1}H NMR spectrum of compound **5a** in DMSO-d_6 at 400 MHz.

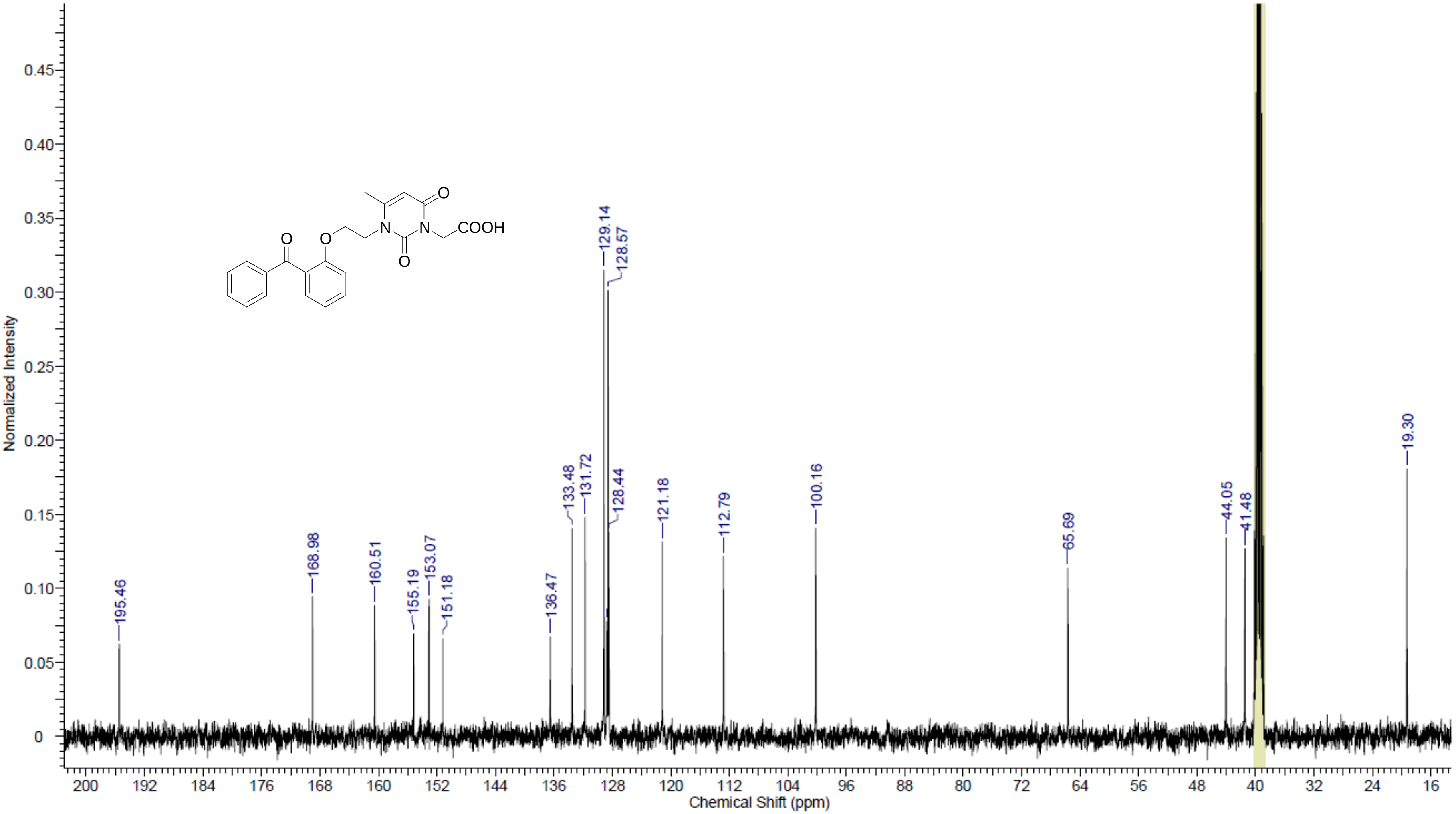

Figure S14. ^{13}C NMR spectrum of compound **5a** in DMSO-d_6 at 100 MHz.

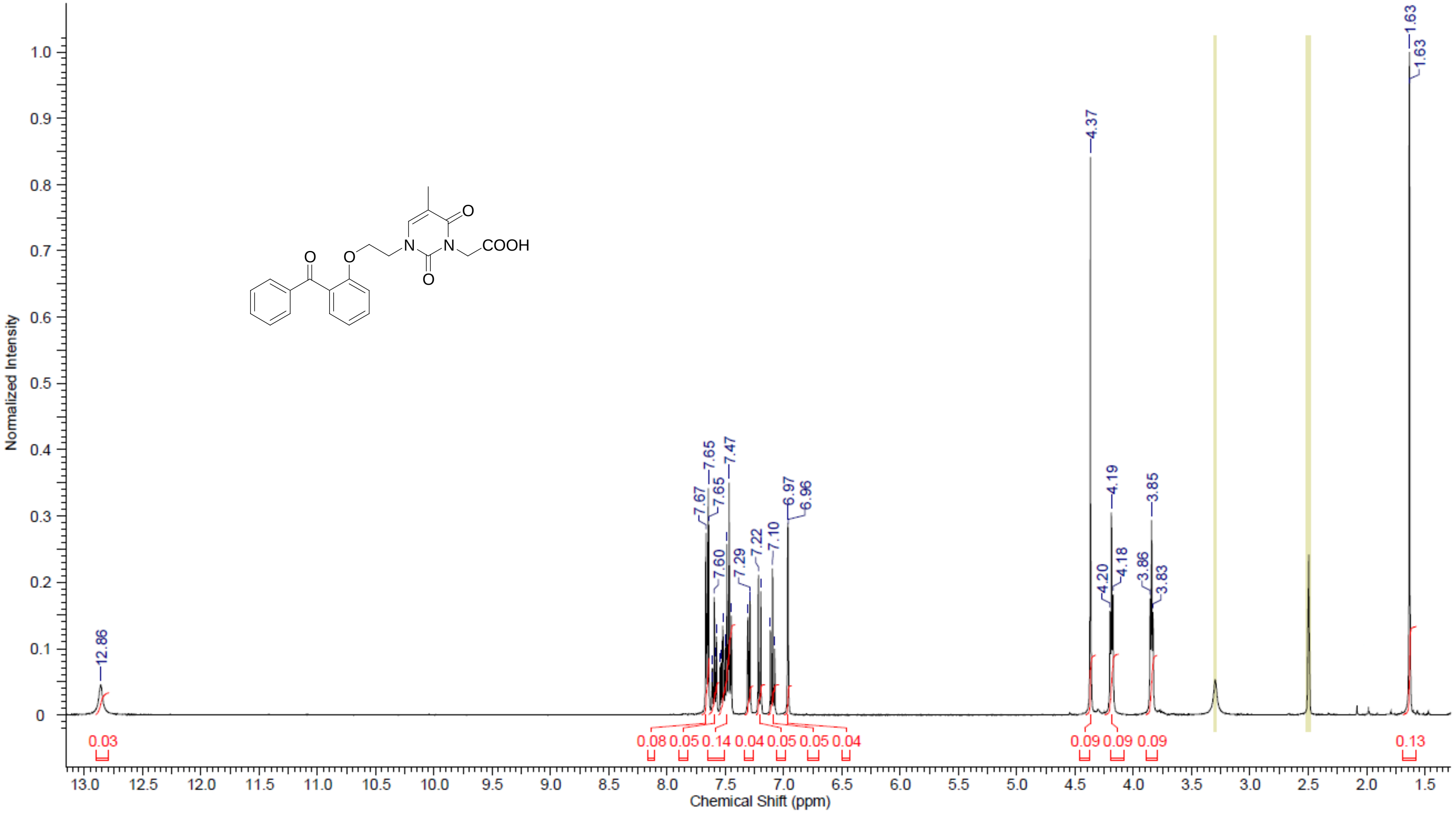

Figure S15. ^{1}H NMR spectrum of compound **5b** in DMSO-d_6 at 400 MHz.

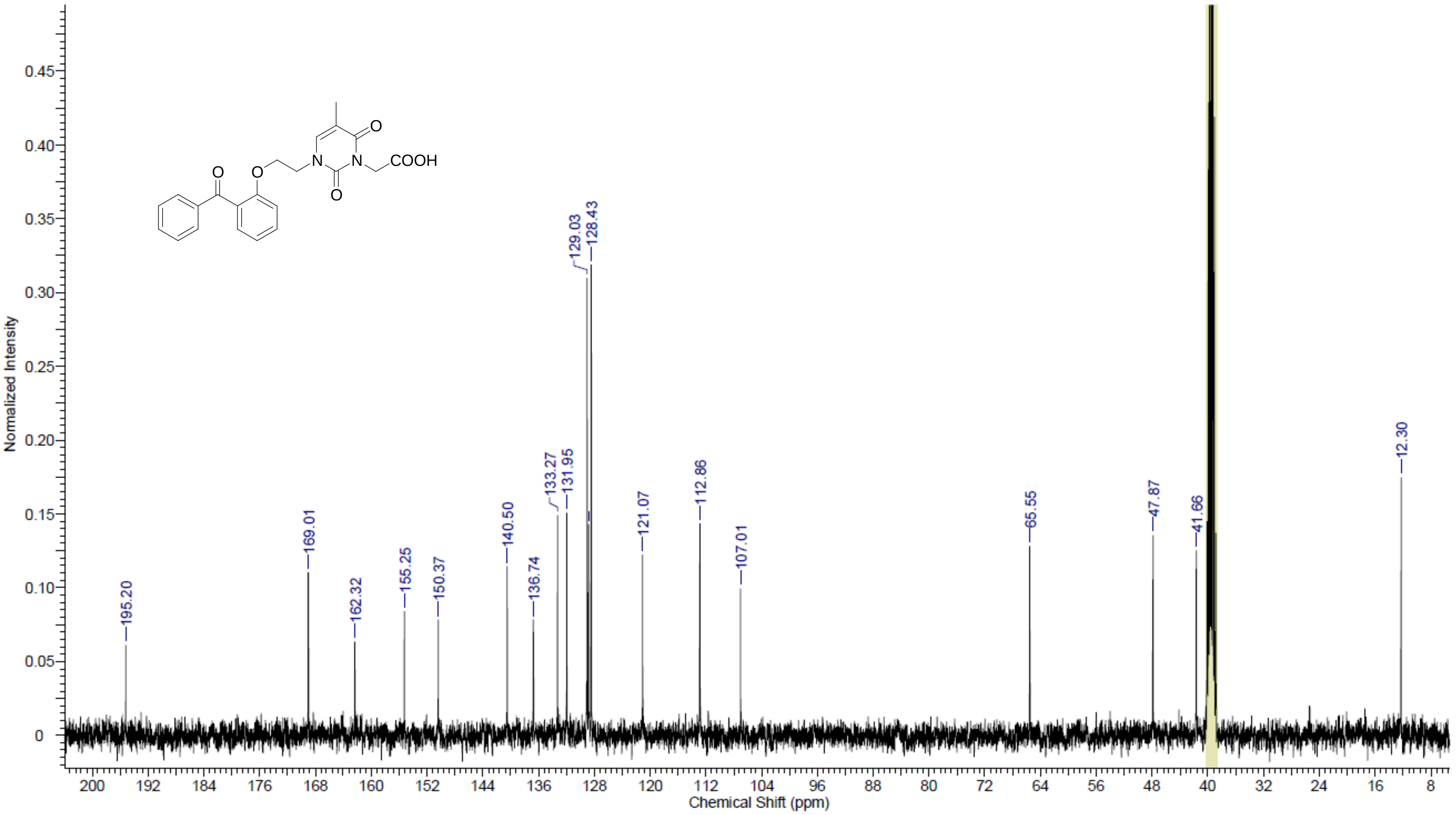

Figure S16. ^{13}C NMR spectrum of compound **5b** in DMSO-d_6 at 100 MHz.

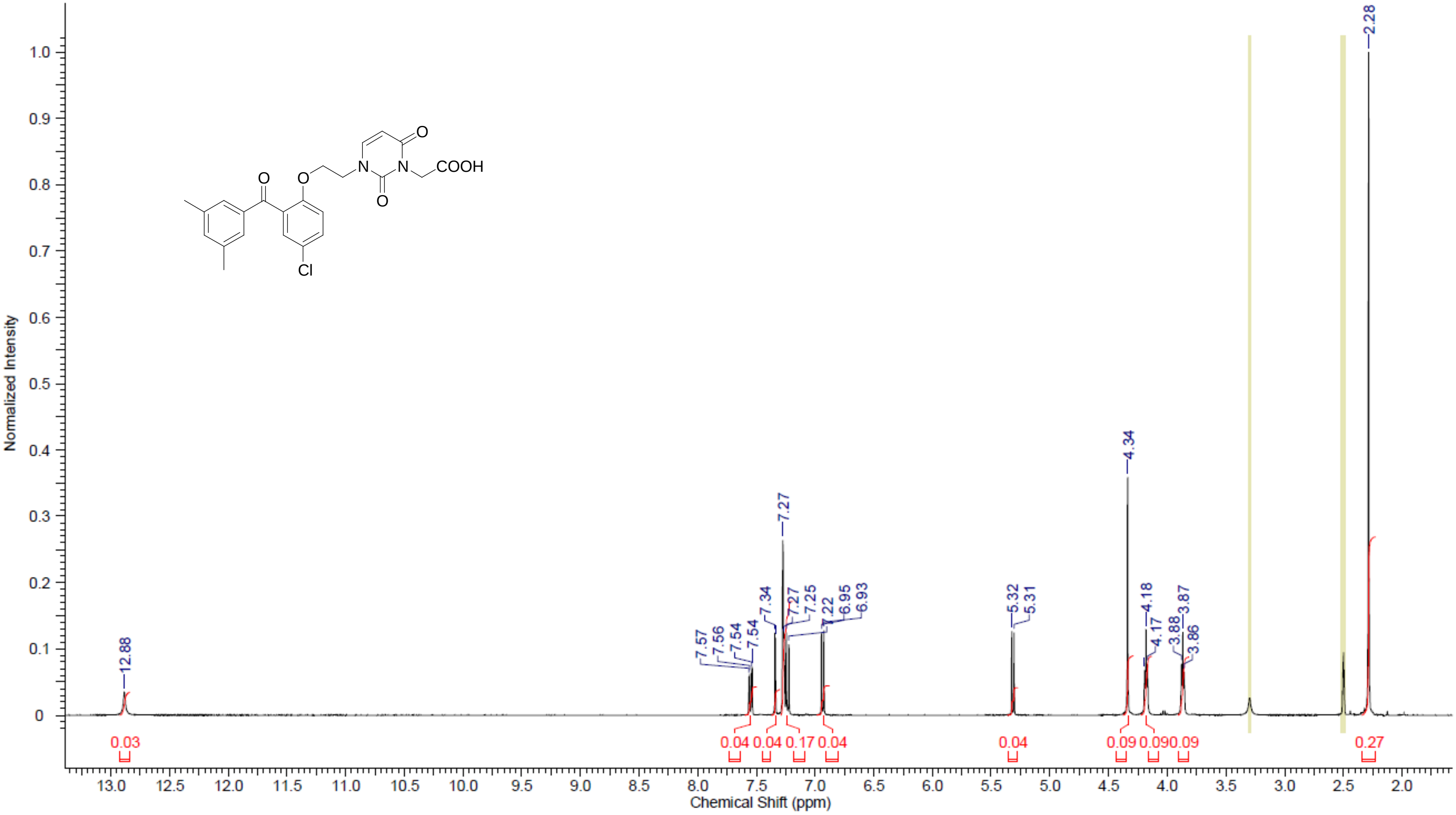

Figure S17. ^{1}H NMR spectrum of compound **5c** in DMSO-d_6 at 400 MHz.

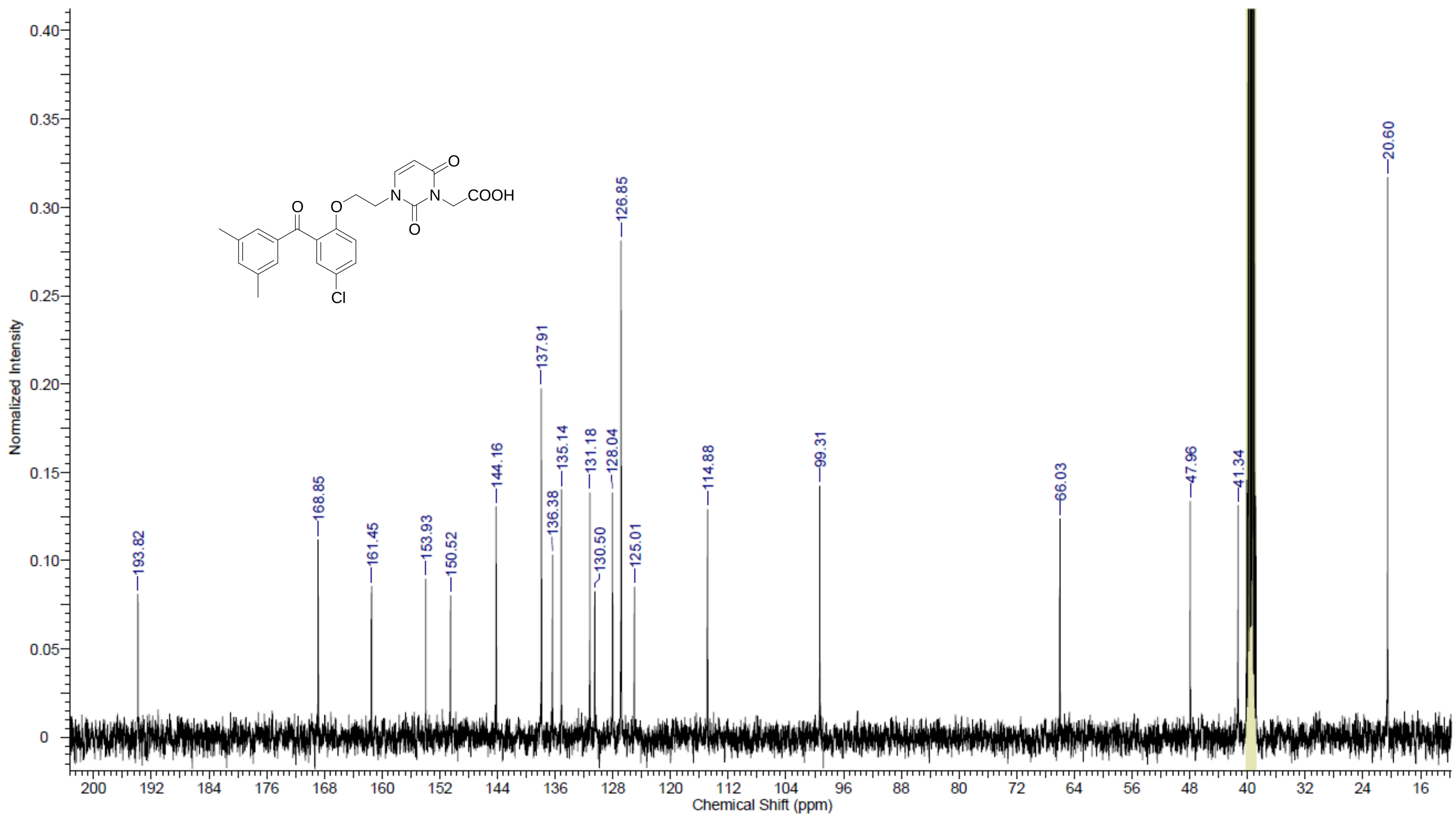

Figure S18. ^{13}C NMR spectrum of compound **5c** in DMSO-d_6 at 100 MHz.

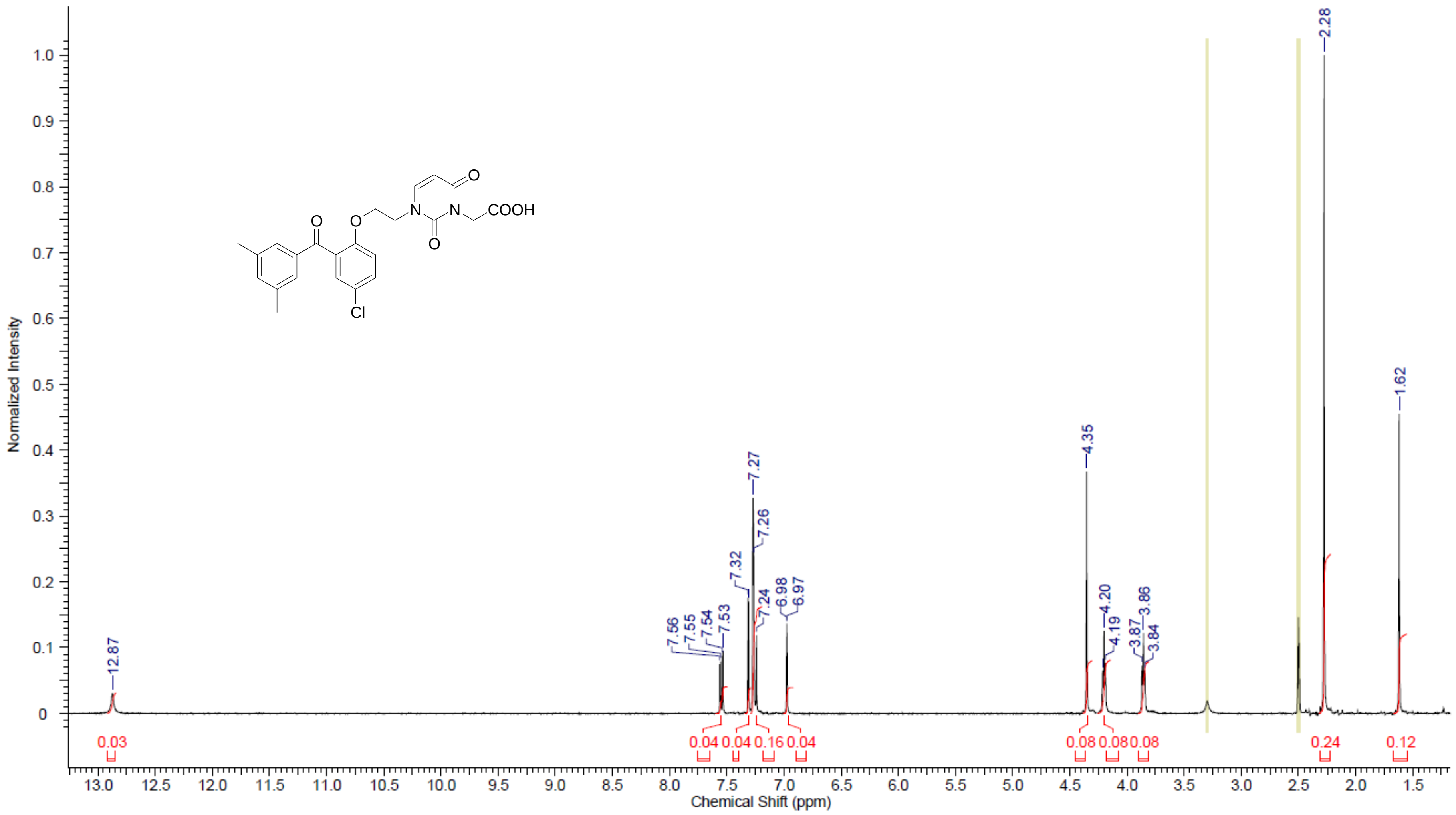

Figure S19. ^{1}H NMR spectrum of compound **5d** in DMSO-d_6 at 400 MHz.

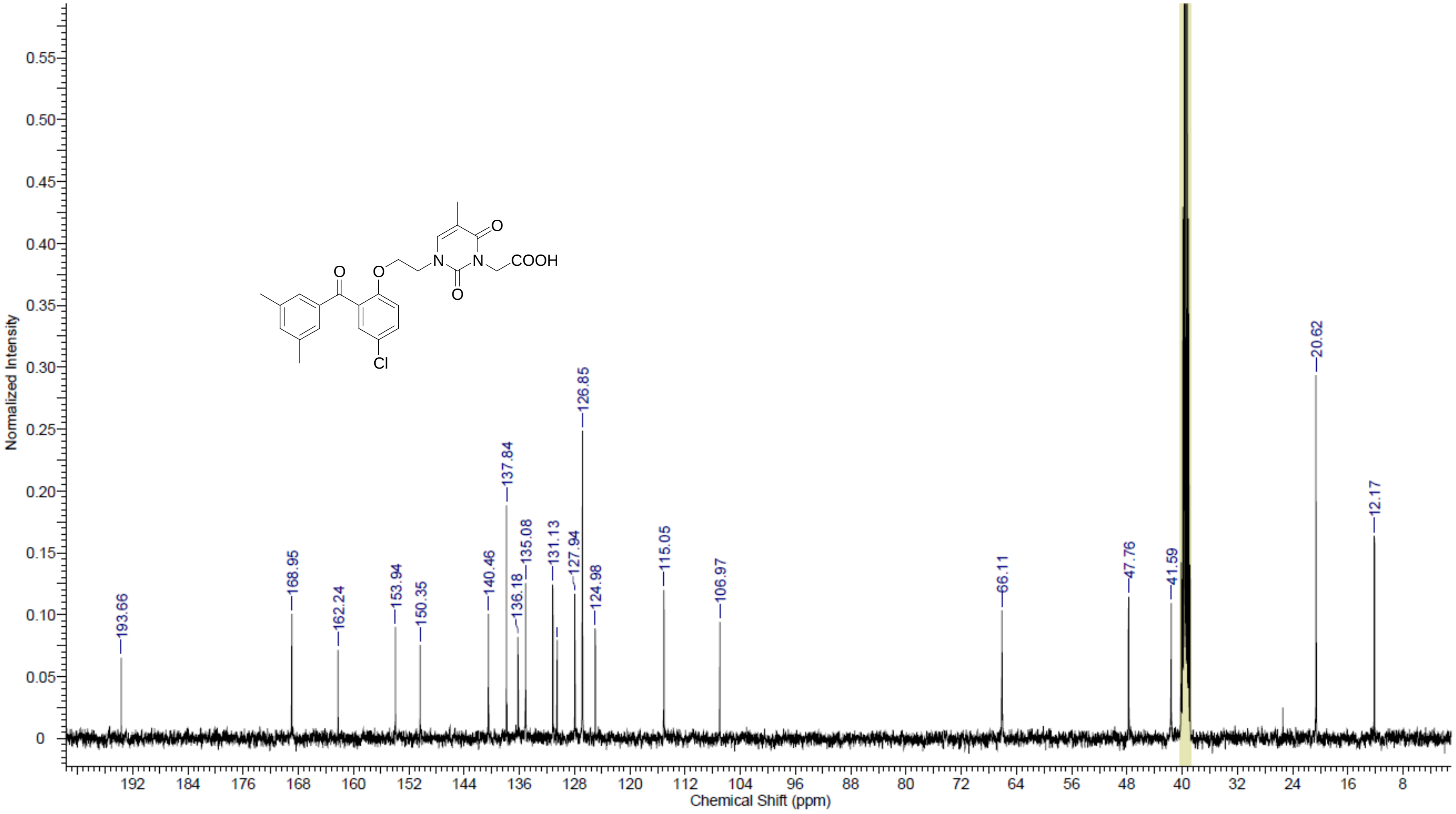

Figure S20. ^{13}C NMR spectrum of compound **5d** in DMSO-d_6 at 100 MHz.

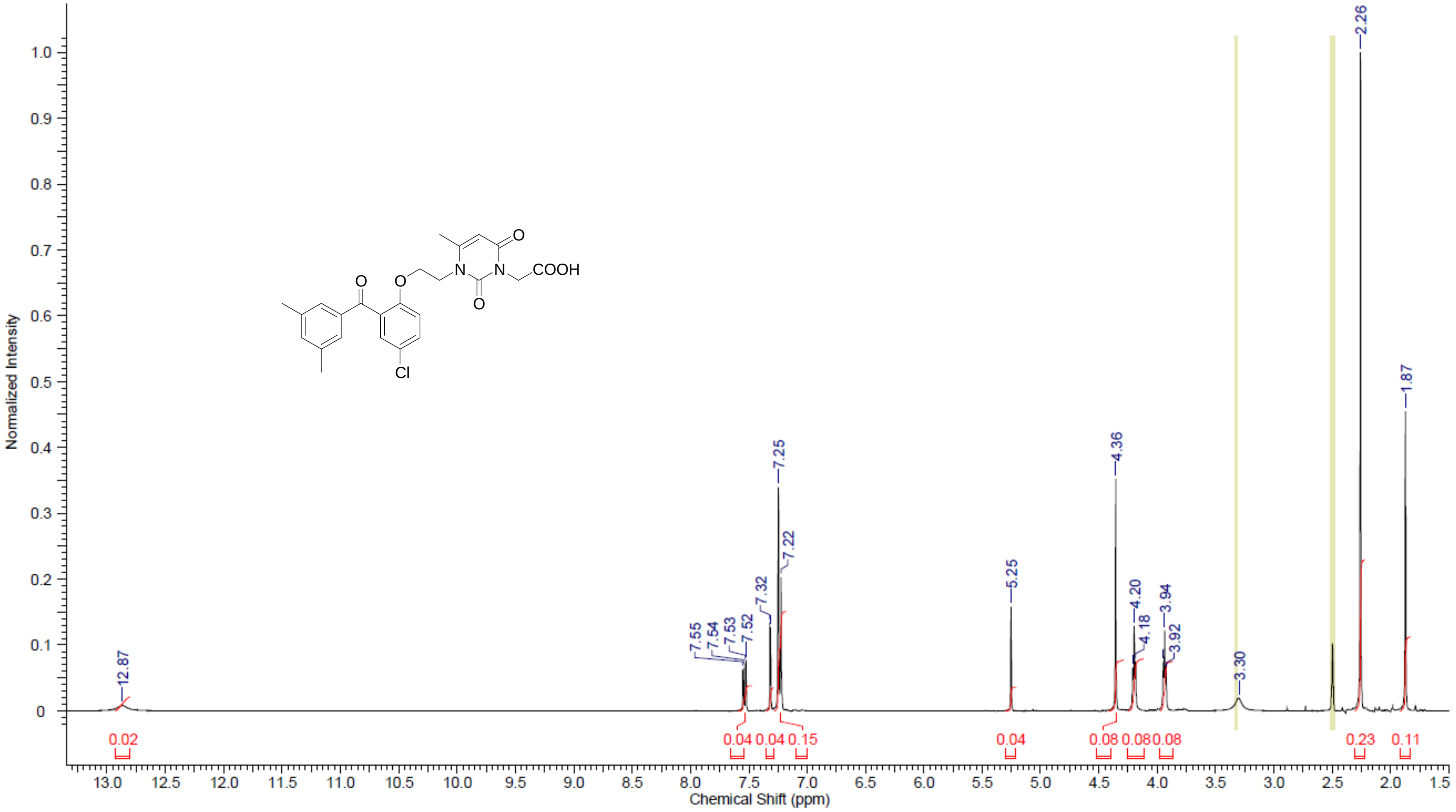

Figure S21. ^{1}H NMR spectrum of compound **5e** in DMSO-d_6 at 400 MHz.

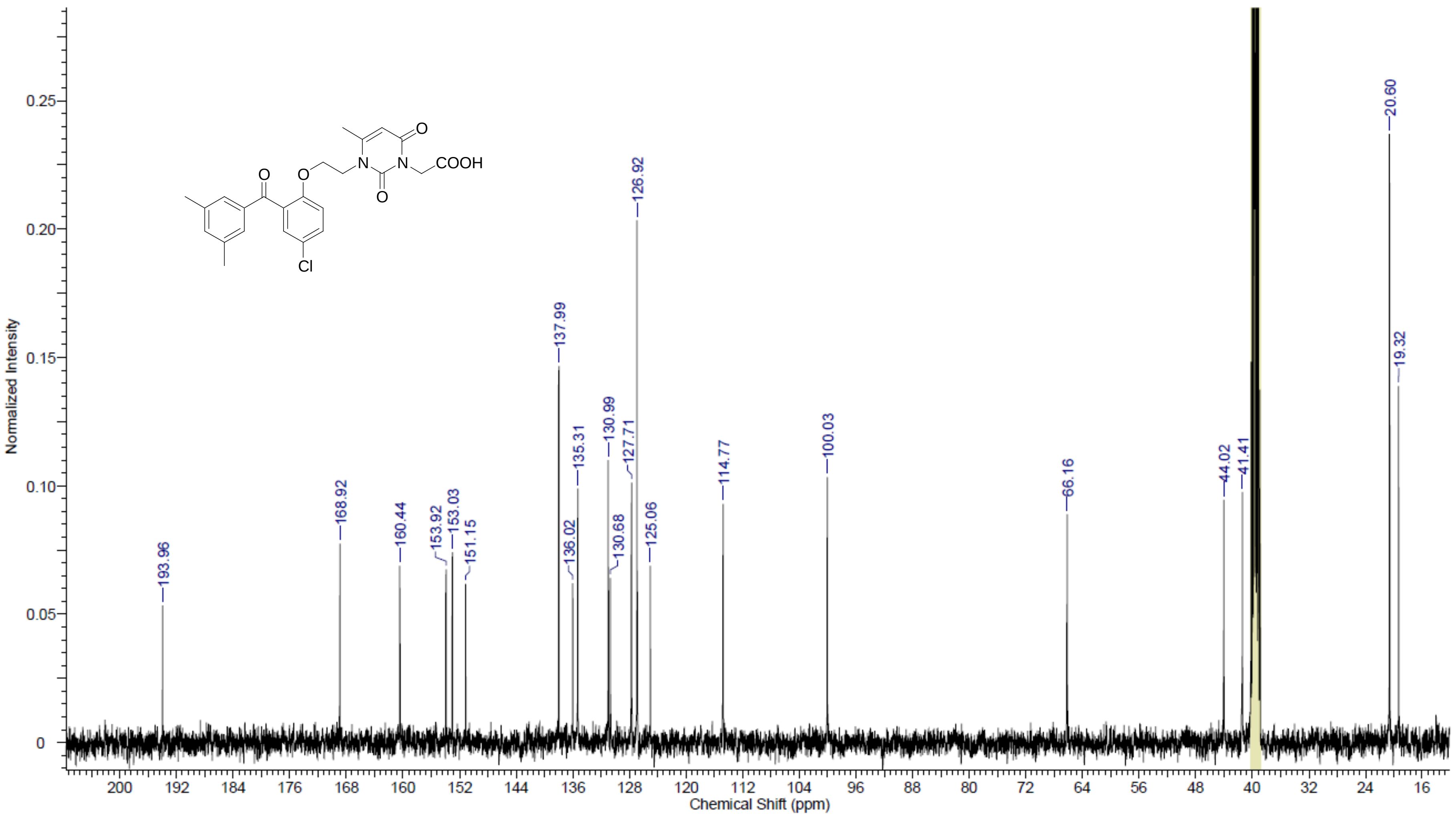

Figure S22. ^{13}C NMR spectrum of compound **5e** in DMSO-d_6 at 100 MHz.

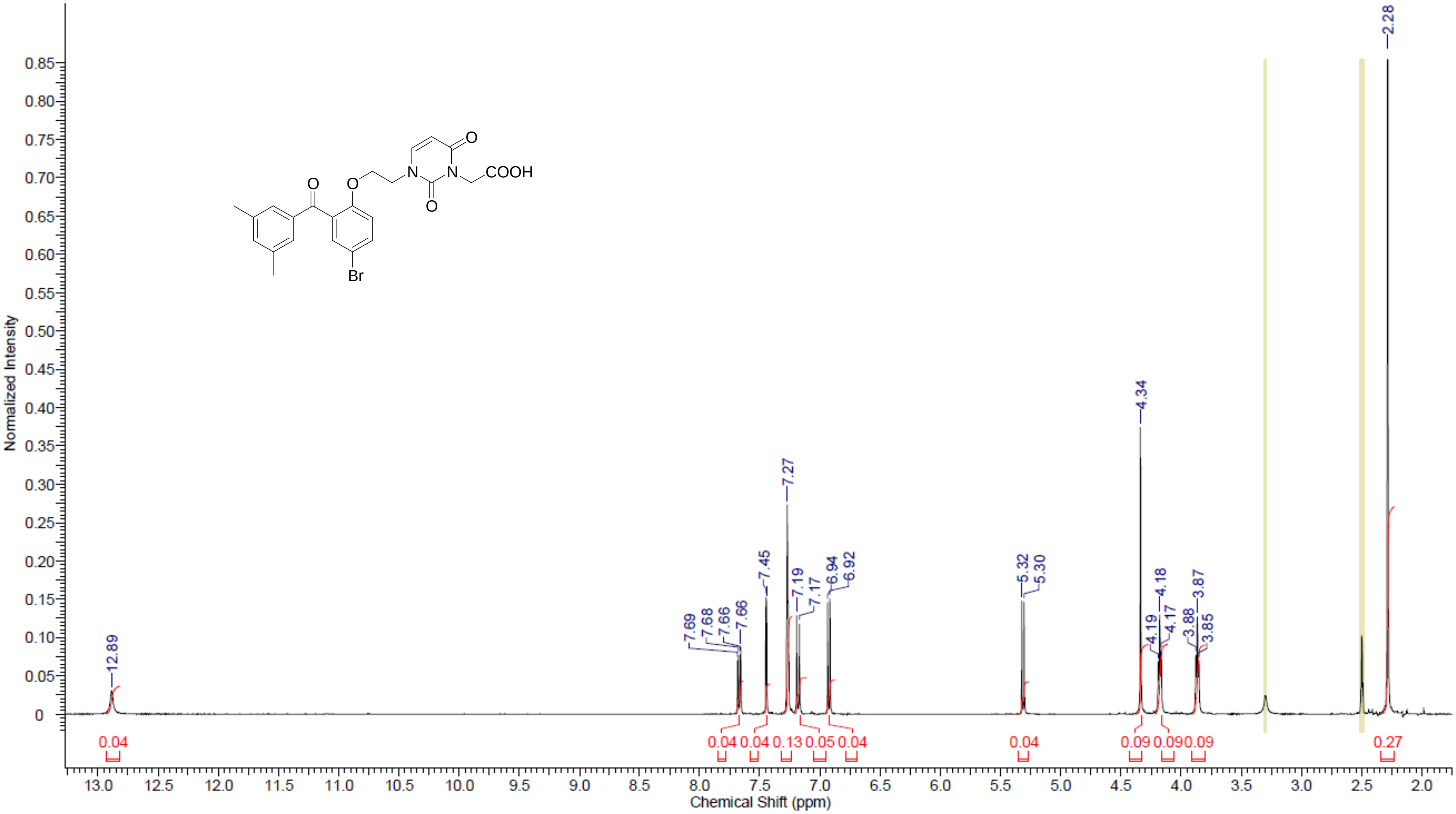

Figure S23. ^{1}H NMR spectrum of compound **5f** in DMSO-d_6 at 400 MHz.

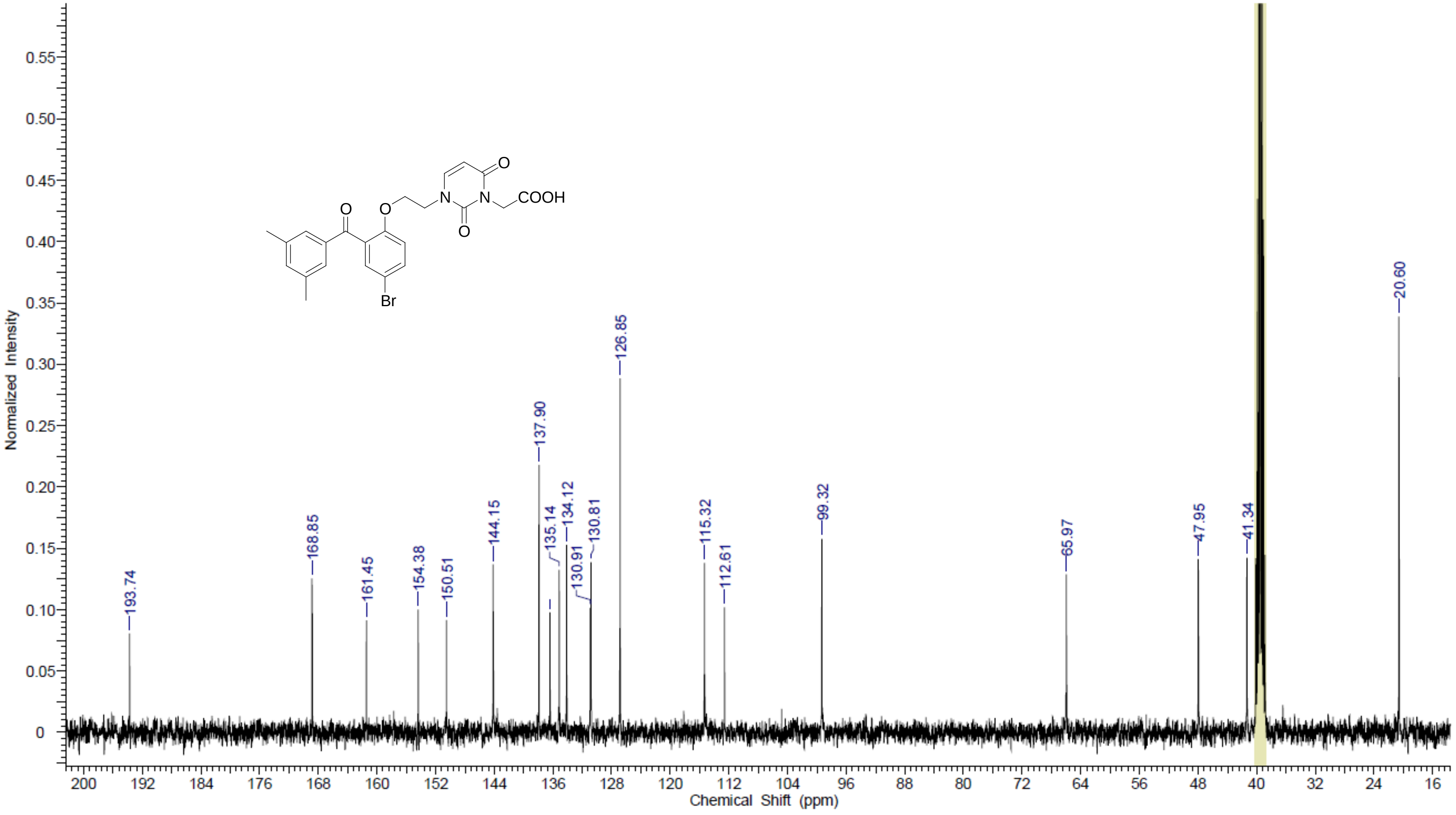

Figure S24. ^{13}C NMR spectrum of compound **5f** in DMSO-d_6 at 100 MHz.

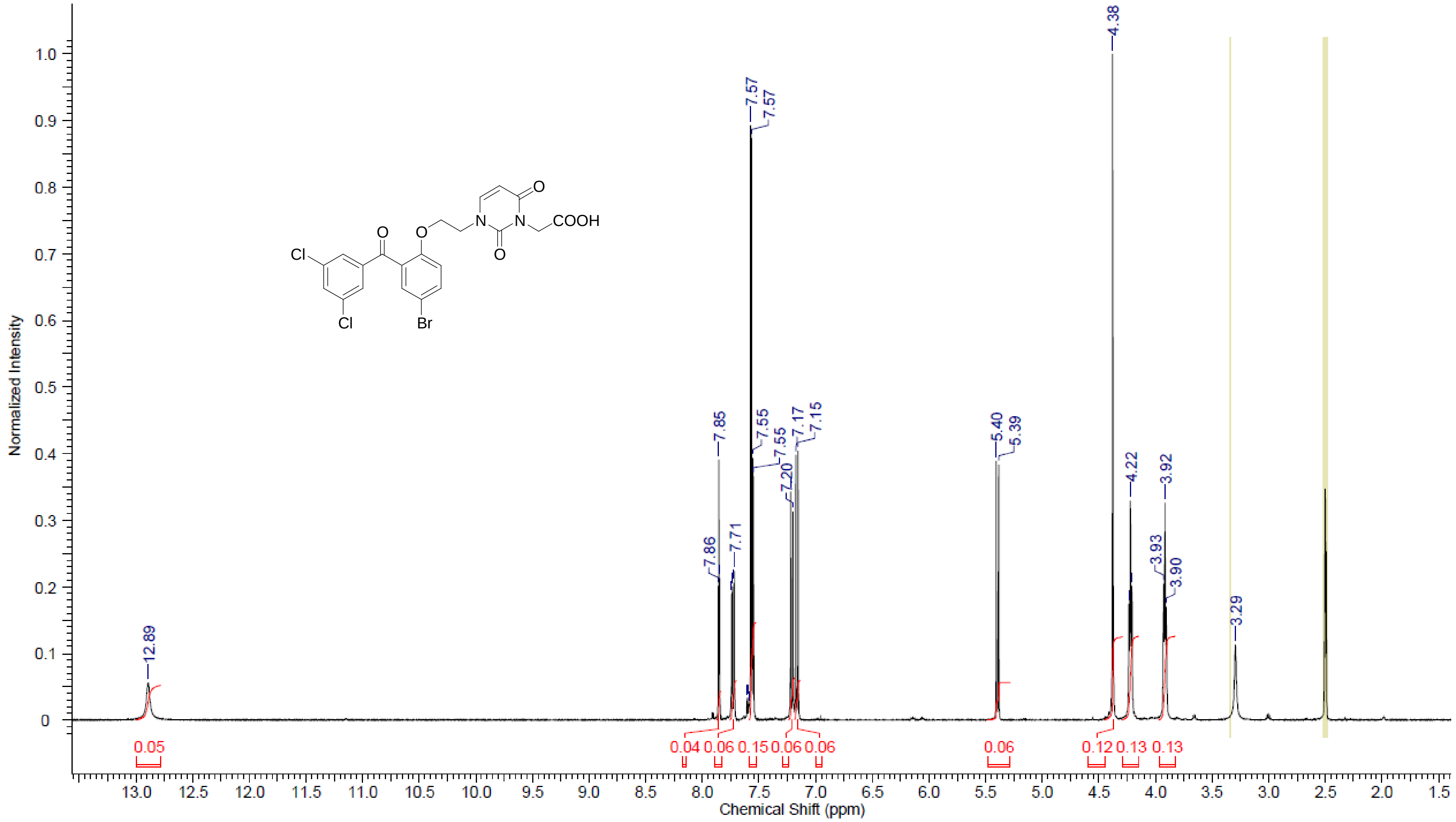

Figure S25. ^{1}H NMR spectrum of compound **5g** in DMSO-d_6 at 400 MHz.

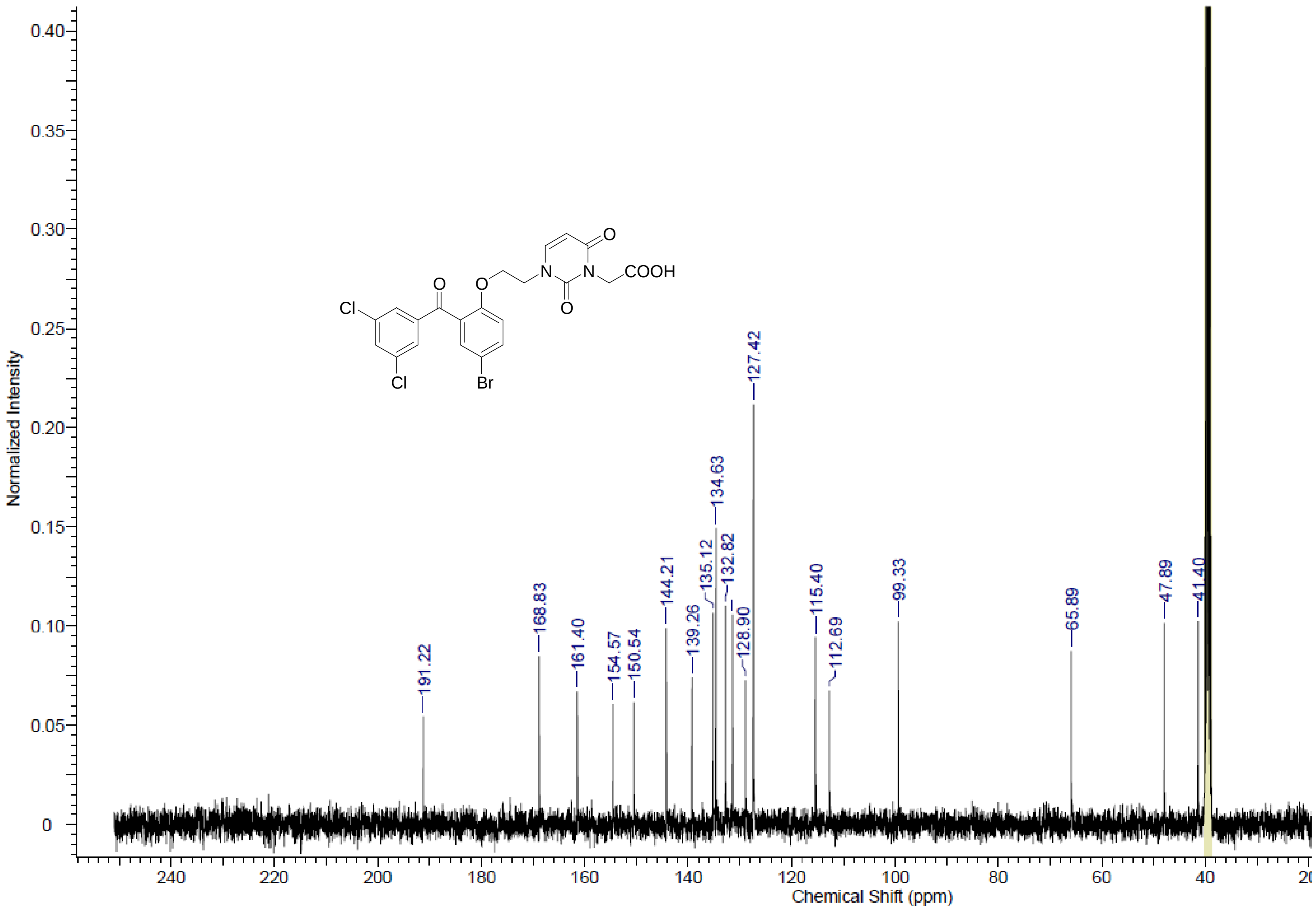

Figure S26. ^{13}C NMR spectrum of compound **5g** in DMSO-d_6 at 100 MHz.

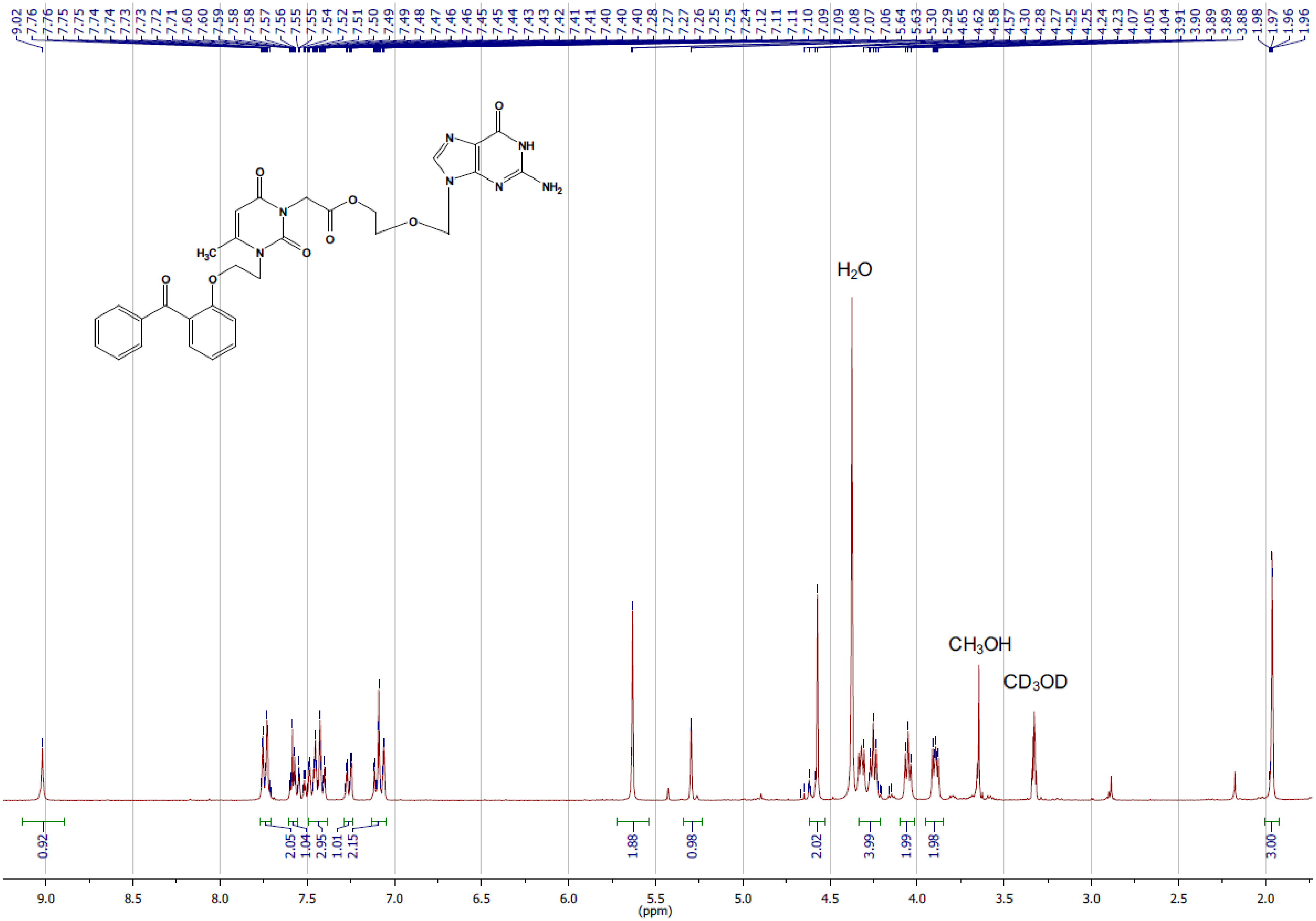

Figure S27. ^{1}H NMR spectrum of compound **2a** in CD_3OD at 400 MHz.

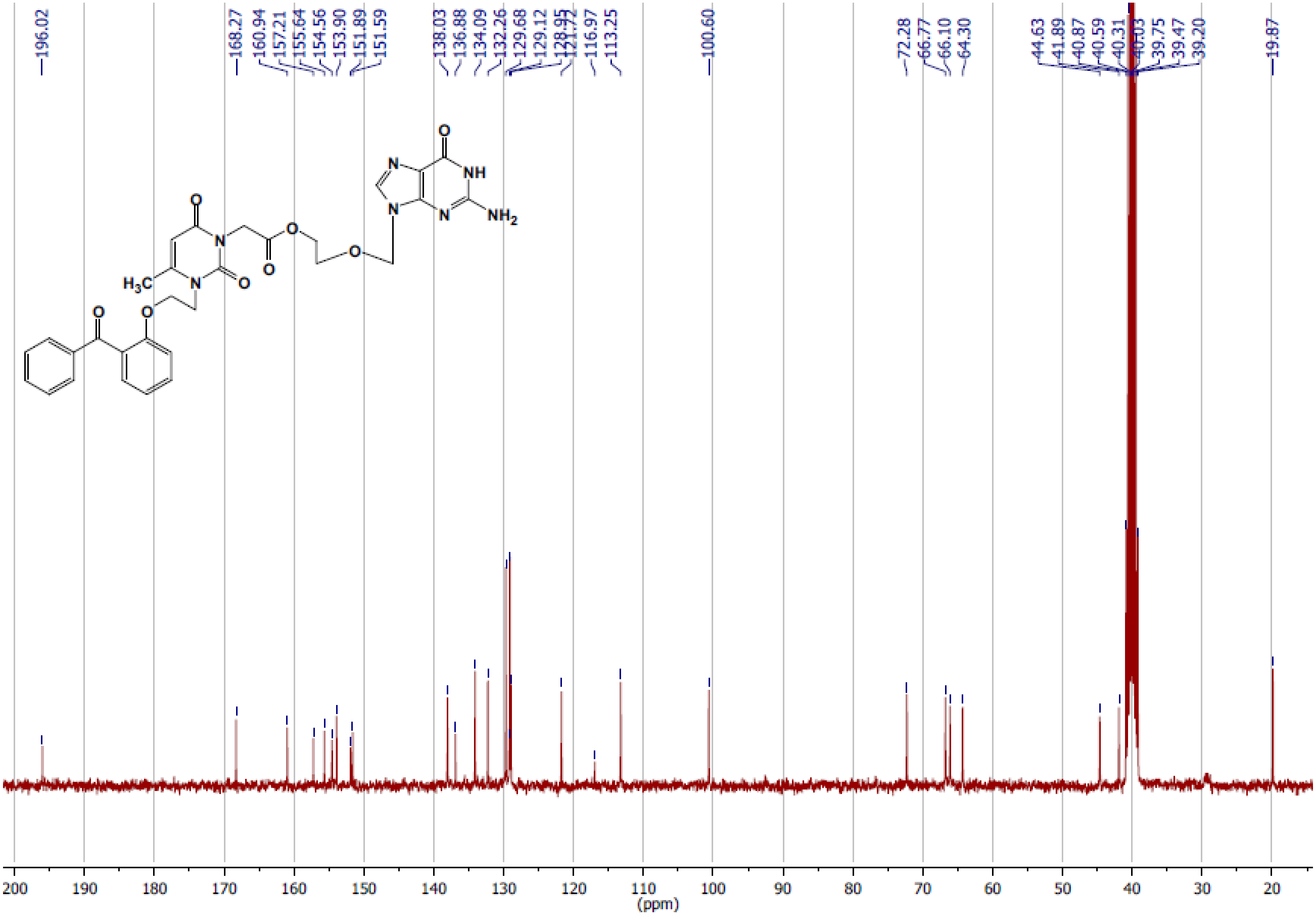

Figure S28. ^{13}C NMR spectrum of compound **2a** in DMSO-d_6 at 100 MHz.

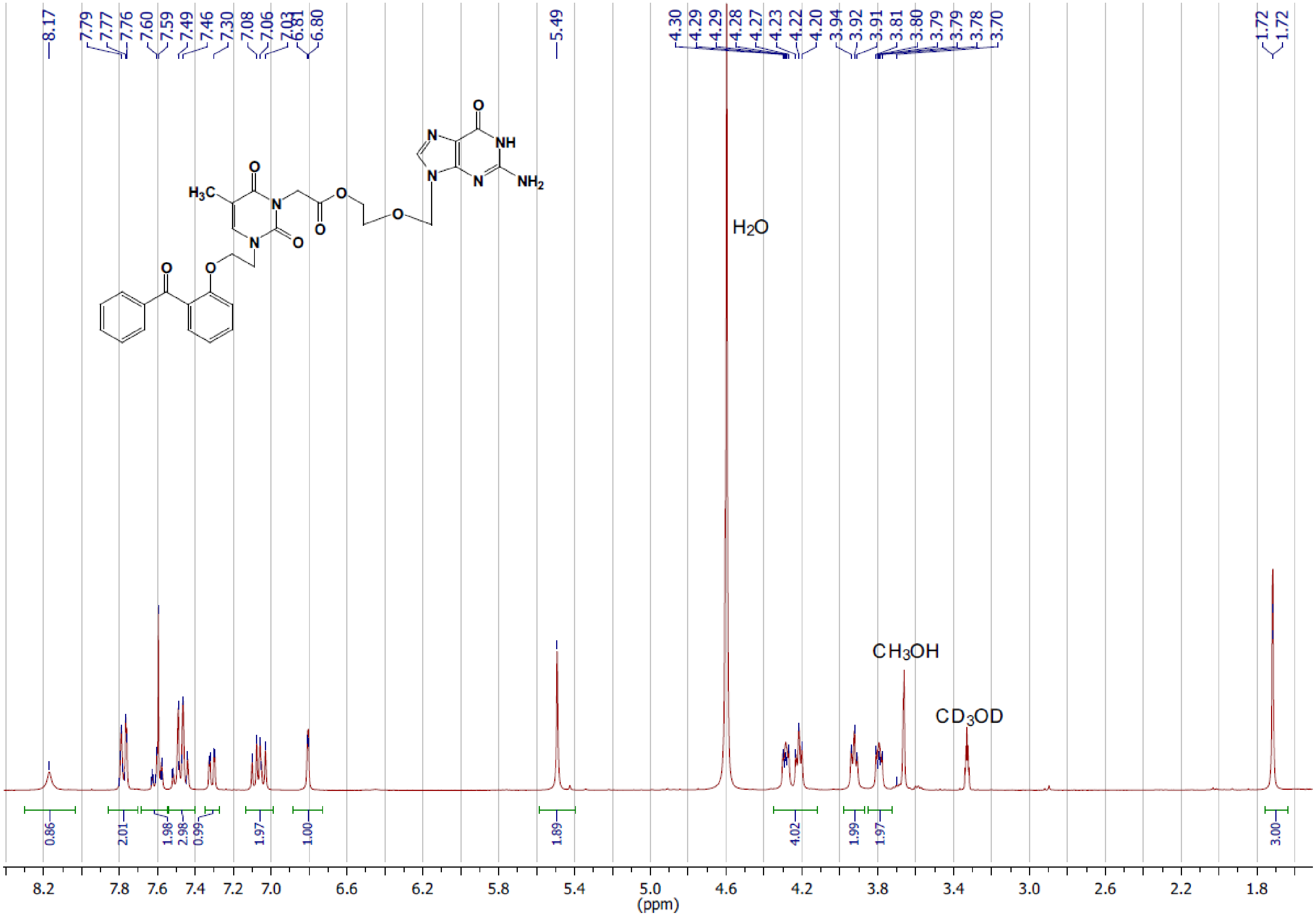

Figure S29. ^{1}H NMR spectrum of compound **2b** in CD_3OD at 400 MHz.

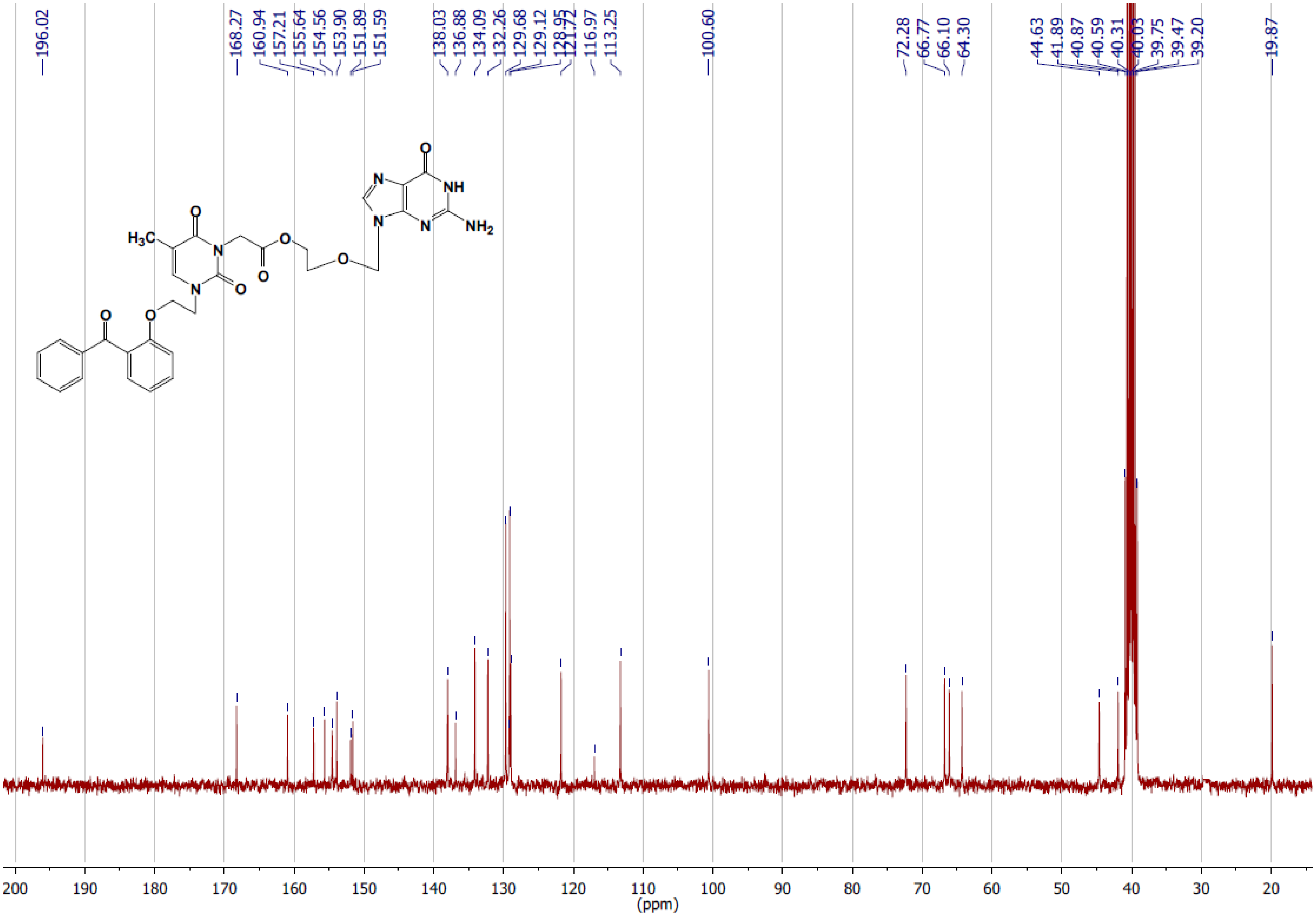

Figure S30. ^{13}C NMR spectrum of compound **2b** in DMSO-d_6 at 100 MHz.

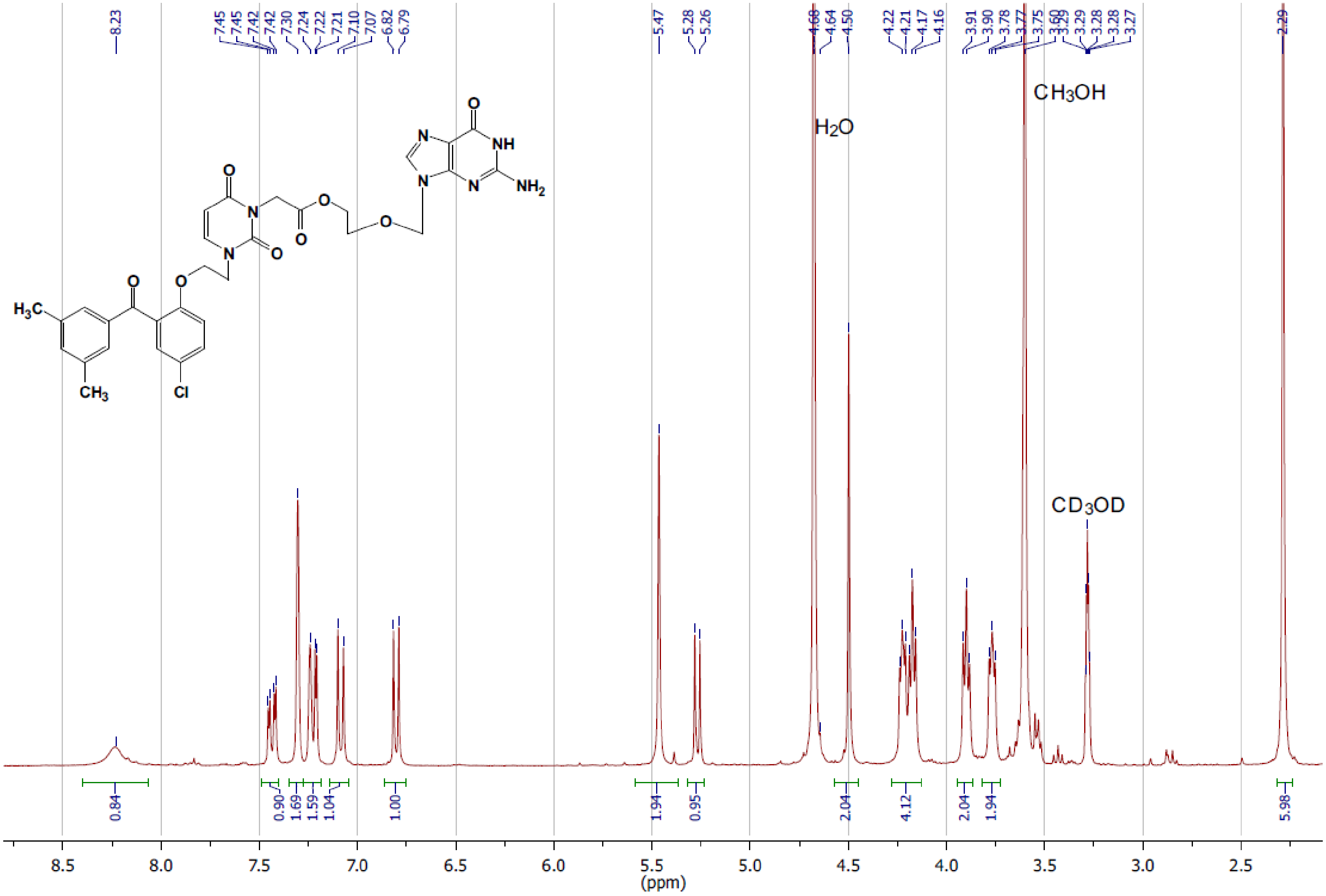

Figure S31. ^{1}H NMR spectrum of compound **2c** in CD_3OD at 400 MHz.

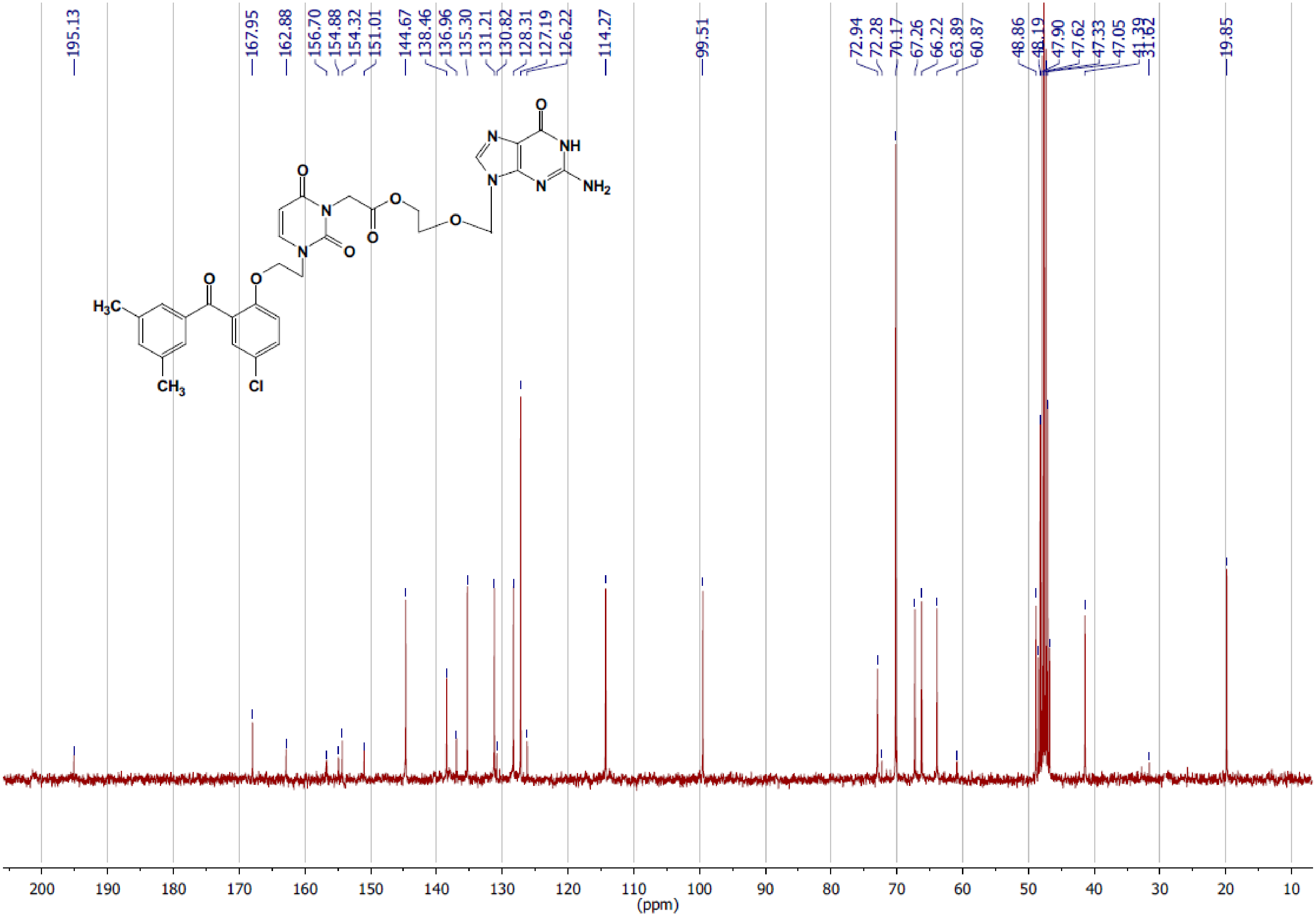

Figure S32. ^{13}C NMR spectrum of compound **2c** in CD_3OD at 100 MHz.

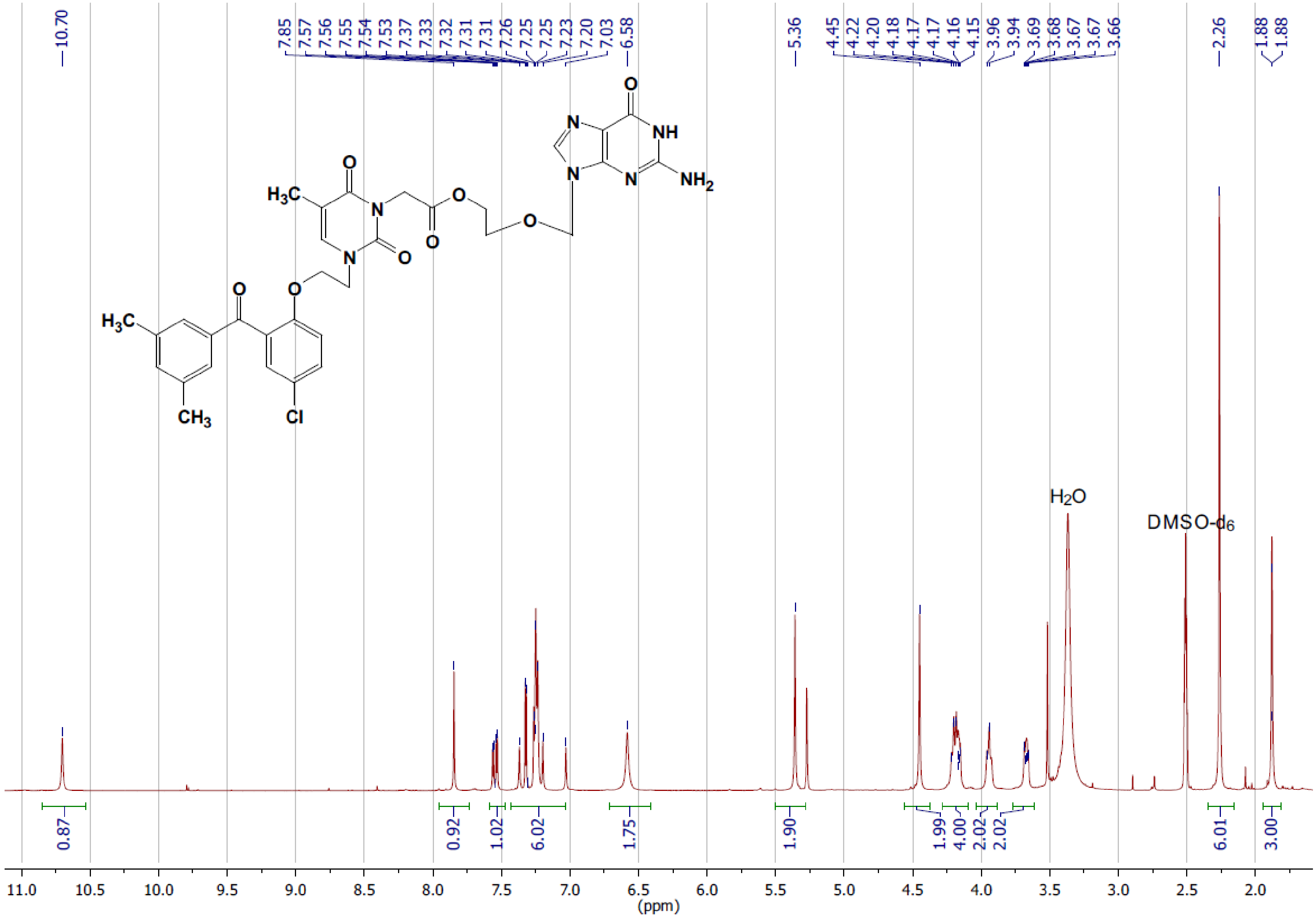

Figure S33. ^{1}H NMR spectrum of compound **2d** in DMSO-d6 at 400 MHz.

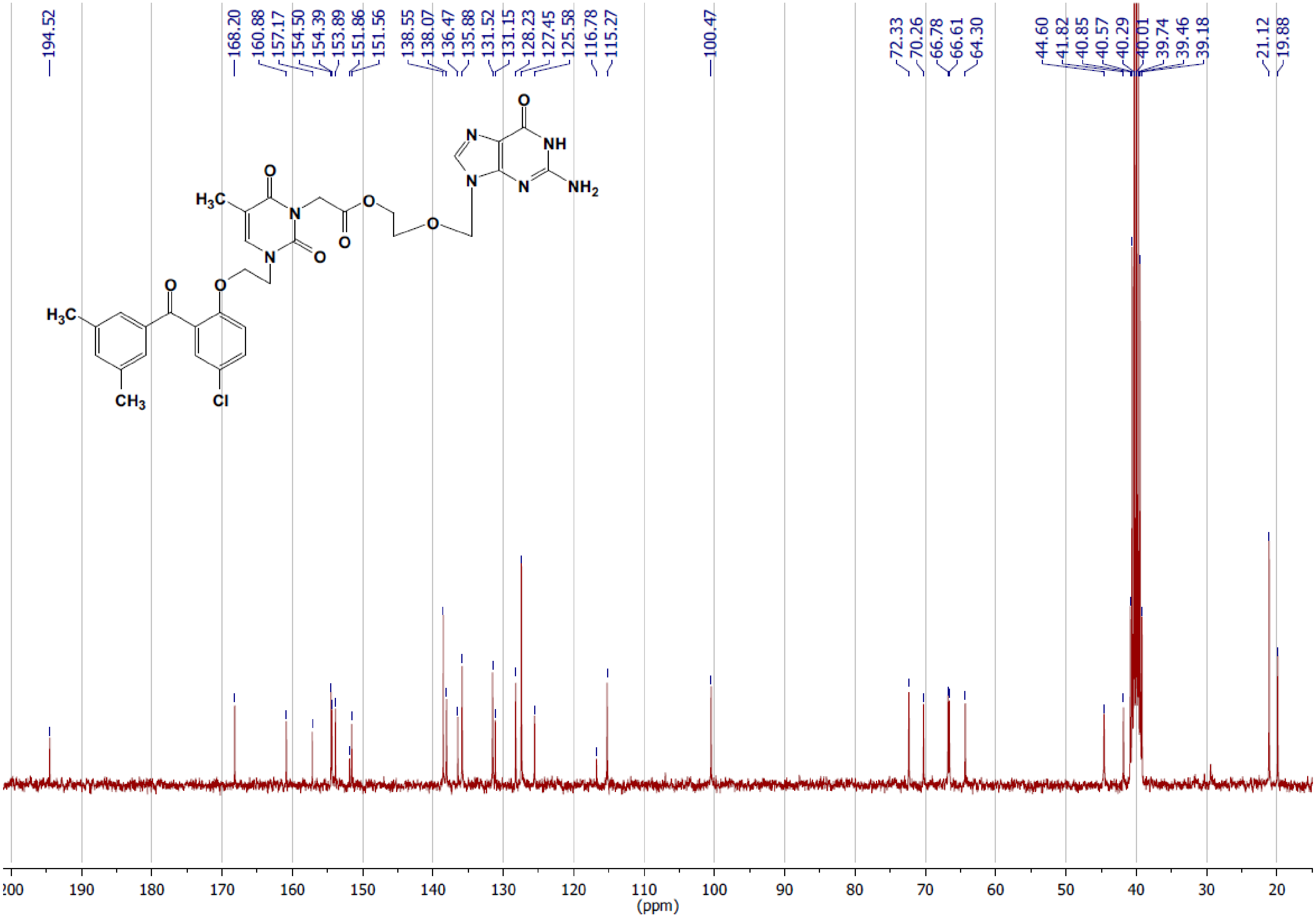

Figure S34. ^{13}C NMR spectrum of compound **2d** in DMSO-d_6 at 100 MHz.

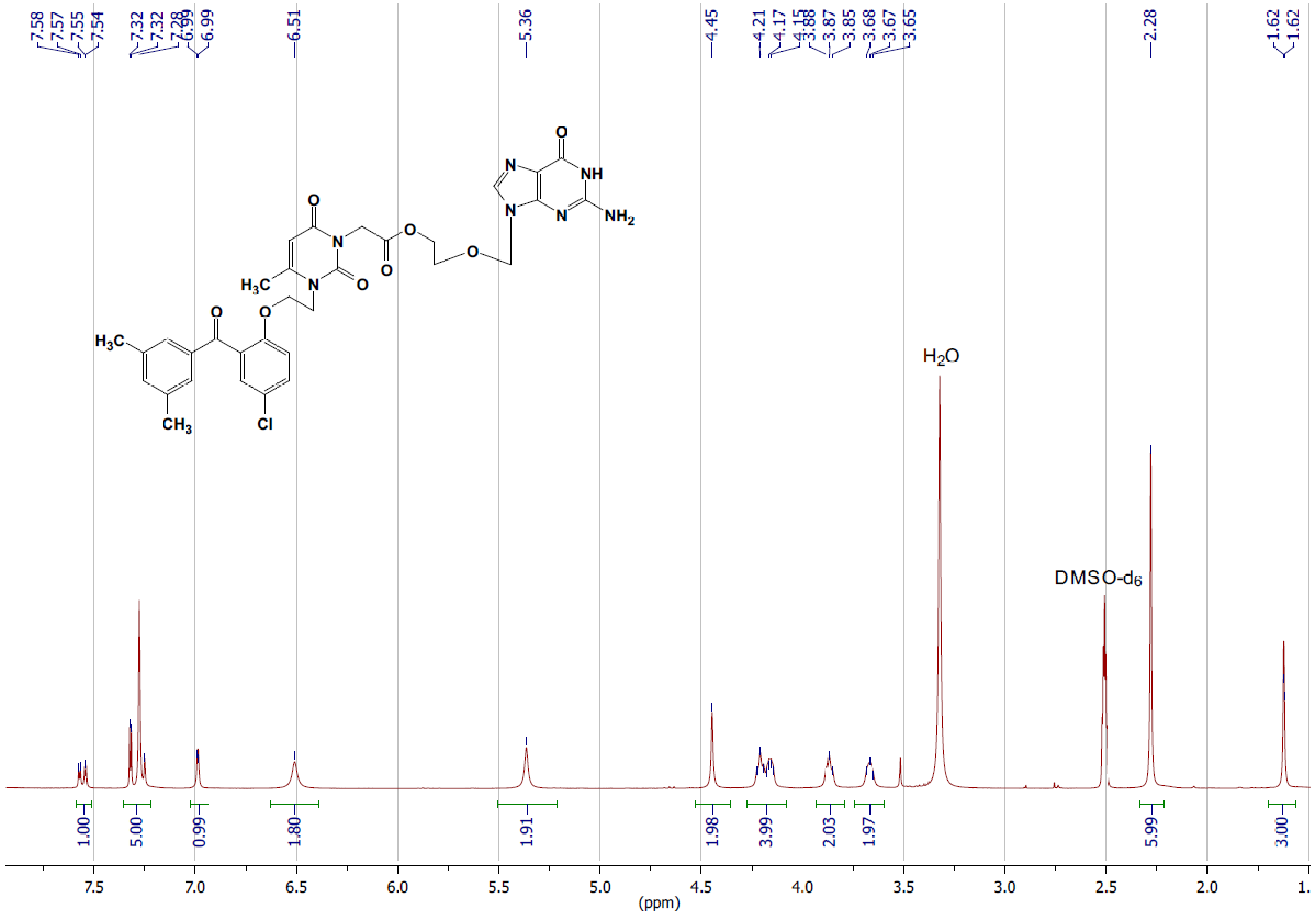
7.58
7.57
7.55
7.54
7.32
7.32
7.28
6.99
6.99
6.51
5.36
4.45
4.21
4.17
4.15
3.88
3.87
3.85
3.68
3.67
3.65
2.28
1.62
1.62
H2O
DMSO-d6
1.00
5.00
0.99
1.80
1.91
1.98
3.99
2.03
1.97
5.99
3.00
7.5
7.0
6.5
6.0
5.5
5.0
4.5
4.0
3.5
3.0
2.5
2.0
1.
(ppm)
O
N
NH
N
N
NH2
O
N
O
O
O
H3C
N
O
O
O
H3C
CH3
Cl

Figure S35.^{1}H NMR spectrum of compound **2e** in DMSO-d6 at 400 MHz.

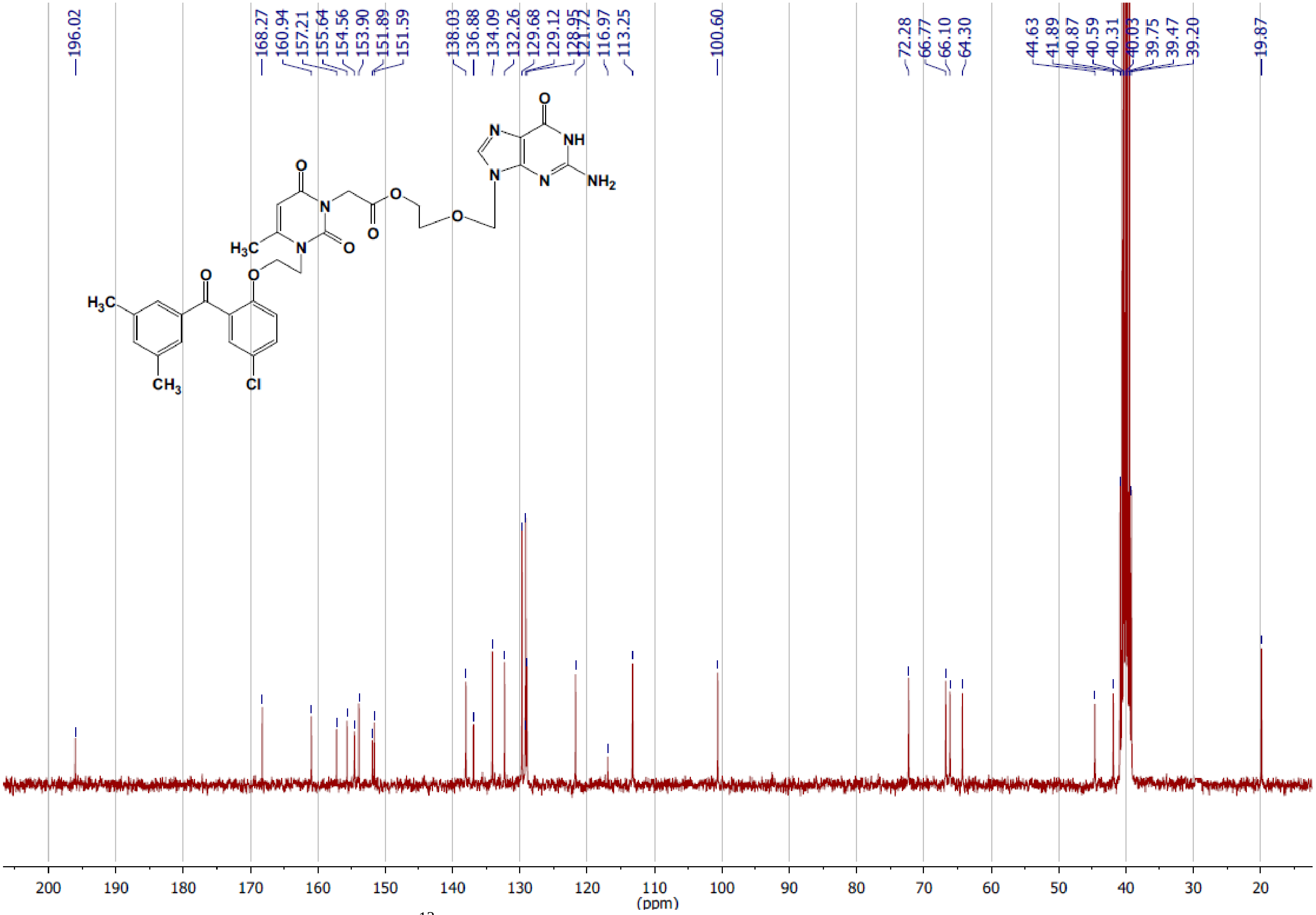

Figure S36. ^{13}C NMR spectrum of compound **2e** in DMSO-d_6 at 100 MHz.

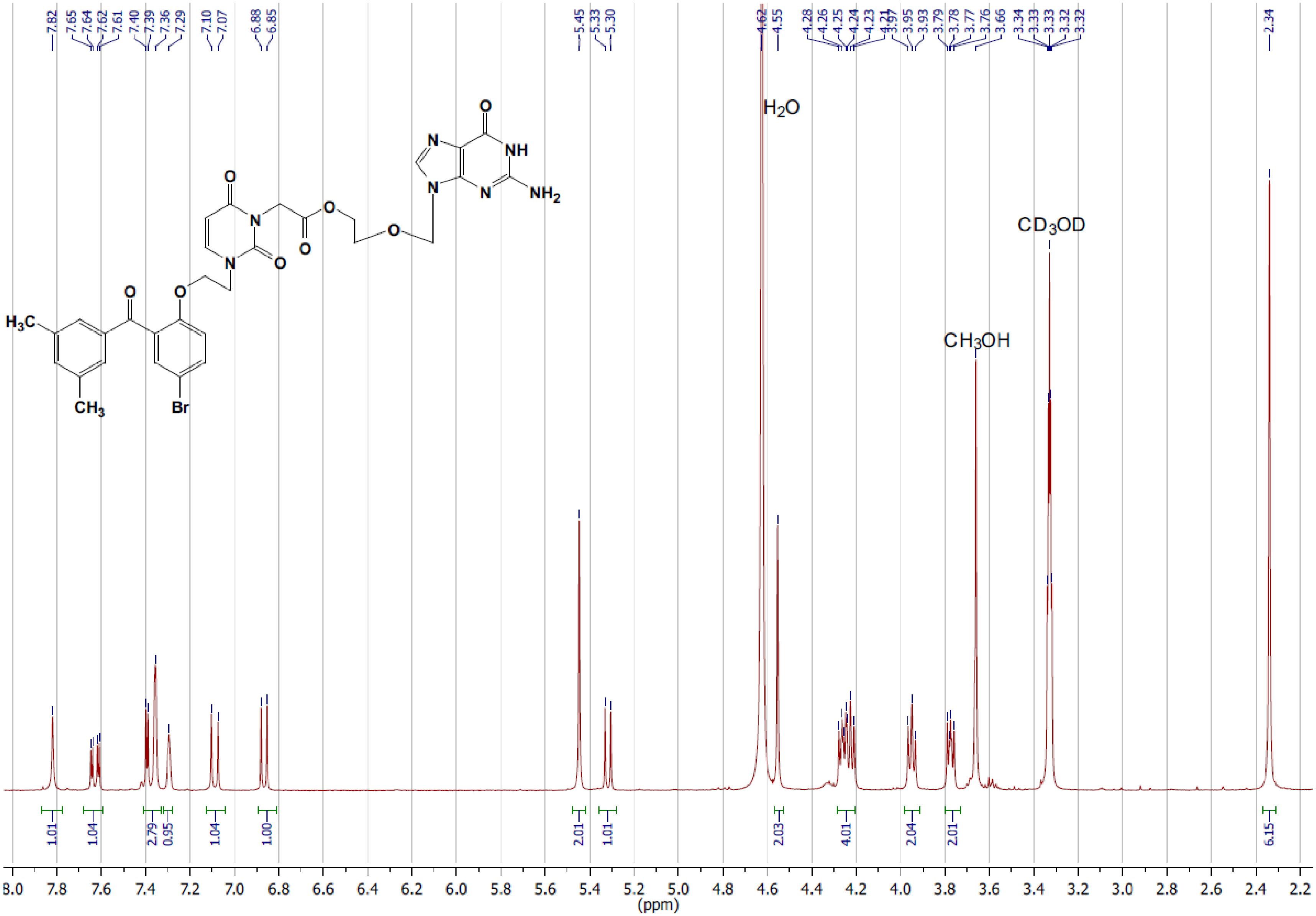

Figure S37.^{1}H NMR spectrum of compound **2f** in CD_3OD at 400 MHz.

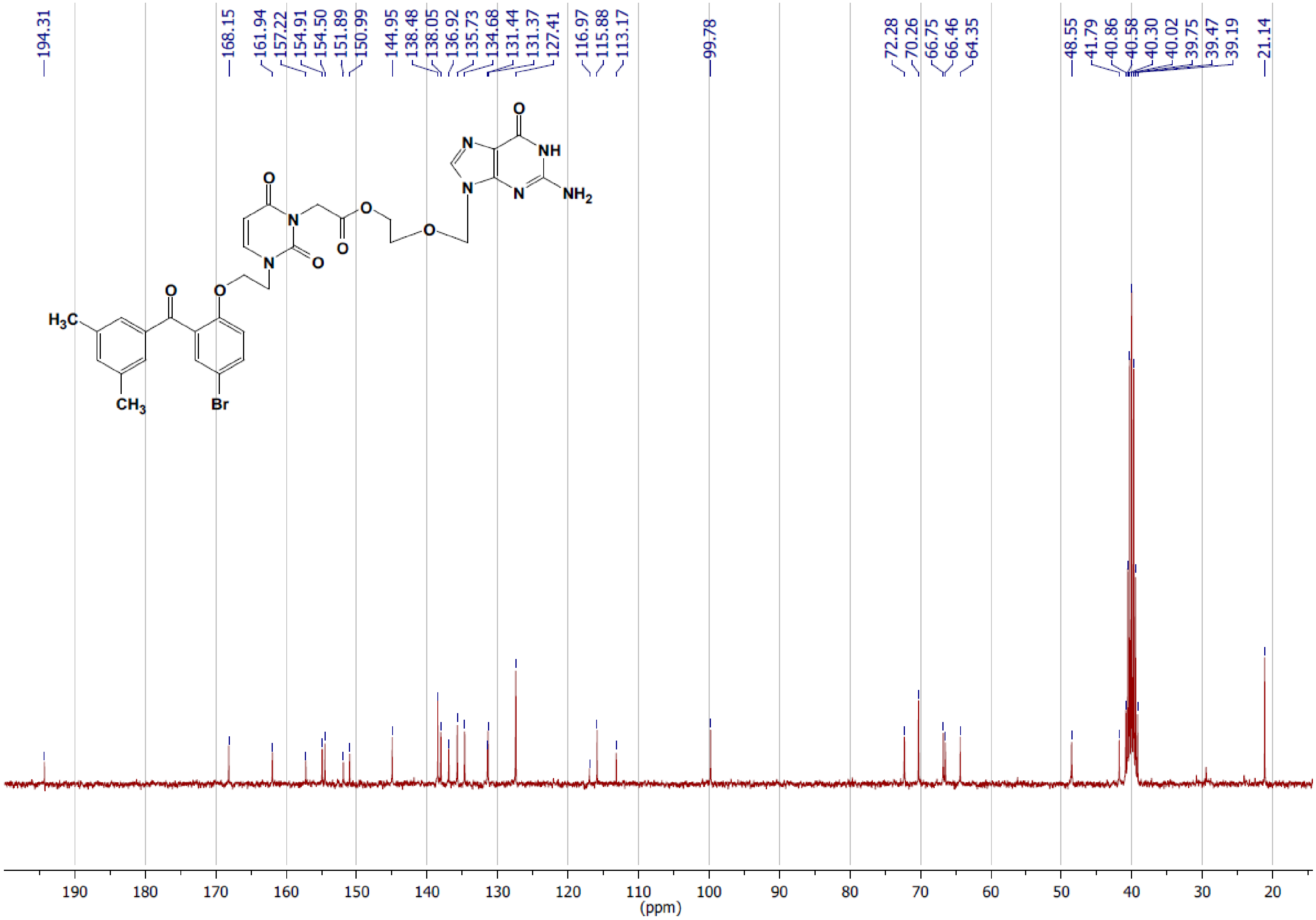

Figure S38. ^{13}C NMR spectrum of compound **2f** in DMSO-d_6 at 100 MHz.

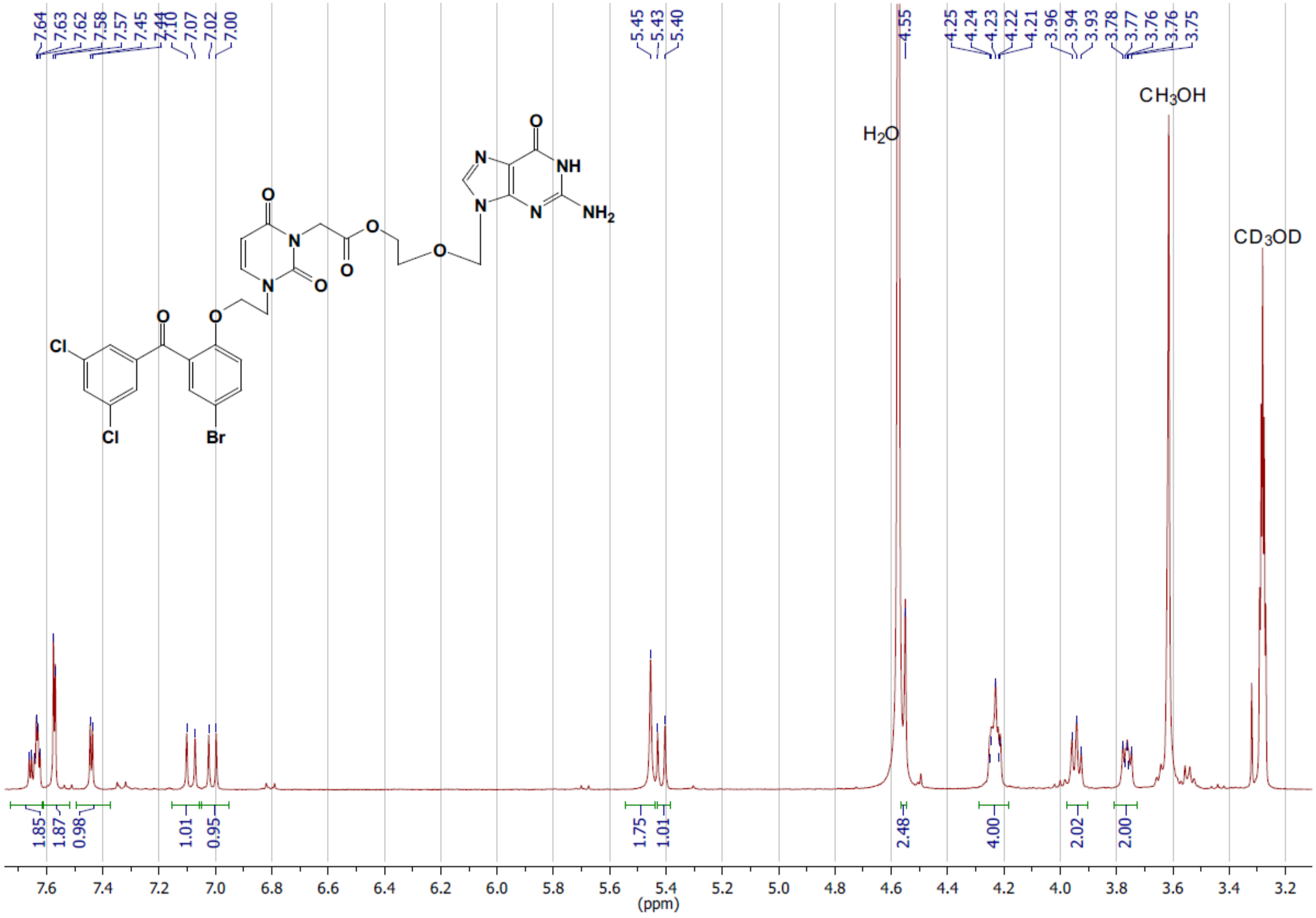

Figure S39.^{1}H NMR spectrum of compound **2g** in CD_3OD at 400 MHz.

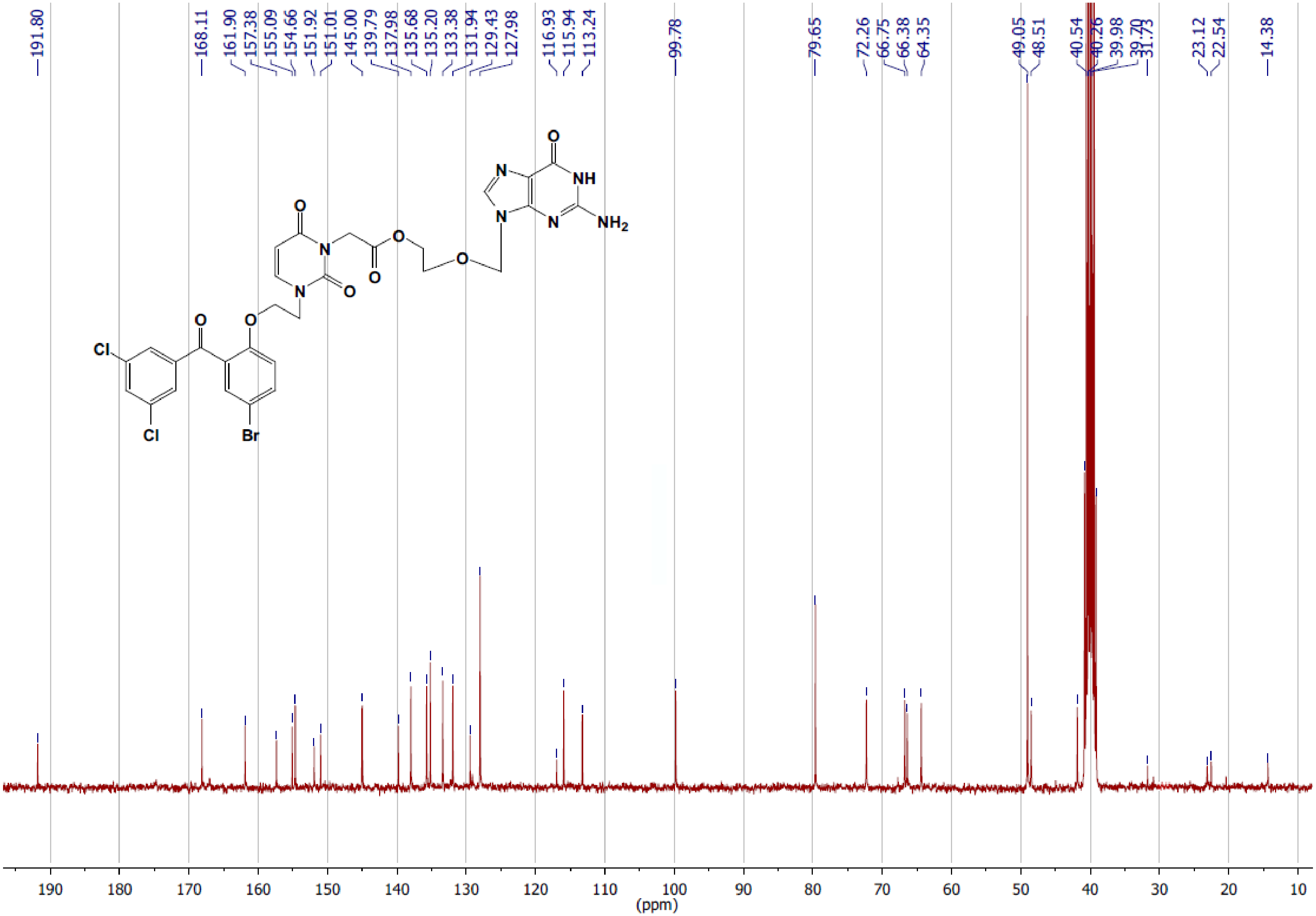

Figure S40. ^{13}C NMR spectrum of compound **2g** in DMSO-d_6 at 100 MHz.

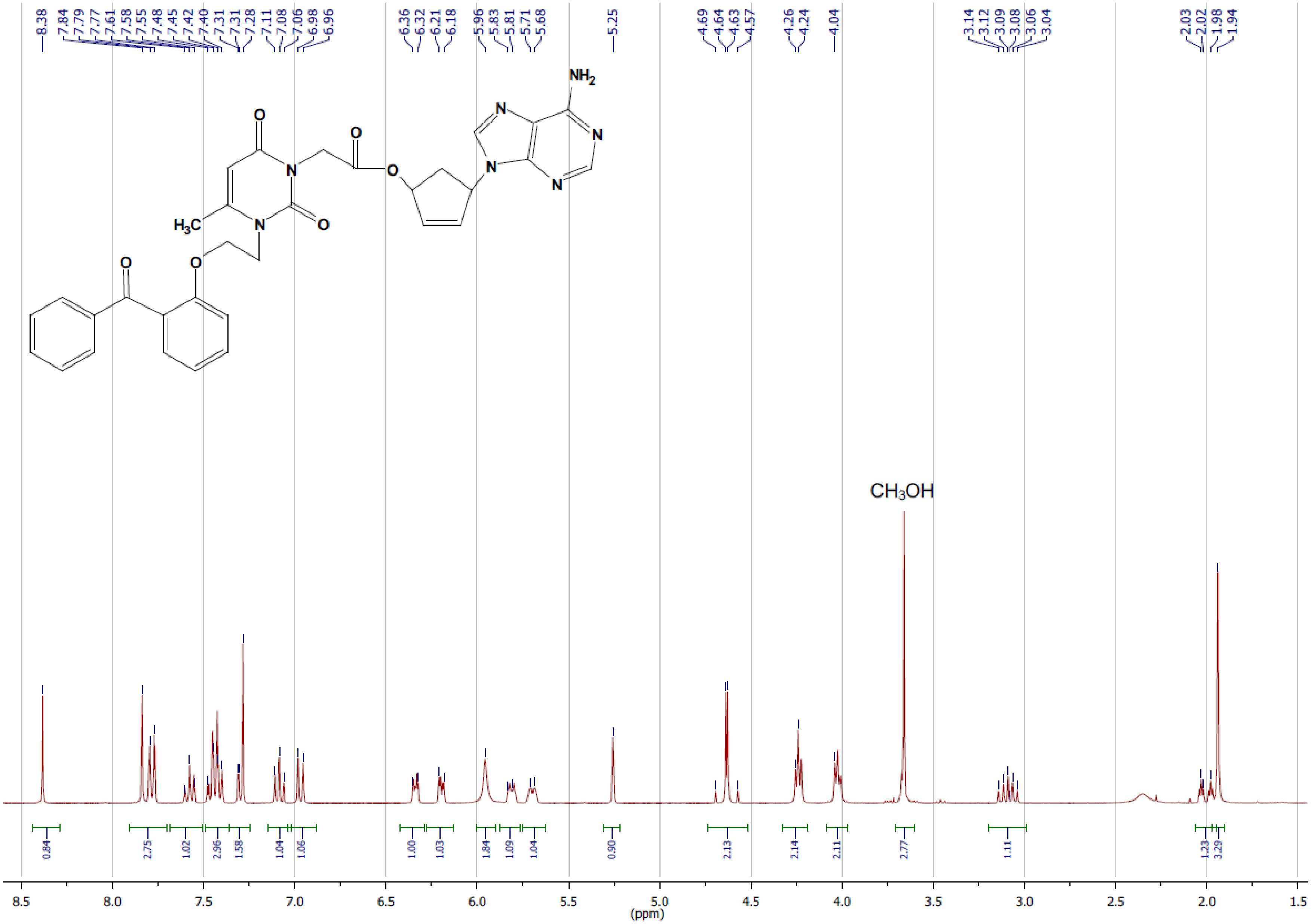

Figure S41. ^{1}H NMR spectrum of compound **6a** (in $CDCl_3$ at 400 MHz)

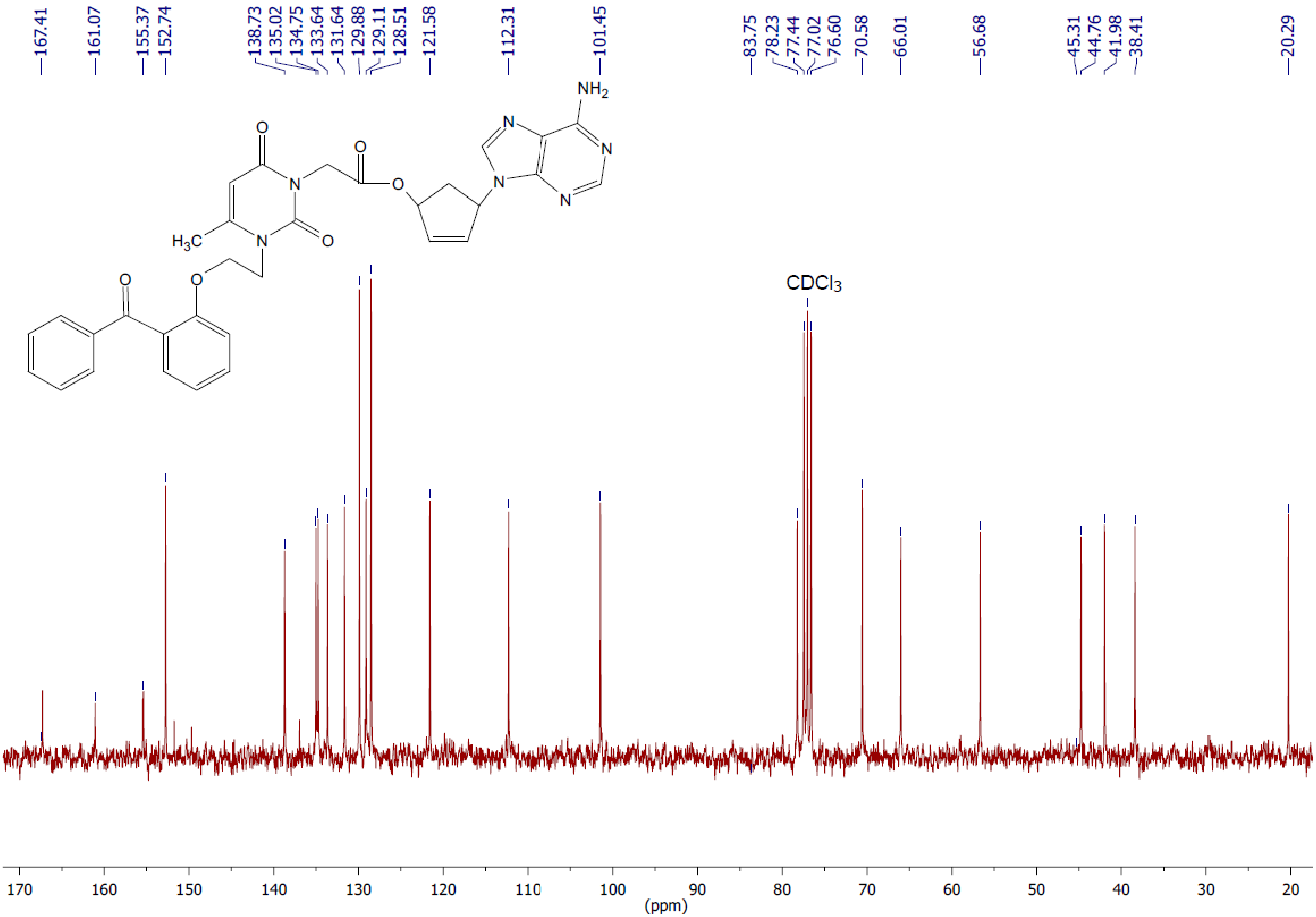

Figure S42. ^{13}C NMR spectrum of compound **6a** (in $CDCl_3$ at 100 MHz)

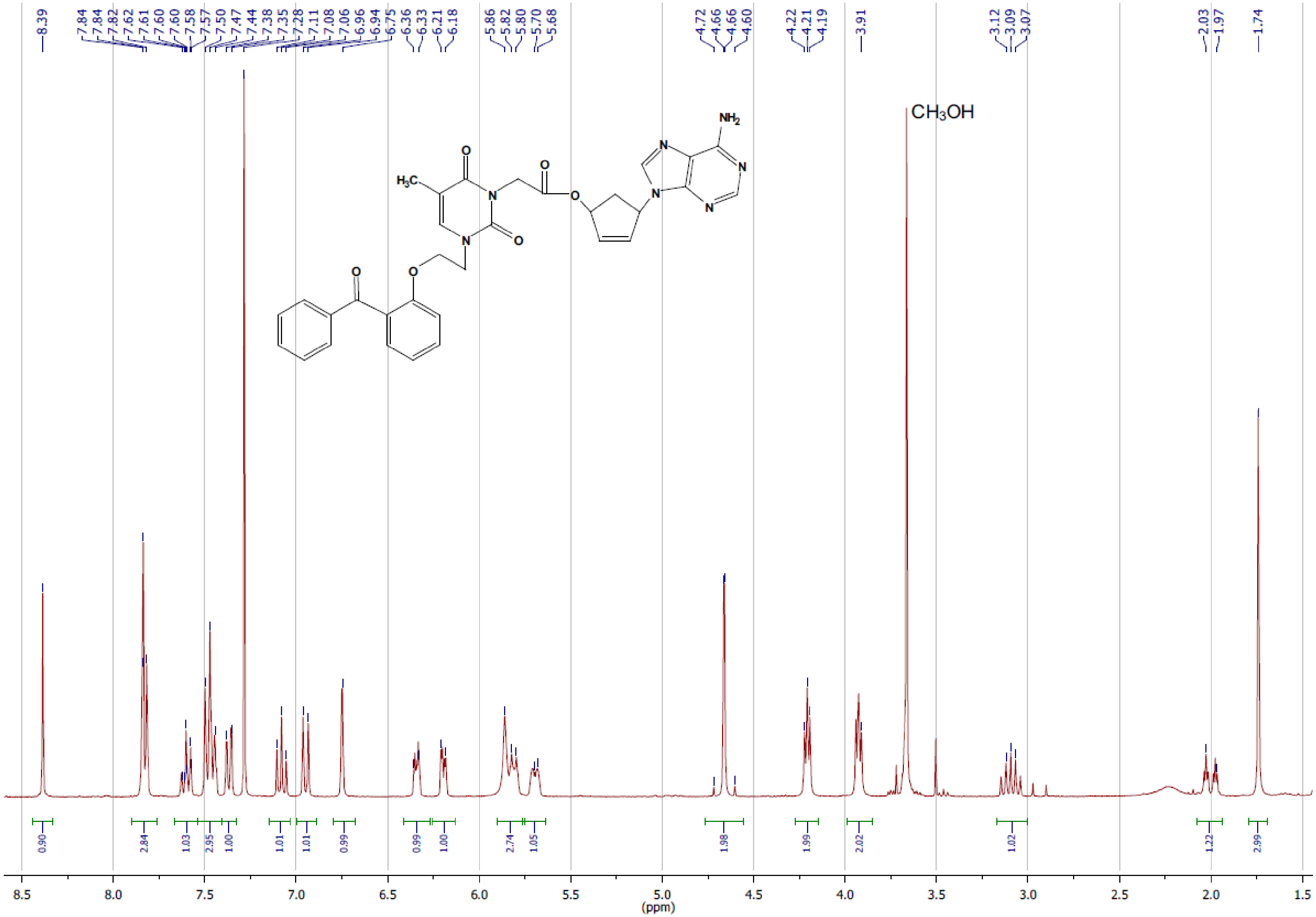

Figure S43. ^{1}H NMR spectrum of compound **6b** (in $CDCl_3$ at 400 MHz)

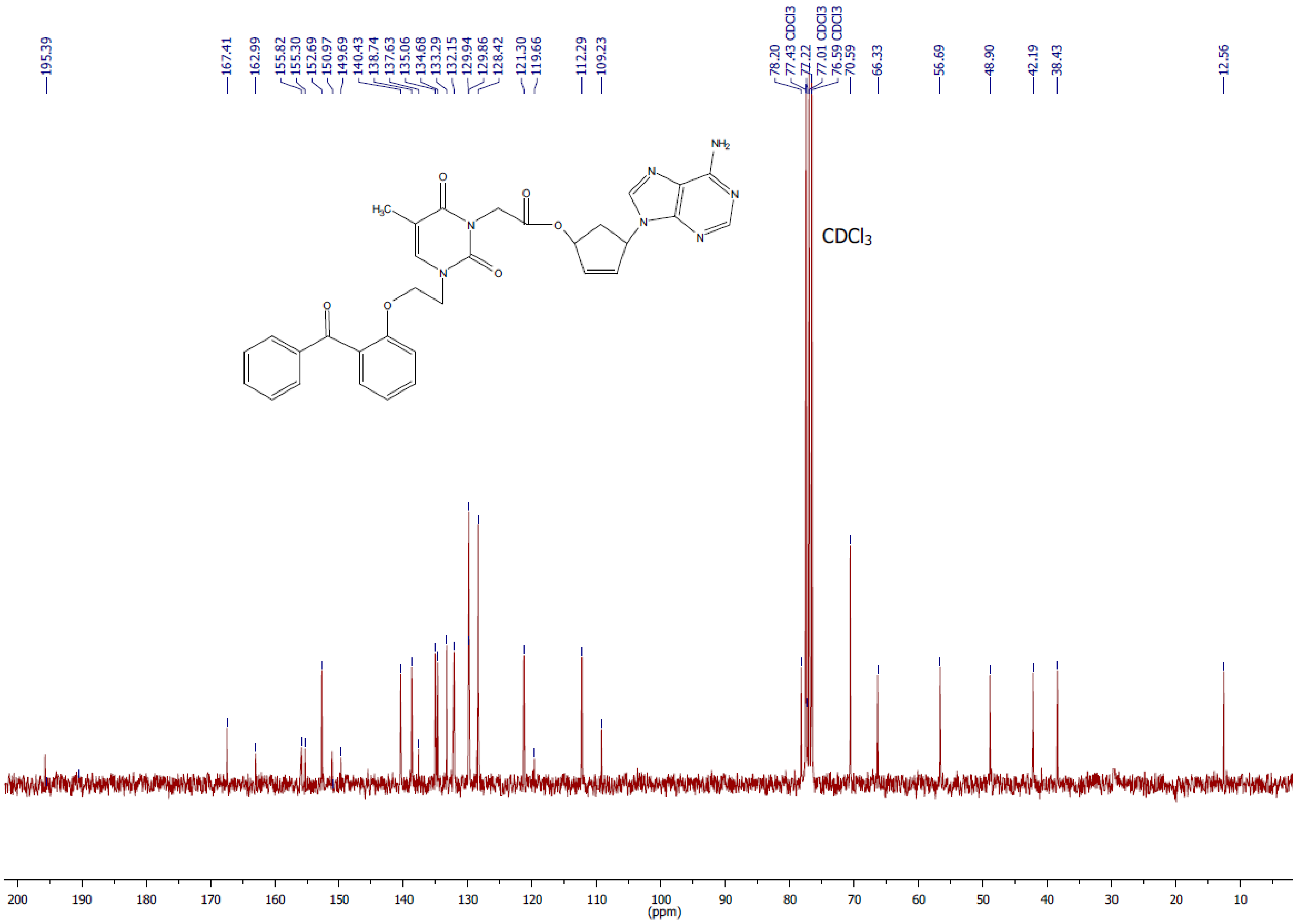

Figure S44. ^{13}C NMR spectrum of compound **6b** (in $CDCl_3$ at 100 MHz)

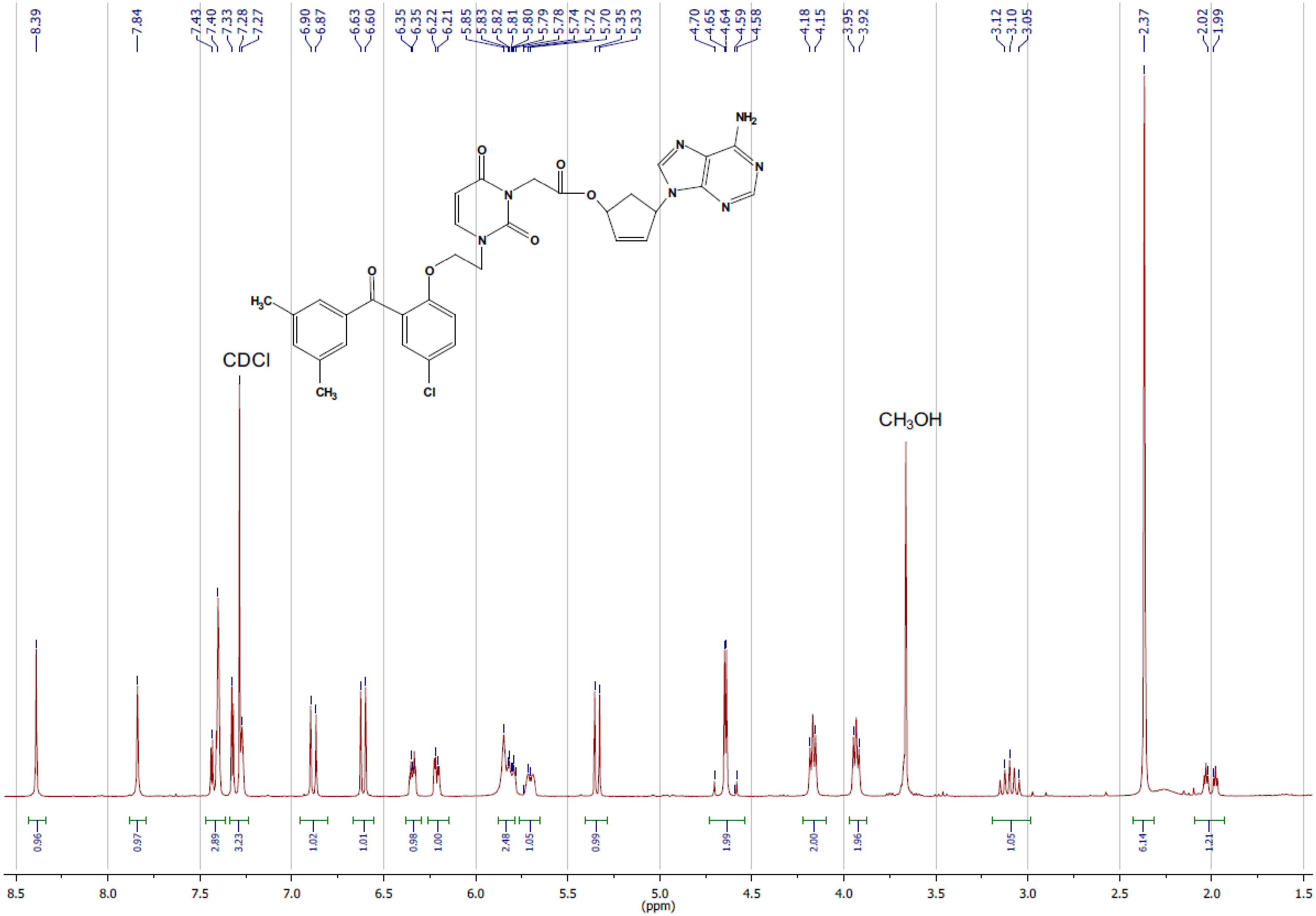

Figure S45. ^{1}H NMR spectrum of compound **6c** (in $CDCl_3$ at 400 MHz)

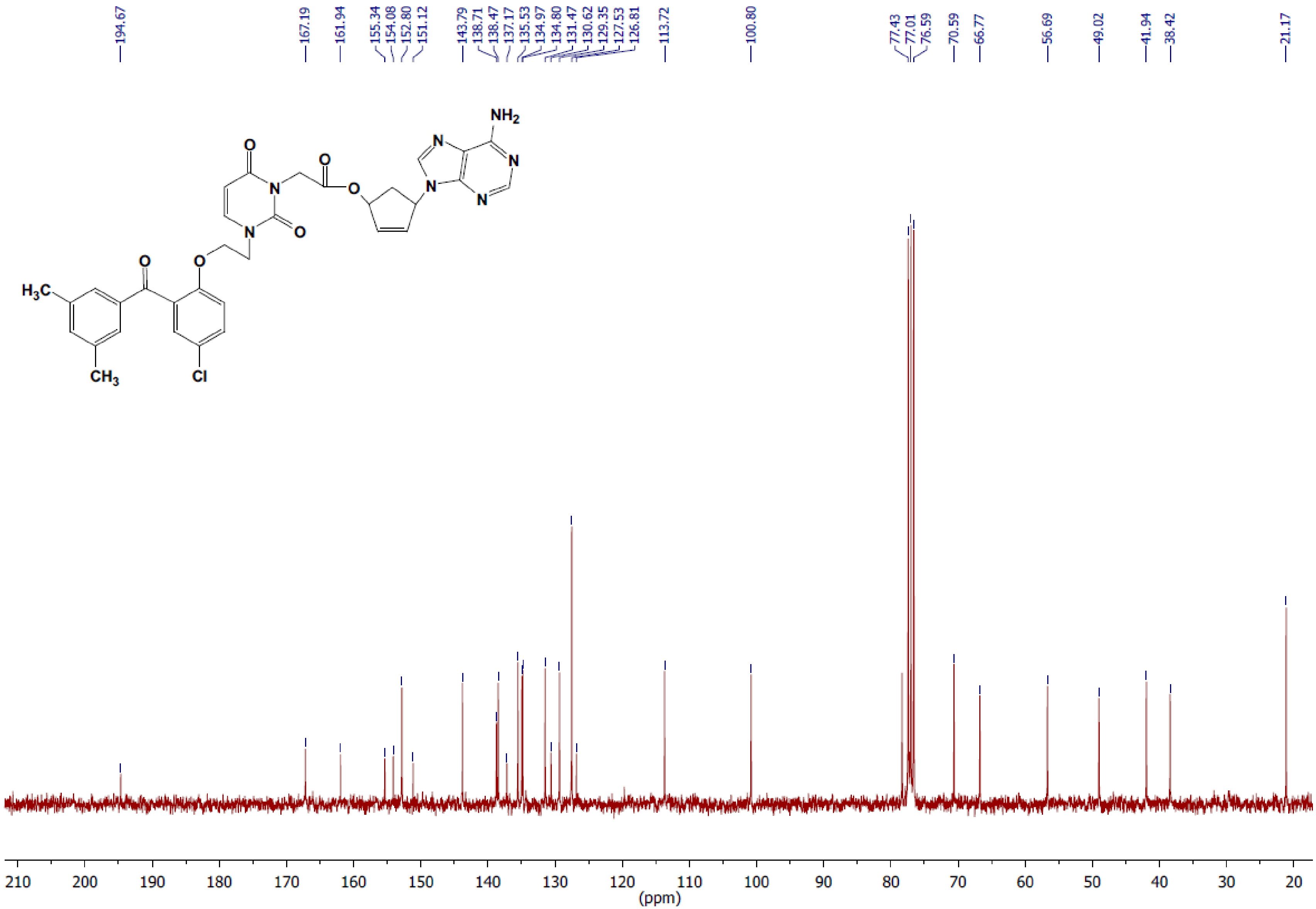

Figure S46. ^{13}C NMR spectrum of compound **6c** (in $CDCl_3$ at 100 MHz)

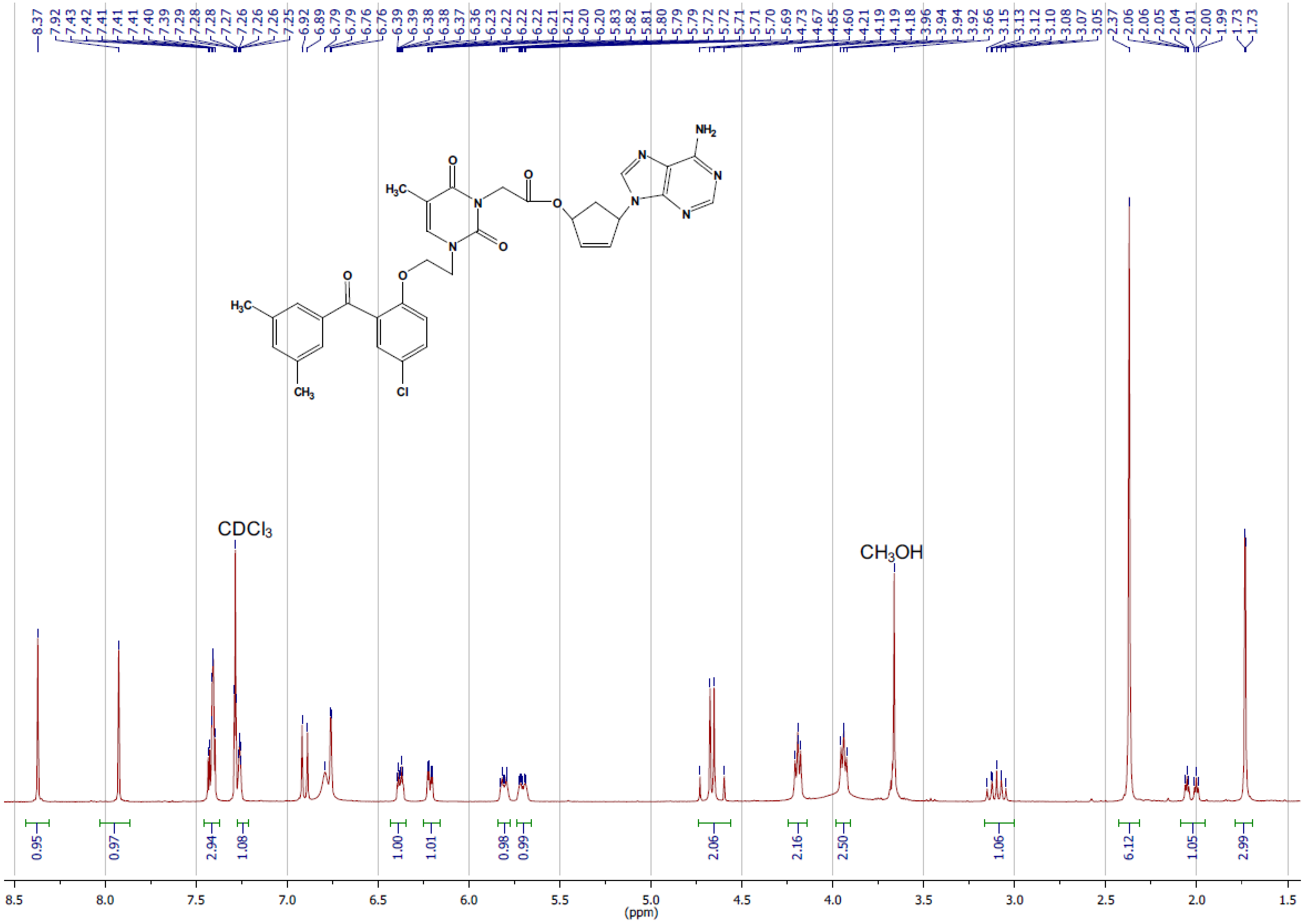

Figure S47. ^{1}H NMR spectrum of compound **6d** (in $CDCl_3$ at 400 MHz)

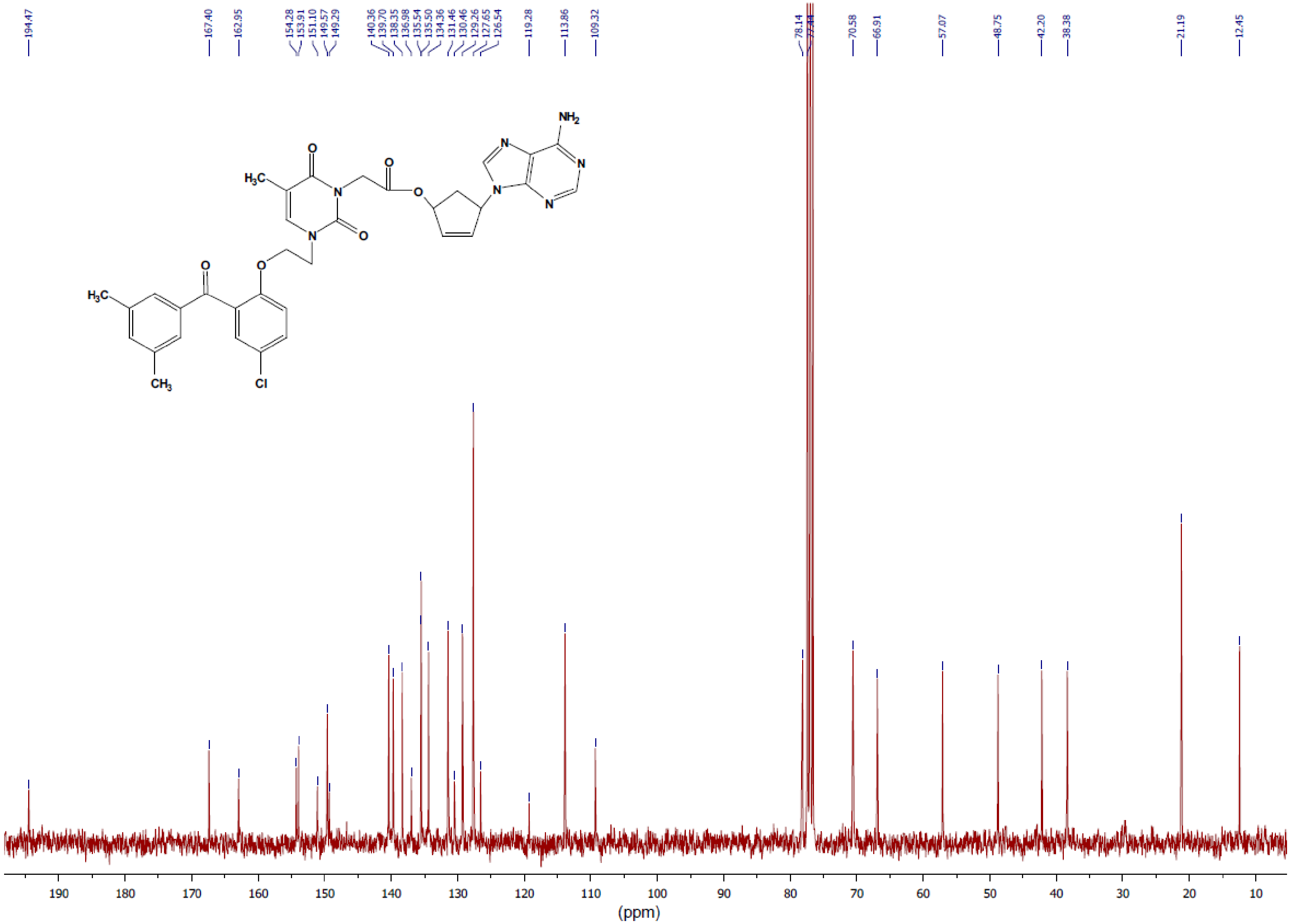

Figure S48. ^{13}C NMR spectrum of compound **6d** (in $CDCl_3$ at 100 MHz)

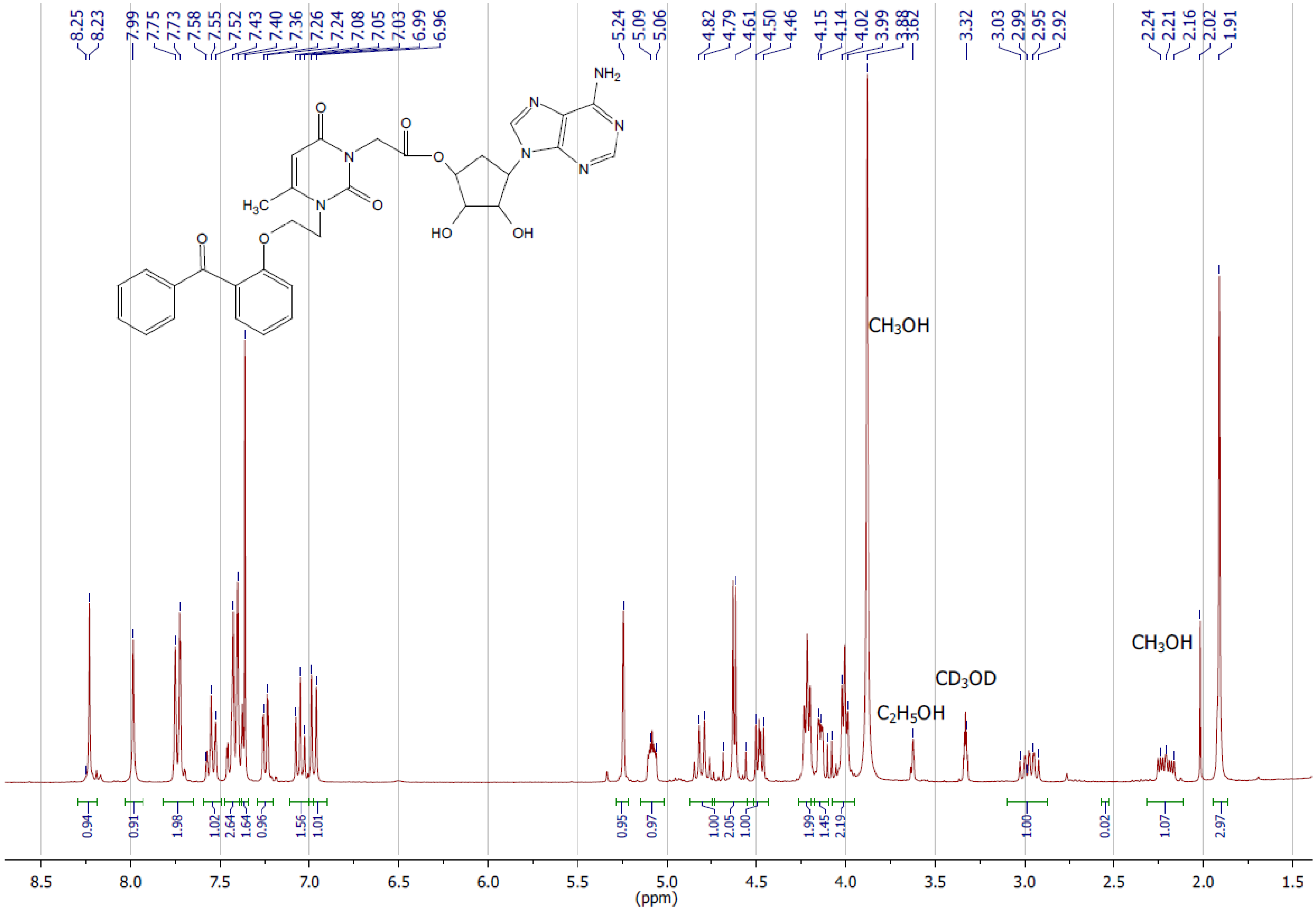

Figure S49. ^{1}H NMR spectrum of compound **3a** (in $CDCl_3$: CD_3OD at 400 MHz)

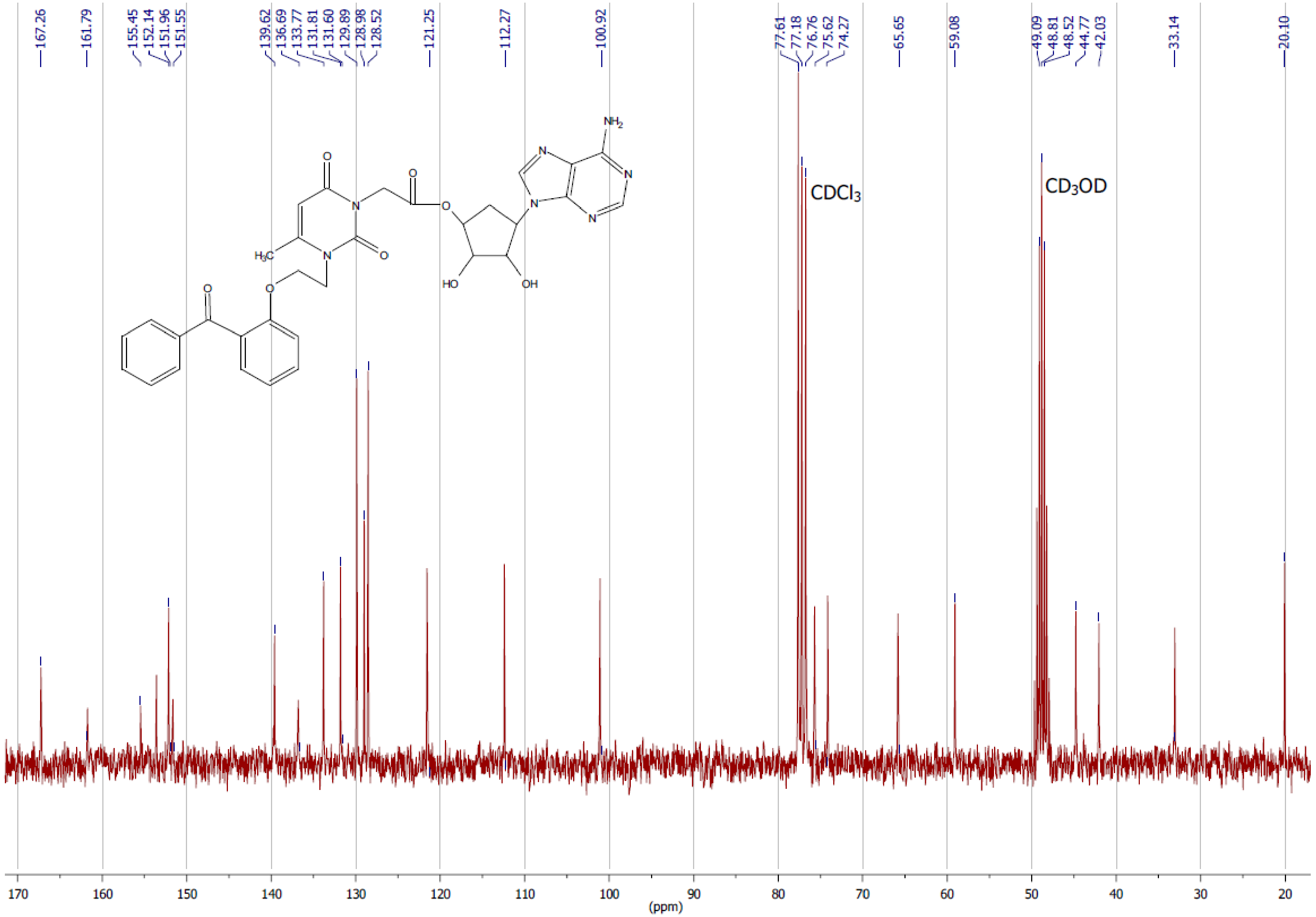

Figure S50. ^{13}C NMR spectrum of compound **3a** (in $CDCl_3$: CD_3OD at 100 MHz)

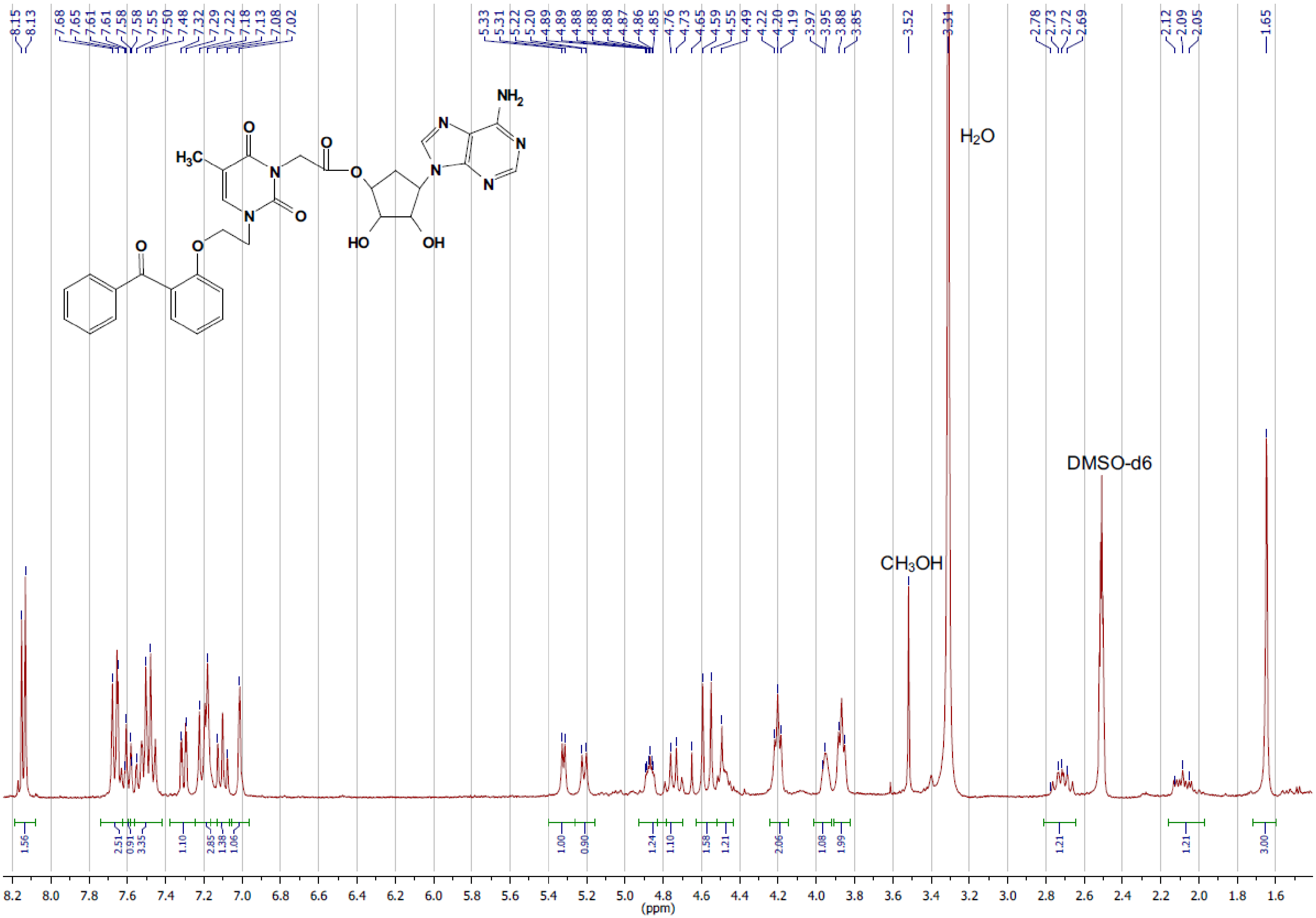

Figure S51. ^{1}H NMR spectrum of compound **3b** (in DMSO-d_6 at 400 MHz)

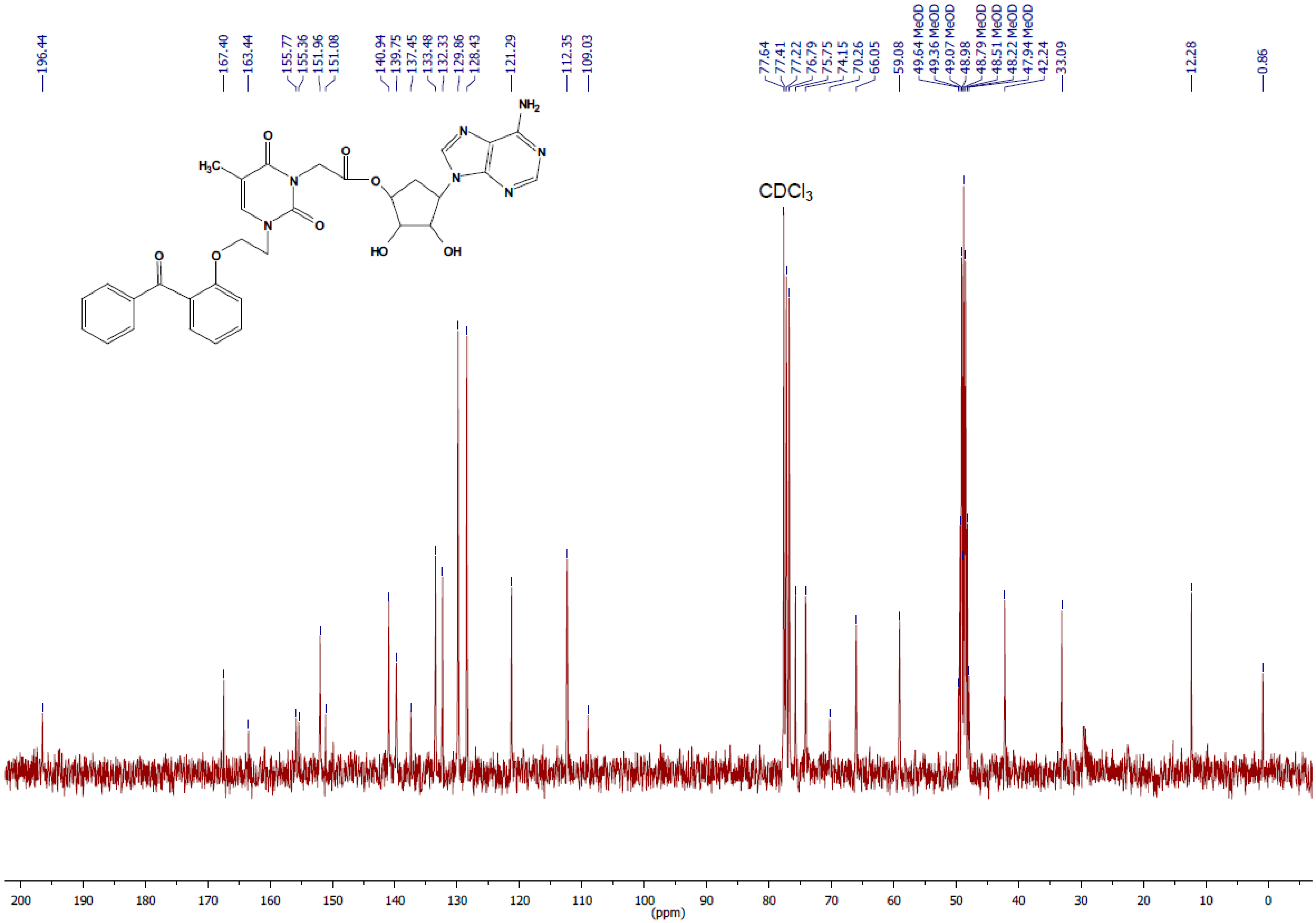

Figure S52. ^{13}C NMR spectrum of compound **3b** (in $CDCl_3$:CD_3OD at 100 MHz)

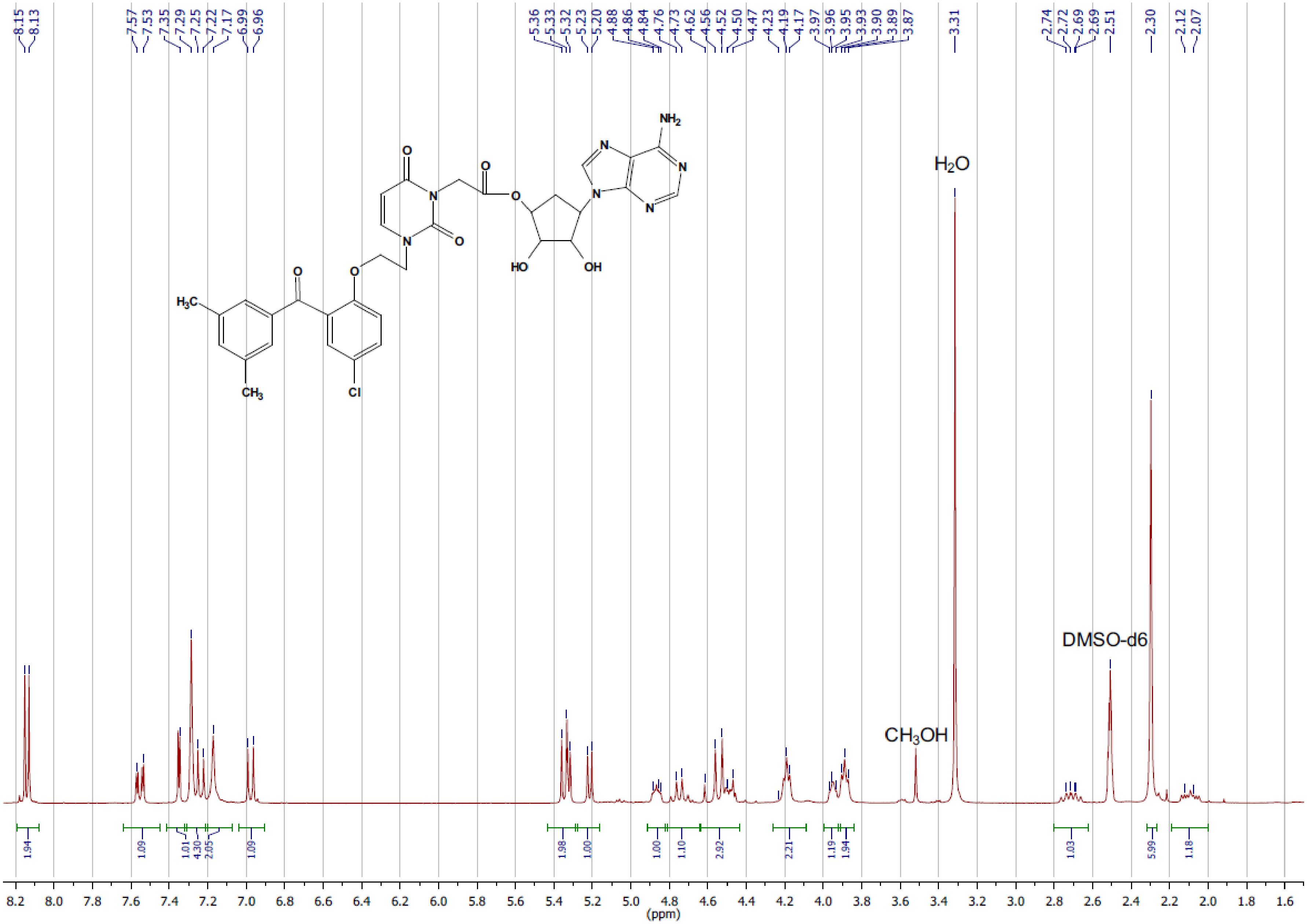

Figure S53. ^{1}H NMR spectrum of compound **3c** (in DMSO-d_6 at 400 MHz)

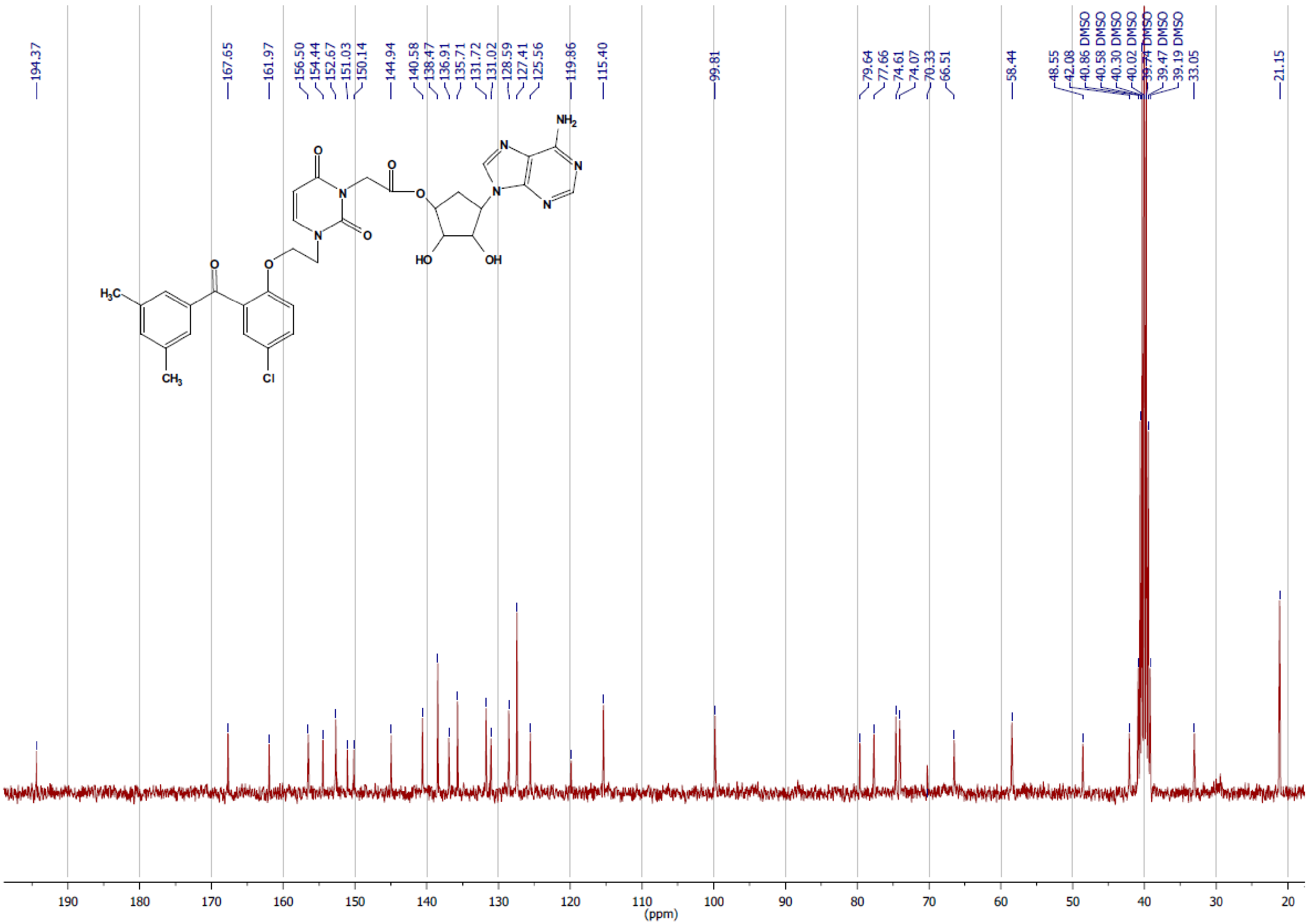

Figure S54. ^{13}C NMR spectrum of compound **3c** (in DMSO-d_6 at 100 MHz)

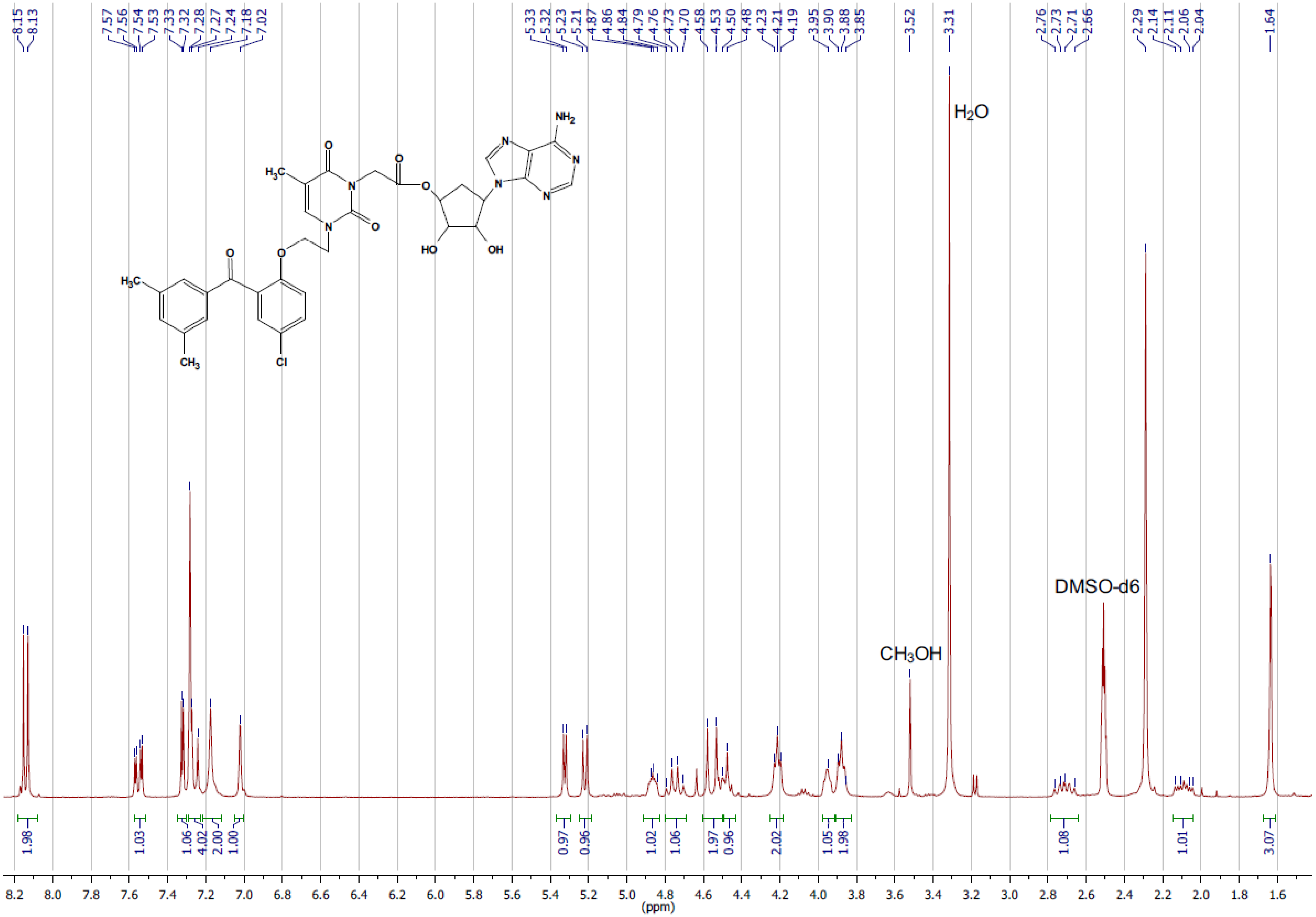

Figure S55. ^{1}H NMR spectrum of compound **3d** (in DMSO-d_6 at 400 MHz)

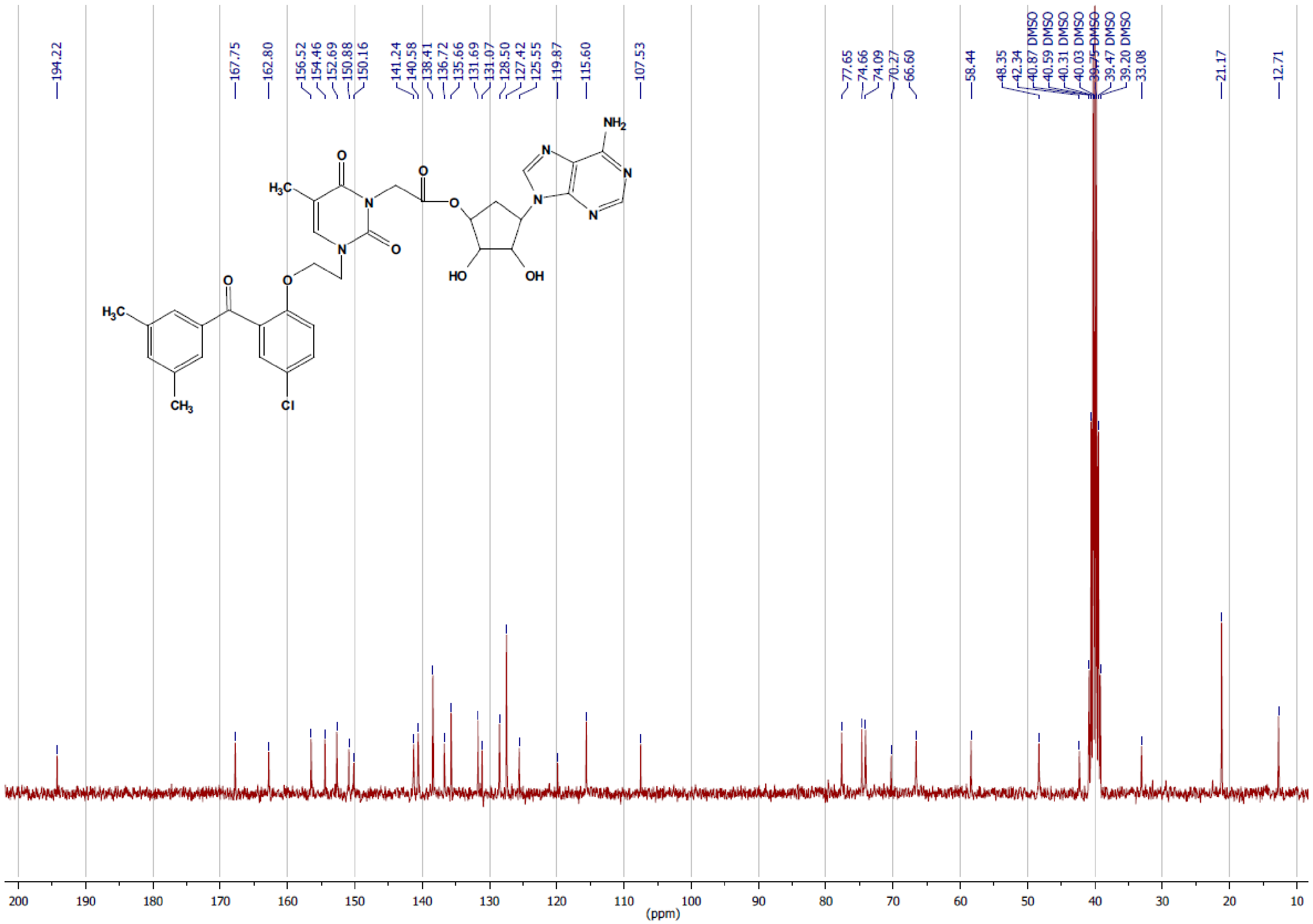

Figure S56. ^{13}C NMR spectrum of compound **3d** (in DMSO-d_6 at 100 MHz)
